# Safety of Tocilizumab in COVID-19 Patients and Benefit of Single-Dose: The Largest Retrospective Observational Study

**DOI:** 10.3390/pharmaceutics14030624

**Published:** 2022-03-11

**Authors:** Ayman M. Al-Qaaneh, Fuad H. Al-Ghamdi, Sayed AbdulAzeez, J. Francis Borgio

**Affiliations:** 1 Clinical Pharmacy Services Division, Pharmacy Services Department, Johns Hopkins Aramco Healthcare (JHAH), Dhahran 31311, Saudi Arabia; ayman.qaaneh@jhah.com; 2 Department of Genetic Research, Institute for Research and Medical Consultations (IRMC), Imam Abdulrahman Bin Faisal University, Dammam 31441, Saudi Arabia; asayed@iau.edu.sa; 3 Pharmacy Services Department, Johns Hopkins Aramco Healthcare (JHAH), Dhahran 31311, Saudi Arabia; fuad.ghamdi@jhah.com; 4 Department of Epidemic Diseases Research, Institute for Research and Medical Consultations (IRMC), Imam Abdulrahman Bin Faisal University, Dammam 31441, Saudi Arabia

**Keywords:** COVID-19 patient, tocilizumab, mortality, treatment, ICU admission, monoclonal antibody, infection

## Abstract

Severe acute respiratory coronavirus-2 (SARS-CoV-2) still presents a public threat and puts extra strain on healthcare facilities. Without an effective antiviral drug, all available treatment options are considered supportive. Tocilizumab as a treatment option has to date shown variable results. In this retrospective study, we aimed to assess predictors of mortality of COVID-19 patients (*n* = 300) on tocilizumab and the clinical effectiveness of this drug. The results showed that ICU admission *OR* = 64.6 (95% CI: 8.2, 507.4); age of the patient *OR* = 1.1 (95% CI: 1.0, 1.1); and number of tocilizumab doses administered by the patient *OR_(two doses)_* = 4.0 (95% CI: 1.5, 10.9), *OR_(three doses)_* = 1.5 (95% CI: 0.5, 5.1), and *OR_(four doses or more)_* = 7.2 (95% CI: 2.0, 25.5) presented strong correlation factors that may be linked to COVID-19 mortality. Furthermore, our study showed the beneficial effects of early administration of tocilizumab *OR* = 1.2 (95% CI: 1.1, 1.4) and longer hospital length of stay *OR* = 0.974 (95% CI: 0.9, 1.0) in reducing COVID-19 mortalities. High blood D-dimer concentration *OR* = 1.1 (95% CI: 1.0, 1.2) and reciprocal blood phosphate concentration *OR* = 0.008 (95% CI: 0.0, 1.2) were correlated to high mortality under SARS-CoV-2 infection. The short-term effect of a single dose of tocilizumab was a significant increase in blood BUN and liver enzymes (ALT, AST, and LDH) above their normal ranges. Furthermore, it significantly reduced CRP blood concentration, but not to normal levels (13.90 to 1.40 mg/dL, *p* < 0.001). Assessing the effect of different doses of tocilizumab (in terms of the number of doses, total mg, and total mg/kg administered by the patients) indicated that administering more than one dose may lead to increases in ICU length of stay and hospital length of stay of up to 14 and 22 days after the last dose of tocilizumab (6 to 14, *p* = 0.06, and 10 to 22, *p* < 0.001), with no improvement in 28- and 90-day mortality, as confirmed by Kaplan–Meier analysis. There were also clear correlations and trends between the number of doses of tocilizumab and increased blood CO_2_, MCV, RDW, and D-dimer concentrations and between number of doses of tocilizumab and decreased CRP, AST, and hemoglobin concentrations. Microbiology analysis showed a significant increase in the incidence of infection after tocilizumab administration (28 to 119, *p* < 0.001) with a median time of incidence within 6 days of the first dose of tocilizumab. A significant correlation was also found between the number of tocilizumab doses and the number of incidences of infections after tocilizumab administration r (298) = 0.396, *p* = 1.028 × 10^−12^. Based on these results and depending on the pharmacokinetic parameters of the drug, we recommend single-dose administration of tocilizumab as the optimal dosage for COVID-19 patients who do not have active bacterial infection or liver diseases, to be administered as soon as the patient is admitted to the hospital.

## 1. Introduction

The newly emerged coronavirus infection disease (COVID-19) caused by the severe acute respiratory syndrome coronavirus-2 (SARS-CoV-2) started in Wuhan, China, in December 2019, and came to affect all countries. By October 5, 2021, almost 235 million confirmed cases had been reported, including 4.8 million deaths worldwide, with a calculated rate of mortality of around 2.04% [1]. A meta-analysis aimed at assessing the fatality rate among hospitalized COVID-19 patients revealed 11.5% (95% CI 7.7–16.9) and 40.5% (95% CI 31.2–50.6) mortality for general patients (excluding critical care studies) and critical care patients, respectively [2]. COVID-19 clinical manifestations range from being asymptomatic to mild and moderate symptoms [3,4]. However, in some patients, symptoms may progress to severe complications that may require hospitalization and ICU admission. Moreover, in critical cases, patients may develop multiple organ failures and need to be put on mechanical ventilation, which may eventually end with death [5]. A systemic hyperinflammatory state that causes a severe course of the disease is called a “cytokine storm” and is defined as an aggressive inflammatory immune response characterized by releasing large amounts of proinflammatory mediators known as cytokines (i.e., Interleukins (IL) 1α, 1β, 1ra, IL 2, 2R, 2Ra, IL 3, IL 6, IL 7, IL 8, IL 9, IL 10, IL 15, IL 12p40, 12p70, IL 13, IL 18), which are also identified in a higher concentration in deceased patients compared to survivors [6,7,8,9,10,11,12,13,14]. These cytokines facilitate the entrance of a large amount of fluid into the alveoli, causing dyspnea and respiratory failure by increasing vascular permeability [15]. Of these ILs, IL 6 and IL 10 were directly correlated with serum C-reactive protein (CRP) and lactate dehydrogenase (LD) values [7]. On the other hand, IL 6 has been frequently reported to be associated with poor prognosis and increased risk of mortality [8]. The role of IL 6 in the cytokine storm in COVID-19 patients has been attributed to its ability to bind to membrane IL 6 receptors and enhance the production of CRP and fibrinogen. Furthermore, IL 6 binds to soluble IL 6 receptor, forming Hyper IL 6, which has the potential to activate all kinds of cells, presenting a focal role in the cytokine storm [11,16]. In a meta-analysis that included six studies (*n* = 1798), Coomes and Haghbaya reported a 2.9-fold increase in blood IL 6 in patients with the complicated disease compared with those with the uncomplicated disease [17]. Accordingly, inhibition of IL 6 was proposed as a promising therapeutic option for managing disturbed immune response in COVID-19 patients [17]. Pharmacologically, tocilizumab is an anti-IL 6 receptor monoclonal antibody that binds both the soluble and membrane-bound forms of the IL 6 receptor [18,19]. Tocilizumab was not granted Food and Drug Administration (FDA) authorization for emergency use in the treatment of COVID-19 patients until 24 June 2021 [20]. Before that authorization, healthcare facilities, including Johns Hopkins Aramco Healthcare (JHAH), used tocilizumab to treat COVID-19 patients based on clinical trial recommendations or hospital/national guidelines. Although many studies have evaluated the effect of tocilizumab in the treatment of COVID-19 patients, the outcomes of these studies have been inconsistent, and the majority of them were limited to a small sample size. Further details are presented in Table S1. It is also important to study the factors associated with better or poor outcomes and early markers of prognosis of COVID-19 patients for whom starting tocilizumab is intended as part of their therapy protocol.

This retrospective observational study aimed to (i) identify the baseline clinical characteristics and laboratory parameters that could correlate with mortality in COVID-19 patients on tocilizumab; (ii) assess the effect of a single dose of tocilizumab on different laboratory parameters in COVID-19 patients; (iii) compare the effect of different doses of tocilizumab on various clinical and laboratory-related parameters in COVID-19 patients; (iv) investigate the effect of tocilizumab on the possibility of increasing serious infections in COVID-19 patients.

## 2. Material and Methods

### 2.1. Study Design and Procedures

An electronic health records system (EPIC^®^) was retrospectively screened for COVID-19 patients admitted to Johns Hopkins Aramco Healthcare (JHAH) between 1 May 2020 and 31 May 2021 who received tocilizumab as part of their COVID-19 treatment protocol. Inclusion criteria were: 1. adult patients ≥ 18 years; 2. admitted directly to JHAH because of SARS-CoV-2 infection confirmed by RT-PCR (real-time polymerase chain reaction) assays on nasopharyngeal swabs. Exclusion criteria were: 1. negative RT-PCR result for SARS-CoV-2 infection in two consecutive samples taken at least 48 h apart; 2. transfer from another healthcare facility before being admitted to JHAH; 3. discharge to another healthcare facility. All patients were followed up until discharge or death. The study was approved by the JHAH institutional review board and registered under IRB#13-20 (date of approval 23 June 2020) with a waiver of consent and followed the principles specified in the Declaration of Helsinki. Upon hospital admission, all COVID-19 patients received a baseline therapy per individual case scenario as determined by the primary team and according to the Ministry of Health therapeutic protocols. Tocilizumab was used on a case-by-case basis for patients exhibiting symptoms of cytokine storm as described below based on criteria set by the drug and therapeutics committee when assessed by the treating team; hence, IL 6 tests were not conducted at JHAH.

Patients were eligible for tocilizumab if they showed elevation in the inflammatory parameters, defined as one of the following: increase in CRP level by 50–100% of baseline or normal upper limit (<1.0 mg/dL), increase in the blood ferritin level by 100% of baseline or normal upper limit (21.81–274.66 ng/mL), or D-dimer blood concentration more than 0.7 mg/L FEU (0.0–0.7 mg/L FEU). Tocilizumab was administered as a 4 to 8 mg/kg intravenous (IV) dose using actual body weight with a maximum dose of 800 mg. Doses were rounded to 400 mg, 600 mg, or 800 mg. Patients were eligible for a second dose only (after 12–24 h of the first dose) if they remained febrile despite treatment. However, some treating physicians were noncompliant with the prescribing criteria of the tocilizumab.

### 2.2. Data Collection

Data collected included demographic data, smoking status, comorbidities before hospital admission, concurrent medication use during COVID-19 hospitalization, hospital-related parameters, intensive care unit (ICU)-related parameters, tocilizumab-related parameters, laboratory results (electrolytes, chemical profile, cardiac profile, hepatic profile, iron/anemia profile, C-reactive protein, blood profile, coagulation profile, and urine profile), and coinfections and culture results (Further details are presented in Table S2). All extracted data were stored in electronic format using a Microsoft Excel sheet and retrospectively analyzed. Comorbid conditions were defined as clinical conditions recorded in the EPIC system before current hospital admission. Laboratory baseline data were defined as the most recent data before tocilizumab administration and after hospital admission. Laboratory data after tocilizumab administration were defined as the most recent lab results before discharge and after the last dose of tocilizumab. Day 0 was defined as the first day tocilizumab was administered. Infection after tocilizumab was defined as a positive microbiologic test that required antibiotic administration for the identified pathogen.

### 2.3. Outcomes Measures

The primary endpoint was to investigate risk factors affecting all causes of mortality of COVID-19 patients on tocilizumab who were discharged dead. Other endpoints evaluated included clinical effectiveness and short-term effects and side effects of single and multiple doses of tocilizumab in COVID-19 patients. Another endpoint was to assess the prevalence and type of infections after tocilizumab administration and before discharge.

### 2.4. Statistical Analysis

The categorical variables are presented as absolute number and percent. Continuous variables are presented as mean ± standard deviation (SD) or median values with interquartile range (IQR), depending on their distribution. Differences of the studied categorical variables between survivors and nonsurvivors COVID-19 patients and between patients who received different doses of tocilizumab were assessed by the chi-square test of independence or Fisher’s exact test as appropriate. The chi-square goodness-of-fit test was used to assess the difference in the studied categorical variables between survivor/nonsurvivor COVID-19 patients vs. a preidentified value. The differences of the studied continuous variables between survivors and nonsurvivors COVID-19 patients were assessed by independent sample *t*-test for parametric data and Wilcoxon rank-sum test (Mann–Whitney U test) for nonparametric data as appropriate. McNemar or Stuart–Maxwell tests were used to assess the differences in the studied categorical variables before and after tocilizumab administration as appropriate. The differences in the studied continuous variable before and after tocilizumab administration were assessed by paired sample *t*-test for parametric data, Wilcoxon signed ranks test for nonparametric data with symmetric distribution, and sign test for nonparametric data when the assumption of symmetry was violated, as appropriate. The differences in the studied continuous variables between patients who received different doses of tocilizumab were assessed by one-way ANOVA for parametric equivariance data and Welch’s test for parametric nonequivariance data as appropriate. In contrast, the Kruskal–Wallis test was used for nonparametric studied continuous data. In univariate analysis, binary logistic regression analysis was used for each studied parameter over the binary outcomes (survival/nonsurvival). Each predictor for mortality with a *p*-value < 0.1 was fitted in one of two hypothesized model sets for multivariable regression analysis depending on its category. The model A set contained demographical data, clinical characteristics, concurrent drug administration during COVID-19 hospitalization, time-related parameters, and tocilizumab-related parameters. In contrast, the model B set contained laboratory results only. Collinearity between independent variables was checked using correlation coefficients in a correlation matrix. Independent variables with absolute correlation coefficients ≥0.7 were considered strongly correlated, and one of them was excluded. The linear relationship between any continuous predictor and logit transformation of the outcome variable was checked by the Box–Tidwell test. For nonlinear relationships, polynomial transformation (x^2^) or inverse function (1/x) was implemented to ensure linearity as appropriate. Continuous predictors were tested for potential outliers (absolute standardized residual (z-score) > 3.29)). Outliers due to wrong entries were removed, but other outliers were utilized for model building, as the predictors presented normal distributions of data. However, analysis without outliers was conducted to assure no effect of the outliers in the model and to ensure the validity of the selected model. For multivariable binary logistic regression, the backward LR stepwise method was implanted and tested for its goodness of fit by the Hosmer–Lemeshow test. The results of the univariate and multivariate binary logistic regressions were presented as odds ratios (OR) with 95% confidence intervals (CIs). Models were compared using the −2log likelihood (−2LL) statistic, the Hosmer–Lemeshow test, and the adjusted R (Nagelkerke R^2^) test. The total accuracy of the models was also recorded. Akaike’s information criterion test (AIC) was calculated to compare different models and select that with the best fit. Models more than 2 AIC units lower than the subsequent model is considered significantly better. The Wald chi-square test was used to assess the contribution of each predictor in the model in the context of other predictors. Sensitivity, specificity, positive predictive value, negative predictive value, false-positive value, false-negative value, false-positive predictive value, and false-negative predictive value were calculated for the final fitted models. Survival analysis for COVID-19 patients was conducted via Kaplan–Meier curve. The significance of probability trends for different doses of tocilizumab in terms of the number of doses in groups was confirmed by log-rank test (Mantel–Cox). The correlation between dose groups and the number of incidences of infection after tocilizumab administration were assessed using Spearman’s rho correlation. All conducted tests were two-tailed and considered significant when the *p*-value was <0.05. No imputations were made for missing data points. All data used in the study were analyzed using SPSS 25.0 (IBM SPSS Statistics for Windows, Version 25.0 IBM Corp., Armonk, NY, USA).

## 3. Results

**Figure 1:**
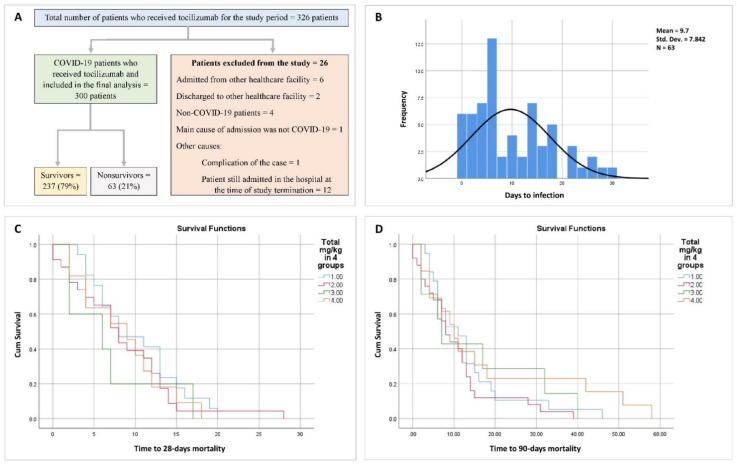
(**A**): Study scheme; (**B**): days to the first incidence of infection after the first dose of tocilizumab; (**C**): 28-day Kaplan–Meier survival curve integrating different dose groups of tocilizumab; (**D**): 90-day Kaplan–Meier survival curve integrating different dose groups of tocilizumab.

Out of 326 patients who received tocilizumab during the study period, 300 were included in the final analysis. Twenty-six patients were excluded because of transfer from other healthcare facilities, discharge to other health care facilities, not being COVID-19 patients, or the main cause of admission not being SARS-CoV-2 infection despite the patient receiving tocilizumab because of SARS-CoV-2 infection. Of the 300 COVID-19 patients on tocilizumab that were retrospectively analyzed, 237 survived (79%) and 63 (21%) were decreased. The study scheme is illustrated in Figure 1A. In addition to the extracted parameters mentioned in Table S2, additional parameters were calculated and utilized in the study (Table S3).

### 3.1. Predictors of Mortality in COVID-19 Patients

The majority of COVID-19 patients on tocilizumab were males (65.3%, *p* < 0.001). At the time of admission, the median weight and BMI of the patients were 83.00 kg and 31.36 Kg/m^2^, respectively. More than half of the patients (58.7%) were obese, with BMI > 35.0 Kg/m^2^. The majority of the patients were nonsmokers (78.3%, *p* < 0.001). Prior to hospital admission, there was a significant difference in the number of comorbidities between patients (*p* < 0.01), with 33.0% of patients reporting having three or more comorbid conditions. However, there was no significant difference in the number of patients who reported having no comorbid conditions or having one or two comorbid conditions (not shown, *p* = 0.526). The most-reported comorbidities were diabetes mellitus (DM) (51.0%), hypertension (31.3%), dyslipidemia (21.0%), cardiac diseases (15.7%), and G6PD disease (14.3%). Nearly all patients (99.3%) received at least one type of corticosteroid. More than half of the patients (53.7%) were admitted to the ICU, with median hospital and ICU lengths of stay being 14 and 11 days, respectively. Although most patients received a maximum of two doses of tocilizumab (76%), 24% received three or more doses, which was confirmed by cumulative mg of tocilizumab (>1600 mg) received by the patients (18.3%). The median time between hospital admission and the first dose of tocilizumab was three days. However, for patients who received the first dose of tocilizumab in the ICU, the median days between ICU admission and the first dose of tocilizumab was one day only. Hospital and ICU lengths of stay after the last dose of tocilizumab were 8 and 7 days, respectively. Clinical parameters and outcome measures of COVID-19 patients on tocilizumab are presented in Table S4, column 1.

Nonsurvivors were older (70 vs. 60, *p* < 0.001) and had a higher number of comorbid conditions (57.1% vs. 26.6%, for three or more comorbidities). There was no significant difference in the number of patients who reported having fewer than three comorbidities between the survivor and nonsurvivor groups (not shown, *p* > 0.05). However, there were significant differences between patients who reported having three comorbidities vs. no comorbidities (*p* < 0.001), one comorbidity (*p* < 0.004), and two comorbidities (*p* < 0.001). Furthermore, significantly higher numbers of patients reported having hypertension, cardiac diseases, DM, arthritis, cancer, and osteopenia/osteoporosis in the nonsurvivor group. Nonsurvivors had longer median hospital and ICU lengths of stay (19 and 14.5 vs. 13 and 9 days, *p* < 0.001 and *p* < 0.01, respectively), and the majority of them were admitted to the ICU (98.4% vs. 4.8%, *p* < 0.001) and received antiviral drugs (38.1% vs. 16.9%, *p* < 0.001). Nonsurvivors also received more tocilizumab doses (2 vs. 1, *p* < 0.001), with 39.7% of them receiving at least three doses of tocilizumab in comparison with 19.8% of survivors (*p* < 0.001). Total tocilizumab amounts in terms of mg and mg/kg were higher in the nonsurvivors as well (1400 mg vs. 800 mg, *p* < 0.001, and 16.7 mg/kg vs. 8.4 mg/kg, *p* < 0.001, respectively). The median time between hospital admission and the first dose of tocilizumab was longer in the nonsurvivors (4 vs. 2, *p* < 0.001). Although the median time between ICU admission and the first dose of tocilizumab for patients who received the first dose in the ICU was one day for both groups, nonsurvivors had a wider IQR (0–2 vs. 0–3, *p* < 0.05). There was no significant difference in the median hospital length of stay after the last dose of tocilizumab between both groups (*p* = 0.88). However, the median ICU length of stay after the last dose of tocilizumab was significantly longer in the nonsurvivor group (8 vs. 5, *p* < 0.05). In terms of inflammatory biomarkers, although the median blood ferritin level (1205.5 vs. 672.0, *p* < 0.01), D-dimer level (0.86 vs. 0.72, *p* < 0.01), and LDH level (403 vs. 344, *p* < 0.01) were significantly higher in the nonsurvivors, there was no significant difference in the median CRP level (15.5 vs. 16.0, *p* = 0.7). Further details and a comparison between the most recent lab results before tocilizumab administration of survivors and nonsurvivors are presented in (Table S4, columns 3 and 4). In a univariate logistic regression analysis (Tables S5 and S6), 28 variables had a significant effect (*p* < 0.1) on the mortality (12 variables from the model set A and 16 variables from the model set B) (Table 1) and were included in the multivariable logistic regression analysis models based on their corresponding categories. In multivariable logistic regression analysis (Tables S9 and S10), ICU length of stay, ICU length of stay after the last dose of tocilizumab, BUN, hematocrit, and MCH were excluded because of high collinearity with other factors as presented in correlation matrices (Tables S7 and S8). Furthermore, the variable “days to ICU admission from the hospital admission” was excluded because of the limited sample size and noninclusivity, as not all patients were admitted to the ICU (*n* = 159). Activated partial thromboplastin time (APTT) was excluded because of the limited number of patients (*n* = 119). However, a regression model was conducted using APTT to ensure that it did not affect the final regression model (not shown). Although the number of tocilizumab doses, number of tocilizumab doses categorized into four groups, total mg of tocilizumab administered by the patient, total mg of tocilizumab administered by the patient categorized into four groups, total mg/kg of tocilizumab administered by the patient, and total mg/kg of tocilizumab administered by the patient categorized into four groups are all showed high values of collinearity, all were utilized in multivariable logistic regression models, albeit one at a time (Table S9). All studied continuous variables showed linear relationships with logit transformation of odds of mortality except total tocilizumab in milligrams and blood phosphate concentration, which were linearized by a polynomial (*x*^2^) and reciprocal transformation (1/*x*), respectively. Models are presented in Tables S9 and S10. For model set A, all extracted models using different presentations of tocilizumab received by the patients fit our data, which was confirmed by the Hosmer–Lemeshow test (*p* > 0.05). Model II from model set A, using number of tocilizumab doses administered by patients categorized into four groups, showed the highest adjusted R^2^ value (Nagelkerke R^2^ = 0.524) and the lowest −2log likelihood value (−2LL = 185.330), which indicates that it best presented our data in reference to other models. AIC tests confirmed these results; the AIC value for model II was 201.33, and the difference (ΔAIC) between model II and its subsequent higher model (model IV AIC = 205.711) was > 2 AIC units, which indicated a significant difference between the two models (Table S11). The WALD test revealed that admission to ICU represented the highest correlated factor to mortality in COVID-19 patients on tocilizumab, followed by patient age, the number of tocilizumab doses categorized into four groups, days between hospital admission and the first dose of the tocilizumab, and hospital length of stay, in descending order. For model set B, one model was created for the laboratory results. In this model, COVID-19 mortality was most linked with blood dimer concentration, followed by reciprocal blood phosphate concentration. However, because of the limited number of patients that had results for blood phosphate concentration (*n* = 117), and as removal of this variable would lead to a vast increase in the number of patients included in the multivariable regression analysis (*n* = 114 to 271), a model was created without this variable (Table S12). The newly created model consisted of RDW, MCV, AST, D-dimer, platelets, and creatinine, arranged in descending order of their contribution to the mortality. To confirm model selection and the effect of outliers on the selected models, analysis was reconducted after removing the potential outlier values (*n* = 19 and 43 for model sets A and B, respectively), which revealed the same model variables in the model set A (Tables S13 and S14). For model set B, removing outliers shortened the variable list to blood phosphate concentration only (Table S15). As the blood phosphate concentration was the only variable in the model, removing it led to establishing a new model consisting of RDW, creatinine, MCV, and platelets, arranged in descending order based on their contribution (Table S16). While all the variables tended to increase the probability of mortality in COVID-19 patients on the tocilizumab, hospital length of stay had the opposite direction (OR 0.960 (0.925–0.995)). Sensitivity, specificity, positive predictive value, negative predictive value, false-positive value, false-negative value, false-positive predictive value, and false-negative predictive value for the final fitted models (with outliers) were calculated to be 56%, 92%, 66%, 87%, 8%, 44%, 34%, and 12%, respectively, for model set A and 88%, 75%, 21%, 99%, 25%, 13%, 76%, 1%, and 41%, respectively, for model set B.

Based on the extracted model, the equation for the probability of mortality for COVID-19 patients on tocilizumab for model set A can be represented by:

Ln (*P*/1 − *P*) = −10.40 + 4.17 *ICU* + 0.07 *AGE* + *TCZ* + 0.178 *DHT* −0.026

where: *P*—probability of mortality of COVID-19 patients on tocilizumab; *ICU*—0, for patients not admitted to ICU, or 1, for patients admitted to ICU; *AGE*—patient age in years; *TCZ*—tocilizumab dose category factor: 0 for patients who received one dose, 1.39 for patients who received two doses, 0.43 for patients who received three doses, or 1.98 for patients who received four or more doses; *DHT*—days between hospital admission and the first dose of tocilizumab; *HLS*—total hospital length of stay in days.

The equation for the probability of mortality for COVID-19 patients on tocilizumab for model set B (with blood phosphate concentration) can be represented by:

Ln (*P*/1 − *P*) = 0.096 + 0.117 *DD* − 4.891 (1/*PH*)

where: *P*–probability of mortality of COVID-19 patients on tocilizumab; *DD*—D-dimer blood concentration (mg/L); *PH*—blood phosphate concentration (mg/dL).

The equation for model set B (without blood phosphate concentration) can be represented by:

Ln (*P*/1 − *P*) = −8.73 + 0.230 *RDW* + 0.051 *MC*V + 0.007 *AST* + 0.053 *DD* −0.004 *PT* + 0.173 *CR*

where: *P*—probability of mortality of COVID-19 patients on tocilizumab; *RDW*—red corpuscular distribution width (%); *MCV*—mean corpuscular volume (fL); *AST*—blood aspartate aminotransferase concentration (IU/L); *DD*—D-dimer blood concentration (mg/L); *PT*—blood platelet concentration (K/µL); *CR*—blood creatinine concentration (mg/dL).

### 3.2. Short Term Effect of a Single Dose of Tocilizumab in COVID-19 Patients

The short-term effect of tocilizumab on different laboratory biomarkers was assessed using a cohort of patients who received a single dose of tocilizumab (400–800 mg) by comparing the most recent lab results before tocilizumab administration vs. the most recent lab results before discharge (Table 2). However, as there was a dramatic change in lab results at the end of patients’ lives, the same parameters were assessed for patients who were discharged alive (Tables S17 and S18).

### 3.3. Clinical Effectiveness of Different Doses of Tocilizumab in COVID-19 Patients

The baseline characteristics (demographics, number of comorbidities, concurrent drug administration, the most recent lab results before tocilizumab administration, admission to ICUs, days to ICU admission, days to the first dose of tocilizumab, days between ICU admission and the first dose of tocilizumab) and clinical effectiveness of tocilizumab (% of mortality, total hospital length of stay, total ICU length of stay, ICU length of stay after the last dose of tocilizumab, hospital length of stay after the last dose of tocilizumab) were compared among patients received different doses of tocilizumab (Table 3 and Table S19). Patients were categorized into four groups (one, two, three, and four or more) based on the number of tocilizumab doses administered. Patients who received more doses had higher total mg and mg/kg of tocilizumab (*p* < 0.001) and higher ICU admission rates (100% for patients who received four doses or more, *p* < 0.001). Regarding baseline characteristics, patients who received more doses were significantly older (*p* < 0.001, pairwise compression not conducted). Furthermore, patients who received four doses or more of tocilizumab had a higher BUN concentration above the normal range, with a significant difference between groups (*p* < 0.01). For hepatic lab results, all groups showed normal median hepatic enzyme concentrations (ALK, ALT, and AST, *p* > 0.05) except for LDH, which was higher than the normal upper range in the four groups, with a significant difference between them (*p* < 0.001). There were no differences in the inflammatory biomarkers (blood CRP, ferritin, and D-dimer concentrations) between different groups who received different doses of tocilizumab, except in LDH, as discussed above. However, all were above normal except for D-dimer concentrations for patients who received two doses. Although, there was a significant difference among groups in terms of blood creatinine level (*p* < 0.05), MCV (*p* < 0.01), MCH (*p* < 0.05), and platelet count (*p* < 0.001), all were within the normal ranges. All groups showed levels of lymphocytopenia, glucose in the urine, and abnormal urine urobilinogen concentration, with significant differences between groups before tocilizumab administration. There were no differences in the median days to ICU admission or days to the first dose of tocilizumab from hospital and ICU admission. All collectively indicated that the four groups had approximately the same baseline characteristics (except for age and ICU admission). Clinical outcome assessment showed that patients who received more doses of tocilizumab scored a higher percentage of mortality (50% for patients who received four doses, *p* < 0.001) and had longer hospital lengths of stay after the last dose of tocilizumab (up to 17 days for patients who received four doses, *p* < 0.001). However, there was no significant difference in the ICU length of stay after the last dose of tocilizumab, although the patients who received more doses had longer median ICU length of stay after the last dose (7, 8.5, 8.5, and 17 days for groups I, II, III, and IV, respectively, *p* > 0.05). Nevertheless, there was a significant difference in the total ICU length of stay between groups. Patients who received more doses had longer median ICU length of stay (0, 3.5, 7.5, and 18 days for groups I, II, III, and IV, respectively, *p* < 0.001). Kaplan–Meier curves for 28- and 90-day survival analysis after the last dose of tocilizumab for different dose groups of COVID-19 patients are illustrated in (Figure 1C,D). Using the Mantel–Cox log-rank test, no significant difference was observed between the groups (*p* = 0.32 and *p* = 0.75 for 28-day and 90-day survival, respectively), although patients who received four or more doses of tocilizumab scored the highest mean 90-day survival. Furthermore, no trend was observed in the 28-day or 90-day survival analysis between different dose groups of tocilizumab (Table S20).

### 3.4. Short-Term Effect of Tocilizumab on Different Lab Results

The short-term effect of different doses of tocilizumab on the lab results was evaluated by comparing the most recent lab results before discharge and after the last dose of tocilizumab between different dose groups (Table 4). There were no trends (increase or decrease) observed in the lab results with the increase in the number of doses of tocilizumab administered by the patient except hemoglobin concentration (**↓**), hematocrit percent (**↓**), MCHC (**↓**), prothrombin time (**↑**), and D-dimer concentration (**↑**). However, the difference between groups was significant for hemoglobin and D-dimer concentrations only (*p* < 0.05 and *p* < 0.001, respectively). Furthermore, although there were observed trends (increases or decreases), all labs were still within their normal ranges except for hemoglobin and D-Dimer concentrations. It is worth mentioning that at the baseline, no trend was observed for these lab results (hemoglobin, hematocrit, MCHC, prothrombin time, and D-dimer concentration), nor significant differences between groups (*p* = 0.93, 0.94, 0.71, 0.75, and 0.29, respectively). As presented for patients who received one dose of tocilizumab, because of dramatic changes at the end of patients’ lives, the comparison between different dose groups of tocilizumab for its short-term effect on different laboratory results was assessed only between patients who were discharged alive (Table S21). The following trends were observed: (**↑**) CO_2_ concentration, (**↓**) AST concentration, (**↓**) CRP concentration, (**↑**) MCV, (**↑**) RDW, (**↑**) mean platelet volume, and (**↑**) platelet distribution width. These trends were all significant except for mean platelet volume and platelet distribution width. However, these trends were not observed at the baseline, nor were they significant between groups (except for MCV; no trend but significant *p* < 0.01). With the observed trends, all results were still within their normal ranges except for CO_2_ and CRP concentration. The observed trends and the comparisons between groups (results at the baseline vs. results at the time of discharge for all patients vs. results at the time of discharge for patients who were discharged alive) are presented in Table 5.

### 3.5. Microbiology Assessment

Microbiology assessment of the number of patients who had an infection before and after tocilizumab administration revealed 27 (9%) and 64 (21.3) patients, representing 28 and 119 incidences, respectively (Table S22). Furthermore, the distribution of infections before and after tocilizumab administration based on their anatomical locations are presented in Table S23.

Details analysis of microorganisms revealed that the most common microorganisms before tocilizumab administration were *Candida albicans* (4), MRSA (methicillin-resistant *Staphylococcus aureus*) (4), and *Escherichia coli* (3). The most common microorganisms after tocilizumab administration were *Candida albicans* (33), *Staphylococcus epidermidis* (9), *Candida tropicalis* (8), and MRSA (7). Types of infection and detailed microorganisms before and after tocilizumab administration are listed in Tables S24 and S25. Furthermore, detailed microorganisms before and after tocilizumab based on their anatomical locations are presented in Table S26. The correlation between dose groups and the number of bacterial infections after tocilizumab administration was assessed using Spearman’s rho correlation, which showed a positive correlation with correlation coefficient r (298) = 0.396, *p* = 1.028 × 10^−12^. Analysis of the time to the first incidence of infection after the first dose of tocilizumab (*n* = 62) revealed a range of 0 to 30 days, with median days to the first infection of 6 (4–15) and mode days to the first infection of 6 (Figure 1B).

## 4. Discussion

Many studies have discussed clinical characteristics and predictors of mortality in COVID-19 patients worldwide [21,22,23,24,25,26] and in Saudi Arabia specifically [27,28,29]. However, our study was one of the few that assessed these predictors in COVID-19 patients receiving tocilizumab [30,31,32] and the first one in Saudi Arabia. Furthermore, our study was considered the largest retrospective study in terms of the number of patients (300 patients), and the largest study ever in the number of assessed parameters (74 group variables with 107 individual variables), that investigated the predictors of mortality in COVID-19 patients on tocilizumab. It was also the first study that assessed the clinical effectiveness, short-term effects, and the outcomes of multiple doses of tocilizumab (three and more doses) in COVID-19 patients.

Hypercytokinemia and its associated hyperinflammatory and immunodeficiency are reported to be responsible for lethal complications and increased mortality in COVID-19 patients [6,33,34,35,36]. Accordingly, the findings of this study provide valuable detail to guide the use of tocilizumab in COVID-19 patients.

Although male patients represented a higher proportion of the nonsurvival groups, there was no significant difference in terms of gender. However, this observation was consistent with many studies [31,32,37,38]. Among studies that assessed the mortality of COVID-19 patients on tocilizumab in Saudi Arabia, our study reported an overall mortality of 21%. In comparison, other studies reported 16.1% (*n* = 62) 28-day mortality [36]. In terms of mortality among ICU patients, the current study reported 38.5%, while other studies reported 31.1% (*n* = 61) [37] and 18.9% (*n* = 37) [38] mortality. However, worldwide, there is inconsistency in the percent of mortality of COVID 19 patients on tocilizumab, which in some studies has been reported to be as low as 13.4% (Asian-Indian) and up to 43.2% (American) [31,32,37,38]. This indicates the presence of many factors that can affect the response to the drug (e.g., pharmacogenomics, number of doses, age of the patients, and different guidelines for proper dosing and time of drug administration). Furthermore, the current study revealed that the mortality of COVID-19 patients on tocilizumab was higher in older patients, or that tocilizumab was less beneficial to the elderly than to younger patients, which was consistent with other studies [32,37]. In contrast, other studies reported nonsignificant differences in age between survivor and nonsurvivor COVID-19 patients on tocilizumab, though the nonsurvivors were older [30,39]. The poor responses of the old-aged patients may be attributed to physiological changes in their bodies hindering their ability to respond properly to the infection (e.g., decreased T cell production). On the other hand, as the patient age progresses, comorbidities’ prevalence increases dramatically [40]. Though our study results illustrated that the nonsurvivor group had a significantly higher number of patients who reported having hypertension, cardiac diseases, DM, arthritis, cancer, and osteopenia/osteoporosis, other studies reported DM and cancer only [31,32], while other studies found no significant differences in comorbidities between survival and nonsurvival patients [30]. The current study’s results indicated that admission to ICUs increased the probability of death due to COVID-19 infection, which was not seen by other studies [30,32]. However, some studies excluded patients admitted to ICUs from their analysis [39]. Results from our study indicated that nonsurvivors had longer median hospital and ICU lengths of stay, which was opposite to what was reported by Morrison et al., [30] who indicated that survivor patients had significantly longer HLS than nonsurvivors. Regarding concurrent drug administration with tocilizumab, our results showed that more patients received antiviral medications in the nonsurvival group than in the survival group, which was not seen in the other studies [30,32]. Furthermore, it is worth mentioning that in both studies, the use of antiviral drugs was before tocilizumab administration, and there was no information regarding whether the patients continued using these drugs concurrently with tocilizumab [30,32]. Nonsurvivors received more tocilizumab doses in terms of the number of doses, total mg administered, and total mg/kg, with 39.7% of them receiving at least three doses compared with 19.8% in the survivors’ group. None of the screened research mentioned using more than two doses of tocilizumab for comparison [31,32,38], except one study in which 2/120 patients received three doses [32] which makes the comparison difficult. However, in our study, subgroup analysis for patients who received one dose vs. patients who received two doses showed a significant increase in mortality in the latter group (*p* = 0.000044, results not shown), which was not significant in the Morrison et al. study [30]. Furthermore, although our study showed that patients who did not survive received more doses of tocilizumab in terms of total mg/kg, Morison et al., [30] reported no difference in the median cumulative mg/kg between groups. Moreover, in our study, the mean dose of tocilizumab in terms of mg/kg for patients who received one dose only was 6.32 (1.888) in comparison with the median first dose of 6.8 (5–7.8) reported by Morison et al. However, there was no difference in the mg of the first dose between both studies, as both our patients who received one dose and those who received the first dose in the Morrison cohort were given a dosage of 800 mg [30]. The time calculation to the first dose of tocilizumab varied between studies [31,32,37] Although our study compared the median days to the first dose of tocilizumab from hospital admission, other studies compared the mean days from hospital admission [30], mean/median days to the first dose from the time of the symptoms [31,32], or mean/median days from the time of diagnosis [32]. However, there were inconsistencies between the results. While some studies indicated a clear delay in drug initiation in nonsurviving vs. surviving patients and the benefits of early administration of the drug [30,32], which in some studies was limited to more severe patients only [32,41], other studies indicated no significant difference in days to the first dose of tocilizumab between survivor and nonsurvivor groups, though deceased patients received it in a shorter period [31]. COVID-19 progression has been described as two phases, a viral phase and a hyperinflammatory phase, and the latter phase is characterized by systemic immune overactivation and cytokine storm that can lead to multiorgan failure. Consequently, early intervention with IL 6 inhibitors may diminish the cytokine storm and improve the outcomes [42]. This benefit of early administration of tocilizumab could also be seen on the days to the first dose of tocilizumab from ICU admission. Although the median time between ICU admission and the first dose of tocilizumab for patients who received the first dose in the ICU was one day for both groups, nonsurvivors had a wider IQR (0–2 vs. 0–3, *p* < 0.05). There was no significant difference in the median days of hospital length of stay after the last dose of tocilizumab between both groups, which was consistent with other studies [31]. However, the median ICU length of stay after the last dose of tocilizumab was significantly longer in the nonsurvivor group (8 vs. 5, *p* < 0.05). Although the median blood ferritin, D-dimer, and LDH concentrations were significantly higher in the nonsurvivor group in our study, there was no significant difference in the median CRP level. These results were inconsistent with other studies that reported no significant differences in these inflammatory markers between groups [31,39]. However, in one of these studies, in which patients were categorized into two groups based on their blood D-dimer concentration (<5000 and >5000 ng/mL), it was found that patients who had blood D-dimer concentration > 5000 ng/mL were more prone to dying from COVID-19 and not responding to tocilizumab treatment [31]. This was consistent with other studies that assessed the association between D-dimer concentration and disease severity [43]. Another study reported no significant differences in the ferritin, D-dimer, or LDH concentrations between groups and a significant difference in the CRP level [30]. Furthermore, Ardanaz et al. [32] reported baseline elevation in these inflammatory biomarkers in the nonsurvivor vs. the survivor group, but no statistical analysis was conducted. Troponin is a marker of myocarditis and cardiac damage. Our study indicated that the blood troponin level was significantly correlated with mortality in COVID-19 patients on tocilizumab. This disappeared in the univariate regression analysis; however, it continued to be available in other studies [32]. Similarly to what was reported by other studies, there was an inverse relationship between platelet count and the mortality of COVID-19 patients on tocilizumab [32,39]. Deceased patients had significantly lower lymphocyte counts than surviving patients, which was consistent with other cohorts that reported the same observation [32,39], one without statistical analysis [32]. Some studies reported a direct relation between blood fibrinogen level and mortality of COVID-19 patients on tocilizumab [32,39]. However, this element was not assessed in our study. While our study showed that the nonsurvivor group had a nonsignificant higher median blood AST level, this increase was significant in another study [39]. Our study reported a significantly higher blood creatinine level in the nonsurvivor group, which was also higher in another study without being significant [39].

Although in the univariate binary logistic regression, 22 baseline variables showed a statistically significant association (*p* < 0.05) with mortality in COVID-19 patients on tocilizumab, ICU admission (**↑**), age of the patient (**↑**), number of tocilizumab doses categorized into four groups (**↑**), days between hospital admission and the first dose of the tocilizumab (**↑**), hospital length of stay (**↓**), D-dimer concentration (**↑**), blood phosphate concentration (**↓**), RDW (**↑**), MCV (**↑**), AST (**↑**), platelets (**↓**), and creatinine (**↑**) were the only variables that remained significant in the multivariable binary logistic regression. In contrast, in another study used Cox stepwise proportional hazard regression models of baseline variables to assess factors that affect time to death. Arandaz et al. reported age of the patient (**↑**), myalgia on admission (**↓**), DM (**↑**), immunosuppression (**↑**), days to tocilizumab administration from COVID-19 diagnosis (**↑**), and blood platelet concentration (**↓**) as the only baseline variables that retained in the final regression model [32]. Furthermore, Lohse et al. reported lymphocytes (**↓**), platelets (**↓**), and blood AST level (**↑**) as predictors of mortality in their final multivariable binary logistic regression model. On the other hand, one study showed only one predictor in the final regression model (tocilizumab within ≤ 12 days of symptoms onset) [30]. All of these were consistent with our study and show the benefit of early administration of tocilizumab in COVID-19 patients.

Analysis of the short-term effects of tocilizumab on COVID-19 patients revealed significant increases in blood calcium, phosphate, carbon dioxide, BUN, ALT, RDW, platelets, lymphocyte, monocyte, and eosinophile absolute at the time of discharge. However, all were still within the normal range except BUN and a significant decrease in the blood AST to the normal ranges. Furthermore, although there was a significant decrease in blood CRP and LDH level at discharge, the values were still higher than the normal upper ranges. The same effects were noticed in patients who were discharged alive, with BUN remaining the normal range and no change in the RDW. Furthermore, although patients who were discharged alive showed significant increases in blood hemoglobin level and percentage of hematocrits and a significant decrease in mean platelet volume and neutrophile absolute, all were within normal ranges. All of these data indicated the safety of a single dose of tocilizumab in COVID-19 patients except the slight increase in the blood BUN level, which could not be linked to tocilizumab administration with the presence of normal blood creatinine level. Furthermore, although there was a significant decrease in the blood CRP and LDH levels, these levels were still higher than the normal upper ranges, indicating the long-term effect of the inflammatory condition induced by SARS-CoV-2 infection. Morrison et al. assessed the short-term effects of tocilizumab on survived vs. nonsurvived patients (patients received two doses at maximum). They noticed a reduction in the CRP level and serum ferritin concentration in both groups 15 days after the first dose and decreases in LDH and D-dimer concentrations in the survived patients only, with these increasing in the deceased patients. However, no statistical analysis was conducted to see the significance of the changes from the baseline, and the study was limited by its small sample size (*n* = 27) [30]. Similarly, Arandaz et al. [32] assessed the effect of tocilizumab after 3, 6, and 9 days of administration on survived vs. deceased patients (*n* = 120, one dose = 90, two doses = 20, and three doses = 2), and based on the presented graphs (no values with no statistical analysis in comparison to the baseline), after 9 days of tocilizumab administration, there were decreases in IL-6, CRP, and LDH levels and almost no change in neutrophile and platelets counts in the survived patients. In contrast, deceased patients showed increased IL-6, CRP, LDH, and neutrophil and platelet counts on the ninth day of tocilizumab administration. However, both groups showed increases in blood troponin and lymphocyte count. Furthermore, Conrozier et al. [44] assessed the short-term effect of two doses of tocilizumab (8 mg/kg) at the time of discharge (which varied) and noticed that there were significant decreases in blood CRP, ferritin, and fibrinogen concentrations and significant increases in lymphocyte and ALT concentration in reference to day zero. There were no significant changes in blood D-dimer, leucocyte, hemoglobin, platelet, or creatinine levels. However, the study had many limitations, including in deceased patients; the changes in biomarkers were assessed and analyzed on day 4 only because four patients were still alive after day 6, and overall, the sample size was too small, with the availability of a limited number of patients on days 4, 6, and 8 (total *n* = 40). Our study was unique because it was the first study that assessed the clinical effectiveness of different doses of tocilizumab and the short-term effects on the different laboratory biomarkers. Although patients who received more doses of tocilizumab had a higher percentage of mortality, this cannot be referred solely to the drug itself, as patients who received more doses of tocilizumab were significantly older, and the majority of them were ICU patients. However, receiving more doses of tocilizumab had no effect on reducing hospital length of stay, ICU length of stay, hospital length of stay after the last dose of tocilizumab, or time to death, which, unfortunately, were all higher in patients who received more doses of tocilizumab. Trend analysis of the short-term effects of different doses of tocilizumab in COVID-19 patients revealed mainly a hematological effect. Tocilizumab had the ability to significantly increase hemoglobin, carbon dioxide, MCV, and RDW levels and decrease blood D-dimer, AST, and CRP concentrations, which is different from what was reported by other studies that assessed the effect of tocilizumab in rheumatoid arthritis patients and reported an increase in blood hemoglobin level [45,46]. Although tocilizumab had the effects of increasing mean platelet volume and platelet distribution width, other studies in rheumatoid arthritis indicated its potential to induce thrombocytopenia [47]

Infections after tocilizumab administration have been well reported in the literature. Our research indicated a doubling in the number of patients who developed infections and a fourfold increase in the incidence of infections after tocilizumab administration, as 21.3% of our patients developed an infection after tocilizumab administration, which was comparable to other studies that reported up to 32% of patients developing infection after tocilizumab administration. However, these percentages have varied wildly between cohorts [31,32,37]. In our study, 51.3% of the secondary infections after tocilizumab administration were attributed to a fungus, and the development of infection was faster, with median days to the first incidence of infection after tocilizumab administration being 6 days compared with the 9 days reported by another study [30]. The increased number of infections can be surely linked with the number of tocilizumab doses received by the patients, based on the significant moderate Spearman correlation coefficient and the nonlinear kinetic of tocilizumab with a dose-dependent half-life (6.3 days after a single dose of 10 mg/kg and 11 days after 3 doses of 8 mg/kg) [48].

It is worth mentioning that our study had some limitations, which included (i) the retrospective nature of the study, with the absence of a control group having the same disease severity with no tocilizumab administration; (ii) the study representing a one-center/one-country cohort; (iii) the study results possibly being confounded by bias, including differences in the baseline characteristics, comorbidities, and length of stay; and (iv) a lack of statistical power due to missing values for some laboratory results. On the other hand, the strengths of our study included (i) it being the largest study nationally and the second largest internationally that assessed the effect of tocilizumab in COVID-19 patients; (ii) being the largest study ever in terms of the assessed parameters in COVID-19 patients on tocilizumab; (iii) being the first study showing the best way to express the doses of tocilizumab administered to patients in clinical trials; (iv) being the first study assessing the clinical effectiveness of multiple doses (more than two) of tocilizumab in COVID-19 patients; (v) being the first study assessing the short-term effects of different doses of tocilizumab in COVID-19 patients; (vi) a unique statistical approach that allowed different variables to be analyzed and various models of variables significantly contributing to mortality to be formed.

## 5. Conclusions

The findings of this study are important to guiding the use of tocilizumab in COVID-19 patients. It showed the benefit of early administration of tocilizumab once administered to patients with low D-dimer and phosphate concentrations. Moreover, there was a trend towards higher mortality in patients with COVID-19 with increasing numbers of doses of tocilizumab administered, with no benefit of administering more than one dose. Furthermore, infections post-tocilizumab administration correlated significantly to the number of tocilizumab doses and could be considered a life-threatening consequence of tocilizumab. While randomized controlled clinical trials are needed to demonstrate the clinical effectiveness and adverse effect of different doses of tocilizumab in COVID-19 patients, more research is required to clarify any possible long-term adverse effects of the drug.

## Figures and Tables

**Table 1:** Univariate and multivariable logistic parameters (demographical, clinical characteristics, concurrent drug administration, time- and tocilizumab-related parameters, and lab results) for mortality in COVID-19 patients on tocilizumab.

Univariate Regression Parameter with *p* < 0.1		Multivariable Regression Parameter (Yes/No)	Reference Group
Demographical Characteristics			
Age (year)		Yes	
Body mass index category	Underweight (<18.5)	Yes	Normal weight
	Overweight (25.0–29.9)	Yes	
	Obese (>35.0)	Yes	
Clinical Characteristics			
Number of comorbidities	1	Yes	No comorbidity
	2	Yes	
	3 or more	Yes	
Concurrent Drug Administration			
Patients received at least one type of antiviral drugs		Yes	Patients did not receive antiviral drugs
Patient received hydroxychloroquine		Yes	Patients did not receive hydroxychloroquine
Time-Related Parameters			
Hospital-Related Parameters			
Hospital length of stay (d)		Yes	
ICU-Related Parameters			
ICU Admission	Admitted to ICU	Yes	Patients not admitted to ICU
For patients admitted to ICU: time between hospital admission and ICU admission (d)		No ^a^	
For All patients, ICU Length of Stay (d)		No ^b^	
For patients admitted to ICU, ICU Length of Stay (d)		No ^b^	
Tocilizumab Related Parameters			
Number of Tocilizumab Doses			
Number of tocilizumab doses (*n*)		Yes	
Number of tocilizumab doses	Two doses (*n*, %)	Yes	Patients received one dose
	Three doses (*n*, %)	Yes	
	Four or more doses (*n*, %)	Yes	
Total Tocilizumab (mg)			
(Total tocilizumab)^2^ (mg)		Yes	
Total tocilizumab (mg)	801–1600 (*n*, %)	Yes	Patients received <800 mg tocilizumab
	1601–2400 (*n*, %)	Yes	
	>2401 (*n*, %)	Yes	
Total Tocilizumab (mg/Kg)			
Total tocilizumab (mg/kg)		Yes	
Total tocilizumab (mg/Kg)	10.1–21 mg/kg	Yes	Patients received <10 mg/kg tocilizumab
	21.1–32 mg/kg	Yes	
	>32 mg/k	Yes	
Time-Related Parameters (All Patients)			
The time between admission and the first dose of tocilizumab (d)		Yes	
ICU length of stay after the last dose of tocilizumab (d)		No ^b^	
Laboratory Characteristics			
Electrolytes			
Potassium (3.5–5.1 mEq/L)		Yes	
Phosphorus (2.5–4.5 mg/dL)		Yes	
Chemical Profile			
Blood urea nitrogen (6–20 mg/dL)		No ^a^	
Creatinine (0.66–1.25 mg/dL)		Yes	
Hepatic Profile			
Bilirubin, total (0.2–1.3 mg/dL)		Yes	
AST (SGOT) (17–59 IU/L)		Yes	
LDH (120–246 IU/L)		Yes	
Complete Blood Count			
RBC (4.5–5.9 M/µL)		Yes	
Hematocrit (41–53%)		No ^d^	
MCV (77–96 fL)		Yes	
MCH (26–34 pg)		No ^d^	
RDW (10.9–15.7)		Yes	
Platelets (150–450 K/µL)		Yes	
Lymph. absolute (0.9–4.9 K/µL)		Yes	
Coagulation			
APTT (22–33 Secs)		Yes	
D-dimer quantitative (0.0–0.7 mg/L FEU)		Yes	

^a^ removed because of insufficient sample size (it was considered a noninclusive factor, as not all of the patients were admitted to the ICU); ^b^ removed because of multicollinearity and small sample size; ^d^: removed because of multicollinearity with other laboratory results.

**Table 2:** Clinical and laboratory characteristics of COVID-19 patients who received single doses of tocilizumab—the most recent lab results before tocilizumab administration vs. the most recent lab results before discharge (all patients *n* = 146).

Parameters				Value		
Number of patients (*n*, %)				146 (48.7)		
Survival						
Survivors (*n*, %)				133 (91.1%)		
Nonsurvivors (*n*, %)				13 (8.9%)		
Tocilizumab-Related Parameters						
Total tocilizumab (mg) median (IQR)				400 (400.00–600.00)		
Total tocilizumab (mg/kg) (mean ± SD)				5.88 (5.00–7.00)		
Demographic Characteristics						
Age median (IQR)				58.50 (53.00–69.00)		
Gender						
Male (*n*, %)				93 (63.7%)		
Female (*n*, %)				53 (36.3%)		
Nationality						
Saudi (*n*, %)				133 (91.7%)		
Non-Saudi (*n*, %)				12 (8.3%)		
Weight-Related Parameters						
Weight median (IQR)				81.25 (73.85–96.00)		
Body mass index median (IQR)				31.23 (27.13–35.15)		
Body Mass Index Category						
Underweight (<18.5) (*n*, %)				1 (0.7%)		
Normal weight (18.5–24.9) (*n*, %)				12 (8.2%)		
Overweight (25.0–29.9) (*n*, %)				48 (32.9%)		
Obese (>35.0) (*n*, %)				85 (58.2%)		
Smoking Status						
Nonsmoker *(n*, %)				111 (76.0%)		
Former smoker (*n*, %)				22 (15.1%)		
Active smoker (*n*, %)				13 (8.9%)		
Clinical Characteristics						
Number of Comorbidities						
0 (*n*, %)				39 (26.7%)		
1 (*n*, %)				35 (24.0%)		
2 (*n*, %)				34 (23.3%)		
3 or more (*n*, %)				38 (26.0%)		
Concurrent Drug Administration						
Patients received at least one type of antiviral drugs (*n*, %)				22 (15.1%)		
Patient received at least one type of corticosteroids (*n*, %)				144 (98.6%)		
Patient received hydroxychloroquine (*n*, %)				34 (23.3%)		
Time-Related Parameters						
Hospital-Related Parameters						
Hospital length of stay (median (IQR))				11.00 (7.00–16.00)		
ICU-Related Parameters						
Not admitted to ICU (*n*, %)				99 (67.8%)		
Admitted to ICU (*n*, %)				47 (32.2%)		
For patients admitted to ICU: days between hospital admission and ICU admission (median (IQR))				4.00 (1.00–5.00)		
ICU length of stay (median (IQR))				8.00 (4.00–12.00)		
Time-Related Parameters—Tocilizumab (All Patients)						
The time between admission and the first dose of tocilizumab (median (IQR))				5.00 (3.00–9.25)		
For patients who received the first dose of tocilizumab in ICU: Time between ICU admission and first dose (median (IQR))				1.50 (0.00–4.00)		
ICU length of stay after the last dose of tocilizumab (median (IQR))				6.50 (3.00–10.00)		
Hospital length of stay after the last dose of tocilizumab (median (IQR))				10.00 (6.75–15.00)		
Laboratory Characteristics						
Parameter		Most recent lab results before TCZ administration	Most recent lab results before discharge/decease		*n*	*p*-value
Electrolytes						
Sodium (135–145 mEq/L) (median (IQR))		136.00 (134.00–138.00)	135.00 (133.00–137.75)		140	0.470703
Potassium (3.5–5.1 mEq/L) (median (IQR))		4.30 (4.00–4.70)	4.30 (4.00–4.70)		140	0.365723
Calcium (8.6–10.0 mg/dL) (median (IQR))		8.50 (8.10–8.85	8.65 (8.30–9.13)↑		41	0.007505 ***
Magnesium (1.7–2.4 mg/dL) (median (IQR))		2.10 (1.90–2.20)	2.10 (2.00–2.20		51	0.409051
Phosphorus (2.5–4.5 mg/dL) (median (IQR))		3.40 (3.00–3.90)	4.00 (3.40–4.50)↑		37	0.015499 *
Chloride (98–107 mEq/L) (median (IQR))		101.00 (98.00–104.00)	101.00 (98.00–103.00)		140	0.588506
Carbon dioxide (22–28 mEq/L) (median (IQR))		25.00 (22.00–27.00)	26.00 (23.00–29.00)↑		140	0.000453 ***
Chemical Profile						
Blood urea nitrogen (6–20 mg/dL) (median (IQR))		17.50 (13.00–25.25)	21.00 (16.25–27.75)↑		140	0.000001 ***
Creatinine (0.66–1.25 mg/dL) (median (IQR))		0.80 (0.60–0.93)	0.70 (0.60–0.90)		140	0.848083
Blood glucose level (73–178 mg/dL) (mean ± SD)		214.63 ± 188.21	186.38 ± 124.27		8	0.275070
Lactic acid (0.7–2.1 mmol/L) (median (IQR))		1.50 (1.20–2.00)	1.45 (1.10–2.05)		39	0.074353
Cardiac Profile						
High-sensitivity troponin (0.000–0.026 ng/mL) (median (IQR))		0.0070 (0.0040–0.0180)	0.0085 (0.0030–0.0438)		16	0.179565
B-type natriuretic peptide (<100 pg/mL) (median (IQR))		49.25 (19.08–118.40)	53.10 (12.10–134.00)		16	0.454498
Hepatic Profile						
Bilirubin, total (0.2–1.3 mg/dL) (median (IQR))		0.5000 (0.4000–0.7000)	0.5000 (0.3000–0.7000)		99	0.097626
Alk. phosphatase (53–128 IU/L) (median (IQR))		69.00 (56.00–89.00)	68.00 (56.00–83.00)		99	0.107822
ALT (SGPT) (<50 IU/L) (median (IQR))		32.00 (22.00–49.50)	46.00 (28.00–71.00)↑		99	0.038088 *
AST (SGOT) (17–59 IU/L) (median (IQR))		43.00 (34.50–59.00)	41.00 (30.00–57.00)↓		99	0.018904 *
LDH (120–246 IU/L) (median (IQR))		325.00 (262.00–415.25)	304.00 (230.25–388.75)↓		99	0.044423 *
Iron/Anemia Profile						
Ferritin (21.81–274.66 ng/mL) (median (IQR))		735.00 (406.75–1415.78)	744.00 (372.00–1358.00)		41	0.635256
CRP, Quantitative						
CRP, quantitative (<1.0 mg/dL) (median (IQR))		13.90 (5.75–20.00)	1.40 (0.60–2.40)↓		134	1.0851 × 10^−29^ ***
Complete Blood Count						
WBC (4.0–10.0 K/µL) (median (IQR))		7.40 (5.40–10.13)	7.90 (5.60–10.20)		143	0.738005
RBC (4.5–5.9 M/µL) (median (IQR))		4.74 (4.25–5.23)	4.76 (4.32–5.21)		143	0.352542
Hemoglobin (13.5–17.5 g/dL) (median (IQR))		12.30 (11.10–13.63)	12.50 (11.10–13.60)		143	0.301700
Hematocrit (41–53%) (median (IQR))		38.10 (34.75–41.88)	39.00 (35.20–41.90)		143	0.208113
MCV (77–96 fL) (median (IQR))		82.60 (73.88–87.70)	82.90 (75.50–88.40)		143	0.110836
MCH (26–34 pg) (median (IQR))		26.90 (22.98–29.10)	27.00 (23.20–29.20)		143	0.477775
MCHC (32–36 g/dL) (median (IQR))		32.35 (31.17–33.30)	32.10 (31.10–33.50)		143	0.267191
RDW (10.9–15.7) (median (IQR))		13.80 (12.78–15.33)	13.80 (12.90–16.30)↑		143	0.046934 *
Platelets (150–450 K/µL) (median (IQR))		245.00 (196.75–321.00)	307.00 (226.00–402.00)↑		143	1.3391 × 10^−7^ ***
Mean platelet volume (9.0–13.0 fL) (median (IQR))		10.60 (9.73–11.38)	10.40 (9.80–11.10)		125	0.104609
Platelet distribution width (10.1–16.1 fL) (median (IQR))		12.30 (11.10–14.43)	12.30 (10.70–13.90)		125	0.619817
Neut. absolute (1.8–7.0 K/µL) (median (IQR))		5.95 (3.90–8.27)	5.35 (3.10–7.73)		138	0.264962
Lymph. absolute (0.9–4.9 K/µL) (median (IQR))		0.8000 (0.6000–1.2000)	1.2000 (0.9000–2.0000)↑		138	3.7661 × 10^−12^ ***
Mono. absolute (0.0–1.0 K/µL) (median (IQR))		0.3000 (0.2000–0.5000)	0.5500 (0.4000–0.8000)↑		138	5.0448 × 10^−12^ ***
Eos. absolute (0.0–0.4 K/µL) (median (IQR))		0.0000 (0.0000–0.0000)	0.0000 (0.0000–0.1000)↑		137	1.4717 × 10^−9^ ***
Baso. absolute (0.0–0.1 K/µL) (median (IQR))		0.0000 (0.0000–0.0000)	0.0000 (0.0000–0.0000)		137	0.307456
Band neutrophil absolute (0.0–0.5 K/µL) (median (IQR))		0.3050 (0.1225–0.6050)	0.2300 (0.1400–0.5600)		27	0.556298
Coagulation						
Prothrombin time (9.8–12.7 s) (median (IQR))		11.40 (10.72–11.90)	11.85 (11.48–13.03)		24	0.133801
I.N. ratio (1.0–1.2) (median (IQR))		1.00 (1.00–1.10)	1.10 (1.00–1.20)		24	0.109375
APTT (22–33 s) (median (IQR))		29.50 (27.25–34.50)	27.00 (24.00–29.50)		19	0.063568
D-dimer quantitative (0.0–0.7 mg/L FEU) (median (IQR))		0.66 (0.37–1.33)	0.87 (0.42–1.62)		115	0.852052
Urine Analysis						
Urine specific gravity (1.000–1.030) (mean ± SD)		1.0223 ± 0.01092	1.0199 ± 0.00839		12	0.538078
Urine pH (4.5–8.0)	5 (*n*, %)				12	0.857462
	5.5 (*n*, %)					
	6 (*n*, %)					
	6.5 (*n*, %)					
	7 (*n*, %)					
	7.5 (*n*, %)					
Urine protein (negative) (*n*)	Negative	2	5		12	0.250000
	Available	10	7			
Urine glucose (negative) (*n*)	Negative	9	9		12	1.000000
	Available	3	3			
Urine ketone (negative) (*n*)	Negative	7	9		12	0.625000
	Available	5	3			
Urine blood (negative) (*n*)	Negative				12	0.781511
	Trace					
	Moderate					
	Numerous					
Urine bilirubin (negative) (*n*)	Negative	12	12		12	1.000000
	Available	0	0			
Urine urobilinogen (0.0–1.0 EU/dL) (*n*)	Normal	10	12		12	0.500000
	Abnormal	2	0			
Urine nitrites (negative) (*n*)	Negative	12	12		12	1.000000
	Available	0	0			
Urine leuk. esterase (Negative) (*n*)	Negative	10	8		12	0.625000
	Available	2	4			

* *p* < 0.05 is statistically significant; ** *p* < 0.01 is statistically very significant; *** *p* < 0.001 is statistically extremely significant.

**Table 3:** Clinical outcomes for COVID-19 patients who received different doses of tocilizumab, categorized based on the number of doses administered to the patient.

Parameters		Total Patients	One Dose	Two Doses	Three Doses	Four or More	*p*-Value
Number of patients (*n*, %)		300 (100%)	146 (48.7%)	82 (27.3%)	36 (12.0%)	36 (12.0%)	
Survival							
Survivors (*n*, %)		237 (79%)	133 (91.1%)	57 (69.5%)	29 (80.6%)	18 (50.0%)	8.9772 × 10^−8^ ***
Nonsurvivors (*n*, %)		63 (21%)	13 (8.9%)	25 (30.5%)	7 (19.4%)	18 (50.0%)	
Tocilizumab-Related Parameters							
Total Tocilizumab (mg)							
Total tocilizumab (mg) (median (IQR))		800.00 (400.00–1600.00)	400.00 (400.00–600.00)	1200.00 (800.00–1400.00)	1800.00 (1450.00–2000.00)	3200.00 (2600.00–4779.75)	4.2386 × 10^−53^ ***
Total Tocilizumab (mg/Kg)							
Total tocilizumab (mg/kg) (mean ± SD)		14.82 ± 13.80	6.32 ± 1.88	13.46 ± 3.64	21.50 ± 4.02	45.74 ± 15.20	6.6742 × 10^−43^ ***
Time-Related Parameters							
Hospital-Related Parameters							
Hospital length of stay (median (IQR))		14.00 (9.00–21.00)	11.00 (7.00–16.00)	16.00 (12.00–22.25)	15.00 (12.25–23.00)	31.00 (19.00–51.50)	1.8892 × 10^−18^ ***
ICU-Related Parameters							
ICU admission	Not admitted to ICU (*n*, %)	139 (46.3%)	99 (67.8%)	35 (42.7%)	5 (13.9%)	0 (0.0%)	6.4282 × 10^−16^ ***
	Admitted to ICU (*n*, %)	161 (53.7%)	47 (32.2%)	47 (57.3%)	31 (86.1%)	36 (100.0%)	
For patients admitted to ICU: days between hospital admission and ICU admission (median (IQR))		3.00 (1.00–5.00)	4.00 (1.00–5.00)	3.00 (1.00–7.00)	3.00 (.00–5.00)	3.00 (1.25–6.75)	0.627276
ICU length of stay (median (IQR))		3.00 (0.00–12.00)	0.00 (0.00–4.00)	3.50 (0.00–14.00)	7.50 (4.00–15.00)	18.00 (12.25–34.50)	1.968 × 10^−21^ ***
Time-Related Parameters—Tocilizumab (All Patients)							
Time between admission and first dose of tocilizumab (median (IQR))		3.00 (1.00–5.00)	3.00 (1.00–5.00)	3.00 (1.00–5.00)	2.00 (1.00–3.00)	2.00 (1.00–3.75)	0.176378
For patients received the first dose of tocilizumab in ICU: time between ICU admission and the first dose (median (IQR))		1.00 (0.00–2.00)	1.00 (0.00–4.00)	1.00 (0.00–2.00)	0.00 (0.00–1.00)	1.00 (0.00–2.00)	0.151333
ICU length of stay after last dose of tocilizumab (median (IQR))		7.00 (4.00–13.00)	6.00 (3.00–9.75)	6.50 (4.00–13.00)	5.50 (3.00–12.25)	9.50 (4.00–18.75)	0.059543
Hospital length of stay after last dose of (tocilizumab median (IQR))		8.00 (5.00–13.00)	7.00 (5.00–11.00)	8.50 (6.00–14.00)	8.50 (6.00–14.00)	17.00 (7.50–31.75)	0.000089 ***

*** *p* < 0.001 is statistically extremely significant.

**Table 4:** Short-term effects of different doses of tocilizumab on the laboratory results of COVID-19 (all patients, *n* = 300).

Parameters		*n*	Total Patients	One Dose^i^	Two Doses^ii^	Three Doses^iii^	Four or More^iv^	*p*-Value
Number of patients (*n*, %)		300	300 (100%)	146 (48.7%)	82 (27.3%)	36 (12.0%)	36 (12.0%)	
Survival								
Survivors (*n*, %)		237	237 (79%)	133 (91.1%)	57 (69.5%)	29 (80.6%)	18 (50.0%)	8.9772 × 10^−8^ ***
Nonsurvivors (*n*, %)		63	63 (21%)	13 (8.9%)	25 (30.5%)	7 (19.4%)	18 (50.0%)	
Laboratory Characteristics								
Electrolytes								
Sodium (135–145 mEq/L) (median (IQR))		292	135.00 (133.00–138.00)	135.00 (133.00–137.75)	135.00 (134.00–138.00)	134.00 (132.00–136.00)	136.00 (133.00–140.50)	0.139298
Potassium (3.5–5.1 mEq/L) (median (IQR))		292	4.30 (4.00–4.70)	4.30 (4.00–4.70)	4.40 (4.00–4.80)	4.35 (3.90–4.70)	4.25 (3.93–4.88)	0.900512
Calcium (8.6–10.0 mg/dL) (median (IQR))		174	8.65 (8.20–9.10)	8.65(8.30–9.13)	8.60 (8.20–9.10)	8.65 (8.05–9.00)	8.70 (8.20–9.15)	0.932224
Magnesium (1.7–2.4 mg/dL) (median (IQR))		210	2.10 (1.90–2.32)	2.10 (2.00–2.20)	2.20 (2.00–2.40)	2.20 (1.92–2.50)	1.95 (1.80–2.58)	0.050987
Phosphorus (2.5–4.5 mg/dL) (median (IQR))		175	4.10 (3.50–4.80)	4.00 (3.40–4.50)	4.15 (3.60–4.93)	4.40 (4.00–4.90)	4.10 (3.40–5.40)	0.165237
Chloride (98–107 mEq/L) (median (IQR))		292	101.00 (98.00–104.00)	101.00 (98.00–103.00)	101.50 (98.00–105.00)	100.00 (96.25–103.00)	100.50 (97.00–103.00)	0.244027
Carbon Dioxide (22–28 mEq/L) (median (IQR))		292	27.00 (23.00–29.00)	26.00 (23.00–29.00)	27.00 (23.00–29.00)	28.00 (24.25–31.75)	28.00 (24.25–31.00)	0.186607
Chemical Profile								
Blood urea nitrogen (6–20 mg/dL) (median (IQR))		292	23.00 (17.00–37.00)	21.00 (16.25–27.75)	26.50 (20.00–42.50)	24.00 (20.00–35.25)	39.00 (14.25–71.25)	0.000244 ***
Creatinine (0.66–1.25 mg/dL) (median (IQR))		292	0.8000 (0.6000–1.1000)	0.7000 (0.6000–0.9000)	0.9000 (*0*.6000–1.3000)	0.8000 (0.7000–1.0750)	0.7500 (0.5000–2.4500)	0.023748 *
Blood glucose level (73–178 mg/dL) (median (IQR))		22	168.00 (109.75–240.25)	171.00 (109.00–234.00)	235.00 (147.25–301.75)	67.00 (8.80–)	156.00 (141.00–274.50)	0.321462
Lactic acid (0.7–2.1 mmol/L) (median (IQR))		142	1.80 (1.30–2.43)	1.45 (1.10–2.05)	1.80 (1.20–2.95)	1.80 (1.50–2.20)	2.00 (1.40–3.05)	0.031889 *
Cardiac Profile								
High-sensitivity troponin (0.000–0.026 ng/mL) (median (IQR))		75	0.0200 (0.0040–0.1570)	0.0085 (0.0030–0.0438)	0.0330 (0.0040–0.3800)	0.1100 (0.0075–0.3270)	0.0310 (0.0110–0.1385)	0.089200
B-type natriuretic peptide (<100 pg/mL) (median (IQR))		90	55.75 (17.08–189.25)	53.10 (12.10–134.00)	51.10 (12.45–180.35)	33.40 (14.68–146.88)	83.45 (34.08–267.48)	0.382850
Hepatic Profile								
Bilirubin, total (0.2–1.3 mg/dL) (median (IQR))		232	0.6000 (0.4000–0.8000)	0.5000 (0.3000–0.7000)	0.6000 (0.4000–0.8500)	0.7000 (0.4000–1.1000)	0.6500 (0.4250–1.7750)	0.002946 ***
Alk. phosphatase (53–128 IU/L) (median (IQR))		231	70.00 (56.00–90.00)	68.00 (56.00–83.00)	69.50 (58.00–90.00)	60.50 (48.00–81.25)	80.50 (59.75–113.00)	0.040667 *
ALT (SGPT) (<50 IU/L) (median (IQR))		231	46.00 (28.00–89.00)	46.00 (28.00–71.00)	46.50 (27.50–91.75)	50.00 (34.25–103.25)	41.50 (22.75–92.75)	0.764289
AST (SGOT) (17–59 IU/L) (median (IQR))		231	39.00 (28.00–55.00)	41.00 (30.00–57.00)	39.00 (28.75–52.50)	35.50 (27.25–50.00)	41.00 (23.25–76.75)	0.742672
LDH (120–246 IU/L) (median (IQR))		232	321.50 (249.50–465.50)	304.00 (230.25–388.75)	346.50 (280.00–473.50)	276.00 (239.50–487.50)	463.00 (304.00–681.00)	0.002791 ***
Iron/Anemia Profile								
Ferritin (21.81–274.66 ng/mL) (median (IQR))		138	691.50 (350.00–1201.50)	744.00 (372.00–1358.00)	602.00 (450.00–1200.00)	604.50 (243.50–1301.00)	550.00 (324.50–964.00)	0.809493
CRP, Quantitative								
CRP, quantitative (<1.0 mg/dL) (median (IQR))		284	0.90 (0.49–2.00)	1.40 (0.60–2.40)	0.85 (0.49–1.50)	0.49 (0.49–1.60)	0.50 (0.49–1.68)	0.002306 ***
Complete Blood Count								
WBC (4.0–10.0 K/µL) (median (IQR))		295	8.20 (5.60–11.10)	7.90 (5.60–10.20)	8.95 (6.25–12.30)	8.65 (6.00–12.17)	8.80 (4.85–16.98)	0.204886
RBC (4.5–5.9 M/µL) (median (IQR))		295	4.58 (3.95–5.13)	4.76 (4.32–5.21)	4.40 (3.75–5.06)	4.53 (3.91–5.17)	3.82 (3.11–4.47)	0.000003 ***
Hemoglobin (13.5–17.5 g/dL) (mean ± SD)		295	12.11 ± 2.21	12.41 ± 1.95	12.08 ± 2.28	11.99 ± 2.36	11.08 ± 2.58	0.012874 *
Hematocrit (41–53%) (mean ± SD)		295	37.76 ± 6.27	38.58 ± 5.40	37.67 ± 6.52	37.55 ± 6.73	34.93 ± 7.73	0.061542
MCV (77–96 fL) (mean ± SD)		295	84.73 ± 9.29	81.67 ± 8.80	86.86 ± 8.89	85.85 ± 6.85	91.09 ± 9.65	1.422 × 10^−8^ ***
MCH (26–34 pg) (median (IQR))		295	27.80 (25.10–29.50)	27.00 (23.20–29.20)	28.30 (25.88–29.60)	27.55 (25.60–29.05)	29.35 (27.05–30.95)	0.000625 ***
MCHC (32–36 g/dL) (mean ± SD)		295	31.99 ± 1.71	32.11 ± 1.85	31.97 ± 1.40	31.85 ± 1.28	31.69 ± 2.12	0.618878
RDW (10.9–15.7) (median (IQR))		295	14.50 (13.20–16.90)	13.80 (12.90–16.30)	15.00 (13.23–17.20)	14.55 (13.20–17.08)	16.30 (14.93–18.65)	0.000133 ***
Platelets (150–450 K/µL) (mean ± SD)		295	276.22 ± 128.33	315.50 ± 116.52	251.81 ± 116.35	282.33 ± 143.95	168.33 ± 110.89	1.0606 × 10^−9^ ***
Mean platelet volume (9.0–13.0 fL) (median (IQR))		272	10.60 (10.00–11.50)	10.40 (9.80–11.10)	10.70 (10.10–11.60)	10.50 (9.80–12.00)	11.90 (10.40–12.75)	0.000102 ***
Platelet distribution width (10.1–16.1 fL) (median (IQR))		272	12.60 (11.00–15.00)	12.30 (10.70–13.90)	12.70 (11.40–15.10)	12.40 (10.85–15.85)	15.00 (12.50–18.40)	0.001880 **
Neut. absolute (1.8–7.0 K/µL) (median (IQR))		290	5.60 (3.10–8.63)	5.35 (3.10–7.73)	6.20 (3.55–9.83)	6.45 (3.68–9.25)	5.35 (2.25–14.53)	0.437818
Lymph. absolute (0.9–4.9 K/µL) (median (IQR))		290	1.3000 (0.8000–2.0000)	1.2000 (0.9000–2.0000)	1.3000 (0.7250–1.9000)	1.8000 (0.9000–2.6500)	1.3000 (0.7000–2.2500)	0.154881
Mono. absolute (0.0–1.0 K/µL) (median (IQR))		290	0.6000 (0.4000–0.8000)	0.5500 (0.4000–0.8000)	0.6000 (0.4000–0.8000)	0.6000 (0.4000–0.8000)	0.5000 (0.2000–0.7000)	0.347368
Eos. absolute (0.0–0.4 K/µL) (median (IQR))		289	0.0000 (0.0000–0.2000)	0.0000 (0.0000–0.1000)	0.1000 (0.0000–0.2000)	0.0000 (0.0000–0.2000)	0.0000 (0.0000–0.1000)	0.117557
Baso. absolute (0.0–0.1 K/µL) (median (IQR))		289	0.0000 (0.0000–0.0000)	0.0000 (0.0000–0.0000)	0.0000 (0.0000–0.0000)	0.0000 (0.0000–0.0000)	0.0000 (0.0000–0.0000)	0.163588
Band neutrophil absolute (0.0–0.5 K/µL) (median (IQR))		123	0.4000 (0.1700–0.8600)	0.2300 (0.1400–0.5600)	0.4300 (0.2025–1.0525)	0.8600 (0.4950–1.7250)	0.6900 (0.1400–1.3300)	0.012533 *
Coagulation								
Prothrombin time (9.8–12.7 Secs) (median (IQR))		133	12.10 (11.40–13.20)	11.85 (11.48–13.03)	12.10 (11.25–13.13)	12.20 (10.90–12.90)	12.25 (11.60–13.70)	0.479953
I.N. ratio (1.0–1.2) (median (IQR))		133	1.10 (1.00–1.20)	1.10 (1.00–1.20)	1.10 (1.00–1.20)	1.10 (1.00–1.20)	1.10 (1.10–1.30)	0.277785
APTT (22–33 s) (median (IQR))		128	26.00 (24.00–29.75)	27.00 (24.00–29.50)	27.00 (25.00–30.00)	26.00 (24.00–32.25)	26.00 (23.75–29.75)	0.797481
D-dimer quantitative (0.0–0.7 mg/L FEU) (median (IQR))		245	1.07 (0.48–2.35)	0.87 (0.42–1.62)	1.22 (0.55–3.21)	1.39 (0.50–4.29)	1.51 (0.87–3.97)	0.004335 ***
Urine Analysis								
Urine specific gravity (1.000–1.030) (mean ± SD)		49	1.0195 ± 0.00914	1.0222 ± 0.00955	1.0199 ± 0.00881	1.0126 ± 0.00508	1.0149 ± 0.00720	0.063610
Urine pH (4.5–8.0)	5 (*n*, %)	49	15 (5.0%)	7 (29.2%)	5 (41.7%)	2 (40.0%)	1 (12.5%)	0.636921
	5.5 (*n*, %)		8 (2.7%)	5 (20.8%)	1 (8.3%)	0 (0.0%)	2 (25.0%)	
	6 (*n*, %)		8 (2.7%)	5 (20.8%)	2 (16.7%)	0 (0.0%)	1 (12.5%)	
	6.5 (*n*, %)		12 (16.7%)	4 (33.3%)	4 (20.0%)	1 (37.5%)	3 (%)	
	7 (*n*, %)		6 (2.0%)	3 (12.5%)	0 (0.0%)	2 (40.0%)	1 (12.5%)	
	7.5 (*n*, %)		0 (0.0%)	0 (0.0%)	0 (0.0%)	0 (0.0%)	0 (0.0%)	
Urine protein (negative) (*n*, %)	Negative	49	27 (9.0%)	15 (62.5%)	6 (50.0%)	2 (40.0%)	4 (50.0%)	0.760679
	Available		22 (7.3%)	9 (37.5%)	6 (50.0%)	3 (60.0%)	4 (50.0%)	
Urine glucose (negative) (*n*, %)	Negative	49	37 (12.3%)	17 (70.8%)	9 (75.0%)	4 (80.0%)	7 (87.5%)	0.920407
	Available		12 (4.0%)	7 (29.2%)	3 (25.0%)	1 (20.0%)	1 (12.5%)	
Urine ketone (negative) (*n*, %)	Negative	49	38 (12.7%)	18 (75.0%)	10 (83.3%)	5 (100.0%)	5 (62.5%)	0.489538
	Available		11 (3.7%)	6 (25.0%)	2 (16.7%)	0 (0.0%)	3 (37.5%)	
Urine blood (negative) (*n*, %)	Negative	49	17 (5.7%)	8 (33.3%)	5 (41.7%)	1 (20.0%)	3 (37.5%)	0.676392
	Trace		8 (2.7%)	6 (25.0%)	1 (8.3%)	1 (20.0%)	0 (0.0%)	
	Moderate		13 (4.3%)	7 (29.2%)	2 (16.7%)	1 (20.0%)	3 (37.5%)	
	Numerous		11 (3.7%)	3 (12.5%)	4 (33.3%)	2 (40.0%)	2 (25.0%)	
Urine bilirubin (negative) (*n*, %)	Negative	49	47 (15.7%)	24 (100.0%)	11 (91.7%)	5 (100.0%)	7 (87.5%)	0.255102
	Available		2 (0.7%)	0 (0.0%)	1 (8.3%)	0 (0.0%)	1 (12.5%)	
Urine urobilinogen (0.0–1.0 EU/dL) (*n*, %)	Normal	49	40 (13.3%)	20 (83.3%)	9 (75.0%)	5 (100.0%)	6 (75.0%)	0.708083
	Abnormal		9 (3.0%)	4 (16.7%)	3 (25.0%)	0 (0.0%)	2 (25.0%)	
Urine nitrites (negative) (*n*, %)	Negative	49	45 (15.0%)	24 (100.0%)	10 (83.3%)	5 (100.0%)	6 (75.0%)	0.054721
	Available		4 (1.3%)	0 (0.0%)	2 (16.7%)	0 (0.0%)	2 (25.0%)	
Urine leuk. esterase (negative) (*n*, %)	Negative	49	34 (11.3%)	19 (79.2%)	7 (58.3%)	5 (100.0%)	3 (37.5%)	0.053237
	Available		15 (5.0%)	5 (20.8%)	5 (41.7%)	0 (0.0%)	5 (62.5%)	

* *p* < 0.05 is statistically significant; ** *p* < 0.01 is statistically very significant; *** *p* < 0.001 is statistically extremely significant. ^i^ The most recent lab results before discharge/decease and after the first dose of tocilizumab for patients who received one dose of tocilizumab. ^ii^ The most recent lab results before discharge/decease and after the second dose of tocilizumab for patients received two doses of tocilizumab. ^iii^ The most recent lab results before discharge/decease and after the third dose of tocilizumab for patients received three doses of tocilizumab. ^iv^ The most recent lab results before discharge/decease and after the fourth dose of tocilizumab for patients who received four doses and more of tocilizumab.

**Table 5:** Observed trends for the short-term effects of different doses of tocilizumab on COVID-19 patients and the comparisons between groups.

Lab Test	Baseline (*n* = 300)		All Patients (*n* = 300)		Patients Discharged Alive (*n* = 237)	
	Trend	*p*-value	Trend	*p*-value	Trend	*p*-value
Carbon dioxide	—	0.884520	—		↑	0.000157 ***
AST	—	0.777216	—		↓	0.004630 **
CRP	—	0.140567	—		↓	0.000001 ***
Hemoglobin	—	0.932202	↓	0.012874 **	—	
Hematocrit	—	0.935646	↓	0.061542	—	
MCV	—	0.008551 **	—		↑	0.002726 **
MCHC	—	0.705016	↓	0.618878	—	
RDW	—	0.714308	—		↑	0.043671 *
Mean platelet volume	—	0.206676	—		↑	0.208165
Platelet distribution width	—	0.309775	—		↑	0.494551
Prothrombin time	—	0.747834	↑	0.479953	—	
D-dimer	—	0.291413	↑	0.004335 **	—	

* *p* < 0.05 is statistically significant; ** *p* < 0.01 is statistically very significant; *** *p* < 0.001 is statistically extremely significant.

## Data Availability

The data will be available from authors upon JHAH institutional approval.

## Supplementary Materials

The following supporting information can be downloaded at: https://www.mdpi.com/article/10.3390/pharmaceutics14030624/s1, Table S1. Clinical effectiveness of tocilizumab in treating patients with COVID–19 in different countries. Table S2. Extracted data from the electronic health record system (EPIC^®^) for COVID–19 patients on tocilizumab. Table S3. List of calculated parameters. Table S4. Clinical &laboratory parameters and outcome measures of COVID–19 patients on tocilizumab (survived = 237 & diseased = 63). Table S5. Univariate binary logistic regression analysis for mortality in COVID–19 patients on tocilizumab (demographical and clinical characteristics, concurred drug administration, and time &tocilizumab related parameters). Table S6. Univariate binary logistic regression analysis for mortality in COVID–19 patients on tocilizumab (laboratory results). Table S7. Correlation matrix for multicollinearity check between different mortality predictors in COVID–19 patients on tocilizumab (demographical and clinical characteristics, concurrent drugs administration, and time and tocilizumab related parameters). Table S8. Correlation matrix for multicollinearity check between different mortality predictors in COVID–19 patients on tocilizumab (lab results). Table S9. Multivariable binary logistic regression models for mortality in COVID–19 patients on tocilizumab (Model set A–all data). Table S10. Multivariable binary logistic regression model for mortality in COVID–19 patients on tocilizumab (Model set B–all data). Table S11. AIC values for multivariable binary logistic regression models for mortality in COVID–19 patients on tocilizumab (Model set A–all data). Table S12. Multivariable binary logistic regression model for mortality in COVID–19 patients on tocilizumab (Model set B–all data without blood phosphate concentration). Table S13. Multivariable binary logistic regression models for mortality in COVID–19 patients on tocilizumab (Model set A–no outliers). Table S14. AIC values for multivariable binary logistic regression models for mortality in COVID–19 patients on tocilizumab (Model set A–no outliers). Table S15. Multivariable binary logistic regression model for mortality in COVID–19 patients on tocilizumab (Model set B–all data– no outliers). Table S16. Multivariate logistic regression models for mortality in COVID–19 patients on tocilizumab (Model set B–no outliers without blood phosphate concentration). Table S17. Clinical and laboratory characteristics of COVID–19 patients who received single dose of tocilizumab and were discharged alive—the most recent lab results before tocilizumab administration vs. the most recent lab results before discharge (patients discharged alive *n* = 133). Table S18. Comparison of the short–term effect of a single dose of tocilizumab on different laboratory parameters (most recent lab results before discharge) of COVID–19 patients who received a single dose of tocilizumab (all patients vs. patients who discharged a life). Table S19. Baseline characteristics of COVID–19 patients who received different doses of tocilizumab categorized based on the number of doses administered to the patient. Table S20. Kaplan–Meir survival analysis (days) post last dose of tocilizumab among different dose groups of COVID–19 patients. Table S21. Short–term effect of different doses of tocilizumab on the laboratory results of COVID–19 (patients discharged a live, *n* = 237). Table S22. Number of patients who had an infection before and after tocilizumab administrations and the number of incidences. Table S23. Number of incidences of infection before and after tocilizumab administrations based on their anatomical locations. Table S24. Types of microorganisms before and after tocilizumab based on their types. Table S25. Detailed microorganisms before and after tocilizumab. Table S26. Detailed microorganisms before and after tocilizumab administration based on their anatomical location.
