# Supplementary material for: Safety of Tocilizumab in COVID-19 Patients and Benefit of Single-Dose: The Largest Retrospective Observational Study
Source: Pharmaceutics. 2022 Mar 11;14(3):624. doi: 10.3390/pharmaceutics14030624 (PMC8953525; doi:10.3390/pharmaceutics14030624)
Supplement: Supplementary file 1 [file pharmaceutics-14-00624-s001.zip › pharmaceutics-1623154-supplementary.pdf]

pharmaceutics

MDPI

Supplementary Materials: Safety of Tocilizumab in COVID–19 Patients and Benefit of Single–Dose: The Largest Retrospective Observational Study

Ayman M. Al–Qaaneh, Fuad H. Al–Ghamdi, Sayed AbdulAzeez and J. Francis Borgio

Table S1. Clinical effectiveness of tocilizumab in treating patients with COVID–19 in different countries.

Study reference	Date	Country	*n*	Outcome		
				Favor	Not favor	No difference
33605118 [1]	January 2021	Saudi Arabia	40	X		
34479079 [2]	September 2021	Saudi Arabia	37		X	
33085857 [3]	December 2020	USA	161			X
34153727 [4]	May 2021	Saudi Arabia	62	X		
33472855 [5]	January 2021	Brazil	65		X	
33080005[6]	January 2021	Italy	60			X
32405160 [7]	May 2020	Italy	62	X		
PMC7548660[8]	October 2020	USA	44	X		
MedRxiv [9]	April 2020	France	30	X		
MedRxiv [10]	May 2020	USA	21	X		
32448770 [11]	June 2020	Italy	51			X
32570043 [12]	May 2020	Italy	42		X	
32838323 [13]	August 2020	USA	210	X		
32691021 [14]	July 2020	India	70	X		
32835257 [15]	June 2020	Italy	179	X		
33169088 [16]	December 2020	Saudi Arabia	61			X
MedRxiv [17]	June 2020	Spain	77	X		
32397399 [18]	May 2020	Italy	21			X
32387320 [19]	August 2020	France	20	X		
33631065 [20]	February 2021	Multinational [a]	353	X		
33631066 [21]	April 2021	Multinational [b]	294			X
33332779 [22]	December 2020	Multinational [c]	249	X (time to mechanical ventilation or death)		Survival
32744399 [23]	August 2021	Sweden	29	X (time on mechanical ventilation and length of hospital stay)		Mortality
33605108 [24]	June 2021	Oman	62	X (ventilator parameter and extubation)	mortality	
33080017 [25]	January 2021	France	64	X (noninvasive ventilation, mechanical ventilation, and 14 days mortality		WHO–CPS scores and 28 days mortality

[a] United Kingdome, Netherlands, Australia, New Zealand, Ireland, Saudi Arabia (n = 83); [b] Canada, Denmark, France, Italy, Germany, Netherlands, Spain, United Kingdom, and the USA; [c] United States, Peru, Brazil, Kenya, South Africa, Mexico.

Table S2. Extracted data from the electronic health record system (EPIC®) for COVID–19 patients on tocilizumab.

**Parameters**	
Demographical data	
Patient age	
Gender	Male
	Female
Nationality	Saudi
	Non–Saudi
Patient weight (kg)	
Body mass index (BMI) (Normal: 18.5–24.9 kg/m^2)	
Smoking status	
Smoking status	Non–smoker
	Former–smoker
	Active–smoker
Clinical characteristics	
Comorbid Conditions	Hypertension
	Cardiac diseases include pericardiac effusion, coronary artery diseases, diastolic dysfunction, congestive heart failure, cardiomyopathy, and other cardiovascular diseases)
	Dyslipidemia
	Diabetes Mellitus include diabetes mellitus type I &II
	Respiratory diseases include asthma, pneumonia, acute respiratory failure, chronic obstructive pulmonary disease, pleural effusion, and other lung diseases.
	Thyroid diseases include hyperthyroidism, hypothyroidism, and other thyroid diseases.
	Kidney diseases include chronic kidney disease and acute kidney disease.
	Arthritis includes arthritis and rheumatoid arthritis
	Sickle cell disease
	Stroke
	Cancer
	Gastrointestinal diseases
	Osteopenia/osteoporosis
	Gout
	Peripheral vascular diseases include peripheral vascular disease and varicose veins.
	Anemia
	Deep venous thrombosis
	Glucose 6 Phosphate Dehydrogenase Deficiency (G6PD)
	Liver diseases include hepatitis (A, B, and C), fatty liver, and liver cirrhosis
	Autoimmune diseases and Immunosuppressed patients
Concurrent drugs administration	
Antiviral drug	Lopinavir/Ritonavir
	Favipiravir
	Oseltamivir
Corticosteroids	Dexamethasone
	Methylprednisolone
	Hydrocortisone
	Prednisone/prednisolone
Hydroxychloroquine	
Hospital related parameters	
Hospital admission date	
Hospital discharge date	
Date of death	
ICU related parameters	
ICU admission (Yes/No)	
ICU admission date	
ICU discharge date	

Tocilizumab related parameters		
Number of tocilizumab doses		
Amount of tocilizumab dose (mg)		
Time and date of the tocilizumab dose		
The location where the tocilizumab dose was administered; ICU or Non–ICU		
Laboratory related parameters		
All included laboratory results at the time of admission till the time of discharge		
Laboratory results		
Test group	Individual test	Normal range
Electrolyte	Sodium	135–145 mEq/L
	Potassium	3.5–5.1 mEq/L
	Calcium	8.6–10.0 mg/dL
	Magnesium	1.7–2.4 mg/dL
	Phosphorus	2.5–4.5 mg/dL
	Chloride	98–107 mEq/L
	Carbon Dioxide	22–28 mEq/L
Chemical profile	Blood Urea Nitrogen (BUN)	6–20 mg/dL
	Creatinine	0.66–1.25 mg/dL
	Blood Glucose Level	73–178 mg/dL
Cardiac profile	Troponin High Sensitive	0.000–0.026 ng/mL
	B–type Natriuretic Peptide	<100 pg/mL
Hepatic profile	Bilirubin, Total	0.2–1.3 mg/dL
	Alkaline Phosphatase (ALP)	53–128 IU/L
	Alanine Aminotransferase (ALT) (SGPT)	<50 IU/L
	Aspartate Aminotransferase (AST) (SGOT)	17–59 IU/L
	Lactate Dehydrogenase (LDH)	120–246 IU/L
Iron/Anemia Profile	Ferritin	21.81–274.66 ng/mL
C–Reactive protein (CRP)	CRP	<1.0 mg/dL
Complete blood count	White Blood Cell (WBC) count	4.0–10.0 K/μL
	Red Blood Corpuscles (RBC) count	4.5–5.9 M/μL
	Hemoglobin concentration	13.5–17.5 g/dL
	Hematocrit	41–53 %
	Mean Corpuscular Volume (MCV)	77–96 fL
	Mean Corpuscular Hemoglobin (MCH)	26–34 pg
	Mean Corpuscular Hemoglobin Concentration (MCHC)	32–36 g/dL
	Red Corpuscular Distribution Width (RDW)	10.9–15.7
	Platelets	150–450 K/μL
	Mean Platelet Volume	9.0–13.0 fL
	Platelets Distribution Width	10.1–16.1 fL
	Neutrophils Absolute	1.8–7.0 K/μL
	Lymphocytes Absolute	0.9–4.9 K/μL
	Monocytes Absolute	0.0–1.0 K/μL
	Eosinophils Absolute	0.0–0.4 K/μL
	Basophils Absolute	0.0–0.1 K/μL
	Band Neutrophil Absolute	0.0–0.5 K/μL
Coagulation	Prothrombin Time	9.8–12.7 Secs
	International Normalization Ratio (INR)	1.0–1.2 Ratio
	Activated Partial Thromboplastin Time (APTT)	22–33 Secs
	D–Dimer Quantitative	0.0–0.7 mg/L FEU
Urine analysis	Urine Specific Gravity	1.000–1.030
	Urine pH	4.5–8.0
	Urine Protein	Negative
	Urine Glucose	Negative
	Urine Ketone	Negative
	Urine Blood	Negative
	Urine Bilirubin	Negative

	Urine Urobilinogen	0.0–1.0 EU/dL
	Urine Nitrites	Negative
	Urine Leukocyte Esterase	Negative

Table S3. List of calculated parameters.

**Parameters**
Demographical Calculated parameters
➢ **Body mass index groups**:<br>Body mass index values are collapsed and grouped into one of the main BMI groups based on the Centers for Disease Control and Prevention (CDC) classification [26] as below:<br>o Underweight <18.5 kg/m²<br>o Normal weight 18.5–24.9 kg/m²<br>o Overweight 25.0–29.9 kg/m²<br>o Obese >30.0 kg/m²
Clinical calculated parameters
➢ **Number of comorbidities:**<br>Patients are classified into four groups based on the number of comorbidities they had before hospital admission:<br>o Patients have no comorbidity<br>o Patients have one comorbidity<br>o Patients have two comorbidities<br>o Patients have three and more comorbidities
Concurrent drugs calculated parameters
Patients are classified into three groups based on **co–administered medications during their COVID–19 admission**<br>o Patients received at least one type of antiviral drugs<br>o Patients received at least one type of corticosteroids<br>o Patients received Hydroxychloroquine
Hospital and ICU calculated parameters
➢ **Days to ICU admission** for patients admitted to ICU: ICU admission date – Hospital admission date<br>➢ **Days to death** for deceased patients: Death date – Hospital admission date<br>➢ **Total ICU length of stay** for patients admitted to ICU: ICU discharge date–ICU admission date<br>➢ **Hospital length of stay**: Hospital discharge date – Hospital admission date
Tocilizumab calculated parameters
➢ **Days to the first dose of tocilizumab**: Date of the first dose of tocilizumab – Hospital admission date<br>➢ **Days from ICU admission to the first dose of tocilizumab** for patients admitted to ICU: Date of the first dose of tocilizumab – ICU admission date<br>➢ **Hospital length of stay after the nth dose of tocilizumab**: Hospital discharge date – Date of nth dose of tocilizumab<br>➢ **ICU length of stay after the nth dose of tocilizumab** for patients received the nth dose of tocilizumab in ICU or admitted to ICU after the nth dose of tocilizumab:<br>o ICU discharge date – date of the nth dose of tocilizumab; for patients who received the nth dose of tocilizumab in ICU<br>o ICU discharge date – ICU admission date for; patients admitted to ICU after the nth dose of tocilizumab<br>➢ **Days to death after the nth dose of tocilizumab**: Date of death – date of the nth dose of tocilizumab<br>➢ **Days to the next dose of tocilizumab:** date of the n'th+1 dose of tocilizumab – date of the nth dose of tocilizumab<br>➢ **Days between the first and third dose of tocilizumab** for patients received three doses of tocilizumab: Date of the third dose of tocilizumab – date of the first dose of tocilizumab<br>➢ **Total mg of tocilizumab:** summation of all milligrams of tocilizumab administered by the patient during his/her COVID–19 admission<br>➢ **Total mg of tocilizumab in groups**<br>Patients are classified into four groups based on the total mg of tocilizumab administered:<br>o Patients received a total dose of tocilizumab < 800 mg<br>o Patients received a total dose of tocilizumab 801–1600 mg<br>o Patients received a total dose of tocilizumab 1601–2400 mg<br>o Patients received a total dose of tocilizumab >2400 mg<br>➢ **Total mg/kg of tocilizumab** received by the penitent: Total mg of tocilizumab received by the patient divided by the patient actual body weight<br>➢ **Total mg/kg of tocilizumab in groups**<br>Patients are classified into four groups based on the total mg/kg of tocilizumab administered:<br>o Patients received a total dose of tocilizumab <10 mg/kg<br>o Patients received a total dose of tocilizumab 10.1–21 mg/kg<br>o Patients received a total dose of tocilizumab 21.1–32 mg/kg<br>o Patients received a total dose of tocilizumab > 32 mg/kg
Laboratory calculated parameters
➢ Patients are classified into two groups based on their **urine protein**, **urine glucose**, **urine ketone**, **urine bilirubin**, **urine urobilinogen**, **urine nitrites**, and **urine leukocyte esterase**: negative or available.

➢	Patients are classified into four groups based on the **amount of blood in their urine sample**: negative, trace, moderate, or numerous.

Table S4. Clinical &laboratory parameters and outcome measures of COVID–19 patients on tocilizumab (survived= 237 & diseased= 63).

Parameters		Total Patients' Count (%), Mean (SD), or Median (IQR)	Survivors' Count (%), Mean (SD), or Median (IQR)	Non–survivors' Count (%), Mean (SD), or Median (IQR)	*p*–value
Number of patients (*n*, %)		300 (100%)	237 (79%)	63 (21%)	0.020932 *[i]
Mortality for ICU patients					
Number of patients admitted to ICU (*n*, %)		161 (100%)	99 (61.5%)	62 (38.5%)	0.042259 *[ii]
Demographical Characteristics					
Age (year) median (IQR)		63 (55.00–71.00)	60.00 (53.50–68.00)	70.00 (64.00–79.00)	1.3416×10^{-8} ***
Gender	Male (*n*, %)	196 (65.3%)	157 (66.2%)	39 (61.9%)	0.552735
	Female (*n*, %)	104 (34.7%)	80 (33.8%)	24 (38.1%)	
Nationality	Saudi (*n*, %)	281 (93.7%)	220 (93.2%)	61 (96.8%)	0.381512
	Non–Saudi (*n*, %)	18 (6.0%)	16 (6.8%)	2 (3.2%)	
Weight (kg) median (IQR)		83.00 (72.23–95.75)	83.00 (73.10–96.00)	82.70 (70.70–94.50)	0.598736
Body mass index (kg/m^2) median (IQR))		31.36 (27.43–35.76)	31.26 (27.52–35.63)	31.63 (27.33–36.33)	0.968066
Body Mass Index category	Underweight (<18.5) (*n*, %)	3 (1.0%)	3 (1.3%)	0 (0.0%)	0.060835
	Normal weight (18.5–24.9) (*n*, %)	27 (9.0%)	16 (6.8%)	11 (17.5%)	
	Overweight (25.0–29.9) (*n*, %)	94 (31.3%)	78 (32.9%)	16 (25.4%)	
	Obese (>35.0) (*n*, %)	176 (58.7%)	140 (59.1%)	36 (57.1%)	
Smoking status	Non–smoker (*n*, %)	235 (78.3%)	183 (77.2%)	52 (82.5%)	0.402420
	Former–smoker (*n*, %)	39 (13.0%)	34 (14.3%)	5 (7.9%)	
	Active–smoker (*n*, %)	26 (8.8%)	20 (8.6%)	6 (9.5%)	
Clinical Characteristics					
Number of Comorbidities	0 (*n*, %)	73 (24.3%)	64 (27%)	9 (14.3%)	0.000083 ***
	1 (*n*, %)	68 (22.7%)	57 (24.1%)	11 (17.5%)	
	2 (*n*, %)	60 (20.0%)	53 (22.4%)	7 (11.1%)	
	3 or more (*n*, %)	99 (33.0%)	63 (26.6%)	36 (57.1%)	
Comorbid Conditions	Hypertension (*n*, %)	94 (31.3%)	63 (26.6%)	31 (49.2%)	0.001176 **
	Cardiac diseases (*n*, %)	47 (15.7%)	23 (9.7%)	24 (38.1%)	4.3734×10^{-7} ***
	Dyslipidemia (*n*, %)	63 (21.0%)	44 (18.6%)	19 (30.2%)	0.055286
	Diabetes mellitus (*n*, %)	153 (51.0%)	113 (47.7%)	40 (63.5%)	0.033037 *
	Respiratory diseases (*n*, %)	23 (7.7%)	17 (7.2%)	9 (9.5%)	0.593632
	Thyroid diseases (*n*, %)	21 (7.0%)	16 (6.8%)	5 (7.9%)	0.781515
	Kidney diseases (*n*, %)	21 (7.0%)	13 (5.5%)	8 (12.7%)	0.054929
	Arthritis (*n*, %)	32 (10.7%)	20 (8.4%)	12 (19%)	0.021439 *
	Sickle cell disease (*n*, %)	15 (5.0%)	11 (4.6%)	4 (6.3%)	0.527662
	Stroke (*n*, %)	7 (2.3%)	5 (2.1%)	2 (3.2%)	0.640250
	Cancer (*n*, %)	23 (7.7%)	13 (5.5%)	10 (15.9%)	0.013156 *
	Gastrointestinal diseases (*n*, %)	21 (7.0%)	19 (8.0%)	2 (3.2%)	0.267006
	Osteopenia/Osteoporosis (*n*, %)	12 (4.0%)	6 (2.5%)	6 (9.5%)	0.022015 *
	Gout (*n*, %)	2 (0.7%)	2 (0.8%)	0 (0.0%)	1.000000
	Peripheral vascular disease (*n*, %)	3 (1.0%)	3 (1.3%)	0 (0.0%)	1.000000
	Anemia (*n*, %)	17 (5.7%)	10 (4.2%)	7 (5.7%)	0.058971
	Deep venous thrombosis (*n*, %)	4 (1.3%)	2 (0.8%)	2 (3.2%)	0.195364
	G6PD deficiency (*n*, %)	43 (14.3%)	34 (14.3%)	9 (14.3%)	1.000000
	Liver diseases (*n*, %)	6 (2.0%)	3 (1.3%)	3 (4.8%)	0.109455
	Autoimmune diseases/Immunosuppressed (*n*, %)	2 (0.7%)	2 (0.8%)	0 (0.0%)	1.000000
Concurrent Drug Administration					
Patients received at least one type of antiviral drugs (*n*, %)		64 (21.3%)	40 (16.9%)	24 (38.1%)	0.000258 ***
Patient received at least one type of corticosteroids (*n*, %)		298 (99.3)	235 (99.2%)	63 (100.0%)	1.000000
Patient received hydroxychloroquine (*n*, %)		63 (21%)	45 (19.0%)	18 (28.6%)	0.096912
Laboratory Characteristics					
Electrolytes					
Sodium (135–145 mEq/L) median (IQR)		136.00 (133.00–138.00)	136.00 (134.00–138.00)	135.62 (132.00–139.00)	0.528234
Potassium (3.5–5.1 mEq/L) median (IQR)		4.30 (4.10–4.70)	4.30 (4.00–4.70)	4.40 (4.10–4.80)	0.075398
Calcium (8.6–10.0 mg/dL) median (IQR)		8.40 (8.10–8.80)	8.40 (8.10–8.78)	8.40 (8.10–8.80)	0.875897
Magnesium (1.7–2.4 mg/dL) median (IQR)		2.00 (1.80–2.30)	2.00 (1.80–2.20)	2.00 (1.90–2.30)	0.354069
Phosphorus (2.5–4.5 mg/dL) median (IQR)		3.50 (3.00–4.10)	3.40 (3.00–3.80)	3.90 (3.20–5.03)	0.027015*

Chloride (98–107 mEq/L) median (IQR)		101.00 (98.00–103.00)	101.00 (98.00–103.00)	100.00 (96.00–104.00)	0.367070
Carbon Dioxide (22–28 mEq/L) median (IQR)		25.00 (22.00–27.00)	25.00 (23.00–27.00)	24.00 (22.00–28.00)	0.827208
Chemical Profile					
Blood Urea Nitrogen (6–20 mg/dL) median (IQR)		19.00 (14.00–27.00)	17.00 (13.00–24.00)	26.00 (18.00–45.00)	2.3372×10^{-7} ***
Creatinine (0.66–1.25 mg/dL) median (IQR)		0.80 (0.60–1.10)	0.80 (0.60–1.00)	1.00 (0.70–1.70)	0.000085 ***
Blood Glucose Level (73–178 mg/dL) median (IQR)		141.50 (111.75–210.50)	139.00 (110.00–211.00)	149.00 (114.00–205.00)	0.663107
Lactic Acid (0.7–2.1 mmol/L) median (IQR)		1.50 (1.10–2.00)	1.50 (1.10–1.93)	1.45 (1.10–2.00)	0.930610
Cardiac Profile					
Troponin High Sensitive (0.000–0.026 ng/mL) median (IQR)		0.0100 (0.0045–0.0285)	0.0080 (0.0040–0.0195)	0.0190 (0.0095–0.0750)	0.000656 ***
B–type Natriuretic Peptide (<100 pg/mL) median (IQR)		59.00 (20.20–127.20)	44.30 (17.40–107.30)	96.05 (39.70–229.38)	0.009786 **
Hepatic Profile					
Bilirubin, Total (0.2–1.3 mg/dL) median (IQR)		0.5000 (0.4000–0.7000)	0.5000 (0.4000–0.7000)	0.5000 (0.4000–0.8000)	0.568120
Alk. Phosphatase (53 – 128 IU/L) median (IQR)		68.00 (56.00–87.00)	68.00 (55.00–85.00)	70.00 (58.00–97.00)	0.236349
ALT (SGPT) (<50 IU/L) median (IQR)		31.00 (21.00–48.00)	31.00 (21.00–49.00)	29.00 (22.00–39.00)	0.539727
AST (SGOT) (17–59 IU/L) median (IQR)		44.50 (35.00–62.50)	44.00 (34.00–62.00)	46.00 (37.00–77.00)	0.157338
LDH (120–246 IU/L) median (IQR)		356.50 (280.00–462.00)	344.00 (275.00–450.50)	403.00 (337.00–558.00)	0.005640 **
Iron/Anemia Profile					
Ferritin (21.81–274.66 ng/mL) median (IQR)		786.00 (421.00–1538.00)	672.00 (355.00–1283.00)	1205.50 (586.25–2141.50)	0.003288 **
CRP, Quantitative					
CRP, Quantitative (<1.0 mg/dL) median (IQR)		15.60 (6.30–20.40)	16.00 (6.18–20.85)	15.15 (6.53–19.65)	0.701715
Complete Blood Count					
WBC (4.0–10.0 K/μL) median (IQR)		7.45 (5.30–10.10)	7.50 (5.25–10.10)	7.00 (5.30–10.40)	0.834314
RBC (4.5–5.9 M/μL) median (IQR)		4.61 (4.15–5.11)	4.66 (4.22–5.16)	4.42 (3.83–4.76)	0.003536 **
Hemoglobin (13.5–17.5 g/dL) (mean ± SD)		12.31 ±1.89	12.40±1.87	11.97±1.93	0.106621
Hematocrit (41–53 %) (mean ± SD)		38.05 ±5.34	38.34±5.16	36.99±5.89	0.075295
MCV (77–96 fL) median (IQR)		84.00 (77.30–88.38)	83.40 (77.25–87.75)	87.00 (77.30–90.10)	0.011527 *
MCH (26–34 pg) median (IQR)		27.30 (24.80–29.18)	27.00 (24.60–29.10)	27.90 (25.90–29.60)	0.064683
MCHC (32–36 g/dL) median (IQR)		32.40 (31.30–33.40)	32.50 (31.30–33.40)	32.30 (31.20–33.30)	0.779223
RDW (10.9–15.7) median (IQR)		13.75 (12.90–14.80)	13.60 (12.80–14.65)	14.30 (13.00–15.70)	0.008499 **
Platelets (150–450 K/μL) median (IQR)		233.00 (183.00–297.75)	240.00 (188.50–302.50)	209.00 (155.00–259.00)	0.007864 **
Mean Platelet Volume (9.0–13.0 fL) median (IQR)		10.60 (10.00–11.30)	10.60 (9.90–11.30)	10.60 (10.00–11.45)	0.436157
Platelets Distribution Width (10.1–16.1 fL) median (IQR)		12.30 (11.20–14.10)	12.30 (11.15–14.00)	12.70 (11.35–15.20)	0.156941
Neut. Absolute (1.8–7.0 K/μL) median (IQR)		6.05 (3.80–8.50)	6.10 (3.80–8.25)	5.40 (4.00–8.60)	0.771765
Lymph. Absolute (0.9–4.9 K/μL) median (IQR)		0.8000 (0.6000–1.1000)	0.8000 (0.6000–1.1000)	.7000 (0.4000–0.9000)	0.022830 *
Mono. Absolute (0.0–1.0 K/μL) median (IQR)		0.3000 (0.2000–0.5000)	0.3000 (0.2000–0.5000)	0.3000 (0.2000–0.6000)	0.635995
Eos. Absolute (0.0–0.4 K/μL) median (IQR)		0.0000 (0.0000–0.0000)	0.0000 (0.0000–0.0000)	0.0000 (0.0000–0.0000)	0.144171
Baso. Absolute (0.0–0.1 K/μL) median (IQR)		0.0000 (0.0000–0.0000)	0.0000 (0.0000–0.0000)	0.0000 (0.0000–0.0000)	0.144171
Band Neutrophil Absolute (0.0–0.5 K/μL) median (IQR)		0.2700 (0.1300–0.5900)	.2600 (0.1300–0.6150)	.3200 (0.1300–0.5400)	0.801130
Coagulation					
Prothrombin Time (9.8–12.7 Secs) median (IQR)		11.30 (10.80–11.90)	11.30 (10.70–11.83)	11.25 (10.90–12.20)	0.280110
I.N. Ratio (1.0–1.2 Ratio) median (IQR)		1.00 (1.00–1.10)	1.00 (1.00–1.10)	1.00 (1.00–1.10)	0.342053
APTT (22–33 Secs) median (IQR)		30.00 (28.00–33.00)	30.00 (27.00–32.75)	30.00 (28.00–35.00)	0.239214
D–Dimer Quantitative (0.0–0.7 mg/L FEU) median (IQR)		0.73 (0.43–1.41)	0.72 (0.40–1.34)	0.86 (0.59–1.99)	0.003815 **
Urine Analysis					
Urine Specific Gravity (1.000–1.030) median (IQR)		1.0190 (1.0120–1.0250)	1.02 (1.01–1.03)	1.02 (1.01–1.02)	0.114459
Urine pH (4.5–8.0)	5 (*n*, %)	72 (24.0%)	53 (34.6%)	19 (45.2%)	0.284802
	5.5 (*n*, %)	62 (20.7%)	49 (32.0%)	13 (31.0%)	
	6 (*n*, %)	28 (9.3%)	25 (16.3%)	3 (7.1%)	
	6.5 (*n*, %)	14 (4.7%)	13 (8.5%)	1 (2.4%)	
	7 (*n*, %)	15 (5.0%)	10 (6.5%)	5 (11.9%)	
	7.5 (*n*, %)	4 (1.3%)	3 (2.0%)	1 (2.4%)	
Urine Protein (Negative) (*n*, %)	Negative	92 (30.7%)	70 (45.8%)	22 (52.4%)	0.445862
	Available	103 (34.3%)	83 (54.2%)	20 (47.6%)	
Urine Glucose (Negative) (*n*, %)	Negative	138 (46.0%)	110 (71.9%)	28 (66.7%)	0.509288
	Available	57 (19.0%)	43 (28.1%)	14 (33.3%)	
Urine Ketone (Negative) (*n*, %)	Negative	125 (41.7%)	97 (63.4%)	28 (66.7%)	0.695740
	Available	70 (23.3%)	56 (36.6%)	14 (33.3%)	
Urine Blood (Negative) (*n*, %)	Negative	105 (35.0%)	85 (55.6%)	20 (47.6%)	0.522536
	Trace	46 (15.3%)	34 (22.2%)	12 (28.6%)	

	Moderate	36 (12.0%)	29 (19.0%)	7 (16.7%)	
	Numerous	8 (2.7%)	5 (3.3%)	3 (7.1%)	
Urine Bilirubin (Negative) (*n*, %)	Negative	193 (64.3%)	152 (99.3%)	41 (97.6%)	0.385250
	Available	2 (.7%)	1 (0.7%)	1 (2.4%)	
Urine Urobilinogen (0.0–1.0 EU/dL) (*n*, %)	Normal	166 (55.3%)	128 (83.7%)	38 (90.5%)	0.271470
	Abnormal	29 (9.7%)	25 (16.3%)	4 (9.5%)	
Urine Nitrites (Negative) (*n*, %)	Negative	193 (64.3%)	151 (98.7%)	42 (100.0%)	1.000000
	Available	2 (.7%)	2 (1.3%)	0 (0.0%)	
Urine Leuk. Esterase (Negative) (*n*, %)	Negative	182 (60.7%)	143 (93.5%)	39 (92.9%)	1.000000
	Available	13 (4.3%)	10 (6.5%)	3 (7.1%)	
Time–related pentameters					
Hospital related Parameters					
Hospital Length of stay (Days) median (IQR)		14.00 (9.00–21.00)	13.00 (8.00–19.00)	19.00 (14.00–24.00)	0.000001 ***
ICU Related Parameters					
ICU Admission	Not admitted to ICU (*n*, %)	139 (46.3%)	138 (58.2%)	1 (1.6%)	1.1166×10^{-15} ***
	Admitted to ICU (*n*, %)	161 (53.7%)	99 (41.8%)	62 (98.4%)	
For patients admitted to ICU: Time between Hospital admission and ICU admission (Days) median (IQR)		3.00 (1.00–5.00)	3.12±3.14	5.18±4.37	0.001355 **
For All patients, ICU Length of stay (Days) median (IQR)		3.00 (0.00–12.00)	0.00 (0.00–7.00)	14.00 (8.00–21.00)	1.0979×10^{-18} ***
For patients admitted to ICU, ICU Length of stay (Days) median (IQR)		11.00 (6.00–18.00)	9.00 (4.00–16.00)	14.50 (8.00–21.00)	0.001845 **
Tocilizumab Related Parameters					
Number of Tocilizumab Doses					
Number of Tocilizumab Doses (*n*) median (IQR)		2.00 (1.00–2.00)	1.00 (1.00–2.00)	2.00 (2.00–4.00)	1.9903×10^{-7} ***
Number of Tocilizumab Doses	One Dose (*n*, %)	146 (48.7%)	133 (56.1%)	13 (20.6%)	8.9772×10^{-8} ***
	Two Doses (*n*, %)	82 (27.3%)	57 (24.1%)	25 (39.7%)	
	Three Doses (*n*, %)	36 (12.0%)	29 (12.2%)	7 (11.1%)	
	Four Doses or more (*n*, %)	36 (12.0%)	18 (7.6%)	18 (28.6%)	
Total Tocilizumab (mg)					
Total Tocilizumab (mg) median (IQR)		800.00 (400.00–1600.00)	800.00 (400.00–1200.00)	1400.00 (800.00–2400.00)	6.1184×10^{-7} ***
Total Tocilizumab (mg)	<800 (*n*, %)	176 (58.7%)	155 (65.4%)	21 (33.3%)	0.000004 ***
	801–1600 (*n*, %)	69 (23.0%)	47 (19.8%)	22 (34.9%)	
	1601–2400 (*n*, %)	27 (9.0%)	21 (8.9%)	6 (9.5%)	
	>2401 (*n*, %)	28 (9.3%)	14 (5.9%)	14 (22.2%)	
Total Tocilizumab (mg/Kg)[iii]					
Total tocilizumab (mg/kg) median (IQR)		9.5125 (5.88–17.60)	8.40 (5.56–15.95)	16.69 (9.71–29.02)	6.0238×10^{-7} ***
Total tocilizumab (mg/Kg)	<10 mg/kg (*n*, %)	159 (53.0%)	142 (59.9%)	17 (27.0%)	0.000003 ***
	10.1–21 mg/kg (*n*, %)	86 (28.7%)	61 (25.7%)	25 (39.7%)	
	21.1–32 mg/kg (*n*, %)	27 (9.0%)	20 (8.4%)	7 (11.1%)	
	>32 mg/kg (*n*, %)	28 (9.3%)	14 (5.9%)	14 (22.2%)	
Time–Related Parameters (All Patients)					
Time between admission and first dose of tocilizumab (Days) median (IQR)		3.00 (1.00–5.00)	2.00 (1.00–4.00)	4.00 (2.00–7.00)	0.000761 ***
For patients received the first dose of tocilizumab in ICU: Time between ICU admission and the first dose (Days) median (IQR)		1.00 (.00–2.00)	1.00 (0.00–2.00)	1.00 (0.00–3.00)	0.030501 *
ICU length of stay after last dose of tocilizumab (Days) median (IQR)		7.00 (4.00–13.00)	5.00 (3.00–10.00)	8.00 (4.00–15.00)	0.011098 *
Hospital Length of stay after last dose of tocilizumab (Days) median (IQR)		8.00 (5.00–13.00)	8.00 (5.00–13.00)	8.00 (4.00–15.00)	0.875129
Time Related Parameters (Tocilizumab Doses = 2)					
Time between first and second dose of tocilizumab (Days) median (IQR)		3.00 (2.00–5.00)	3.00 (2.00–4.00)	4.00 (1.50–7.00)	0.152134
Time Related Parameters (Tocilizumab Doses = 3)					
Time between first and second dose of tocilizumab (Days) median (IQR)		1.00 (1.00–2.75)	1.00 (1.00–2.00)	1.00 (1.00–5.00)	0.456105
Time between second and third dose of tocilizumab (Days) median (IQR)		1.00 (1.00–3.00)	1.00 (1.00–3.00)	2.00 (1.00–3.00)	0.638697
Time between first and third dose of tocilizumab (Days) median (IQR)		4.00 (2.00–7.00)	3.00 (2.00–6.50)	7.00 (2.00–7.00)	0.269682

* $p < 0.05$ is statistically significant; ** $p < 0.01$ is statistically very significant; *** $p < 0.001$ is statistically extremely significant; [i] Proportion set at the mortality rate of 16.1% based on the highest mortality rate reported for COVID–19 patients on tocilizumab in Saudi Arabia [4]; [ii] The proportion set at the mortality rate of 31.1% based on the highest mortality reported for ICU COVID–19patients on tocilizumab in Saudi Arabia [16]; [iii] Calculated based on the average weight of the KSA adult =75 kg.

Table S5. Univariate binary logistic regression analysis for mortality in COVID–19 patients on tocilizumab (demographical and clinical characteristics, concurred drug administration, and time &tocilizumab related parameters).

**Parameters**		**ODDs ratio (95% CI)**	***p*–value**	**Reference Group**
**Demographical Characteristics**				
Age (year)		1.073 (1.044–1.102)	4.4265×10^{-7} ¥	
Gender	Male	.828 (.466–1.472)	0.520329	Female
Nationality	Saudi	2.218 (0.496–9.912)	0.296941	Non–Saudi
Weight (Kg) median		0.998 (0.983–1.012)	0.742584	
Body mass index (Kg/m^2)		1.010 (0.975–1.046)	0.575646	
Body Mass Index category	Underweight (<18.5)	0.000 (0.000–0.000)	0.999284	Normal weight
	Overweight (25.0–29.9)	0.298 (0.117–0.762)	0.011445¥	
	Obese (>35.0)	0.374 (0.160–0.876)	0.023444¥	
Smoking status	Former–smoker	0.518 (0.193–1.390)	0.191324	Non–smoker
	Active–smoker	1.056 (0.403–2.765)	0.912041	
Clinical Characteristics				
Number of Comorbidities	1	1.372 (.530–3.550)	0.513993	No comorbidity
	2	0.939 (0.328–2.691)	0.907031	
	3 or more	4.063(1.809–9.126)	0.000682 ¥	
Concurrent Drug Administration				
Patients who received at least one type of antiviral drug		3.031 (1.644–5.587)	0.000381 ¥	Patients did not receive antiviral drug
Patients who received at least one type of corticosteroids		4.330×10^{-8} (0.000–.)	0.999442	Patients did not receive corticosteroid
Patients who received hydroxychloroquine		1.707 (0.904–3.223)	0.099353 ¥	Patients did not receive hydroxychloroquine
Time–related pentameters				
Hospital related Parameters				
Hospital Length of stay (Days)		1.022 (1.006–1.038)	0.007188 ¥	
ICU Related Parameters				
ICU Admission	Admitted to ICU	86.424 (11.784–633.822)	0.000012 ¥	Patients not admitted to ICU
For patients admitted to ICU: Time between hospital admission and ICU admission (Days)		1.162 (1.056–1.280)	0.002186 ¥	
For All patients, ICU Length of Stay (Days)		1.094 (1.063–1.127)	2.1868×10^{-9} ¥	
For patients admitted to ICU, ICU Length of Stay (Days)		1.039 (1.011–1.068)	0.005770 ¥	
Tocilizumab Related Parameters				
Number of Tocilizumab Doses				
Number of tocilizumab Doses *(n)*		1.434 (1.213–1.696)	0.000025 ¥	
Number of Tocilizumab Doses	Two Doses (*n*, %)	4.487 (2.144–9.391)	0.000068 ¥	Patients receive one dose
	Three Doses (*n*, %)	2.469 (0.906–6.732)	0.077249 ¥	
	Four Doses or more (*n*, %)	10.231 (4.300–24.340)	1.452×10^{-7} ¥	
Total Tocilizumab (mg)				
Total tocilizumab (mg)		1.000 (1.000–1.001)	0.000071 ¥	
Total Tocilizumab (mg)	801–1600 (*n*, %)	3.455 (1.748–6.828)	0.000361 ¥	Patients receive <800 mg tocilizumab
	1601–2400 (*n*, %)	2.109 (0.764–5.821)	0.149772	
	>2401 (*n*, %)	7.381 (3.093–17.614)	0.000007 ¥	
Total Tocilizumab (mg/kg)				
Total tocilizumab (mg/kg)		1.039 (1.020–1.058)	0.000047 ¥	
Total Tocilizumab (mg/Kg)	10.1–21 mg/kg	3.423 (1.725–6.793)	0.000432 ¥	Patients receive <10 mg/kg tocilizumab
	21.1–32 mg/kg	2.924 (1.079–7.923)	0.034936¥	
	>32 mg/k	8.353 (3.412–20.451)	0.000003 ¥	
Time–Related Parameters (All Patients)				
The time between admission and the first dose of tocilizumab (Days)		1.146 (1.060–1.238)	0.000633 ¥	
For patients who received the first dose of tocilizumab in ICU: Time between ICU admission and the first dose (Days)		1.085 (0.935–1.259)	0.280976	

ICU length of stay after the last dose of tocilizumab (Days)	1.065 (1.021–1.111)	0.003318 ¥
Hospital Length of stay after last dose of tocilizumab (Days)	1.004 (0.981–1.028)	0.722882
Time Related Parameters (Tocilizumab Doses = 2)		
Time between first and second dose of tocilizumab (Days)	1.110 (0.9721.268)	0.124844
Time Related Parameters (Tocilizumab Doses = 3)		
Time between first and second dose of tocilizumab (Days)	1.377 (0.924–2.054)	0.116403
Time between second and third dose of tocilizumab (Days)	1.012 (0.664–1.541)	0.956609
The time between the first and third dose of tocilizumab (Days)	1.208 (0.893–1.635)	0.220946

¥ $p < 0.1$.

Table S6. Univariate binary logistic regression analysis for mortality in COVID–19 patients on tocilizumab (laboratory results).

Parameters		ODDs ratio (95% CI)	*p*–value	Reference Group
Laboratory Characteristics				
Electrolytes				
Sodium (135–145 mEq/L)		0.987 (0.920–1.059)	0.715092	
Potassium (3.5–5.1 mEq/L)		1.602 (0.926–2.770)	0.091881¥	
Calcium (8.6–10.0 mg/dL)		1.000 (0.559–1.790)	0.998777	
Magnesium (1.7–2.4 mg/dL)		2.282 (0.741–7.027)	0.150533	
Phosphorus (2.5–4.5 mg/dL)		1.406 (1.022–1.934)	0.036311¥	
Chloride (98–107 mEq/L)		0.972 (0.925–1.022)	0.269471	
Carbon Dioxide (22–28 mEq/L)		0.991 (0.925–1.061)	0.795548	
Chemical Profile				
Blood Urea Nitrogen (6–20 mg/dL)		1.040 (1.023–1.057)	0.000003¥	
Creatinine (0.66–1.25 mg/dL)		1.243 (1.041–1.485)	0.016337¥	
Blood Glucose Level (73–178 mg/dL))		0.999 (0.996–1.002)	0.545564	
Lactic Acid (0.7–2.1 mmol/L		1.158 (0.788–1.701)	0.455904	
Cardiac Profile				
Troponin High Sensitive (0.000–0.026 ng/mL)		3.375 (0.723–15.752)	0.121689	
B–type Natriuretic Peptide (<100 pg/mL)		1.001 (0.999–1.002)	0.416247	
Hepatic Profile				
Bilirubin, Total (0.2–1.3 mg/dL)		1.601 (0.919–2.789)	0.096617¥	
Alk. Phosphatase (53–128 IU/L)		1.006 (0.996–1.016)	0.273923	
ALT (SGPT) (<50 IU/L)		1.000 (0.991–1.010)	0.926407	
AST (SGOT) (17–59 IU/L)		1.009 (1.002–1.015)	0.007706¥	
LDH (120–246 IU/L)		1.001 (1.000–1.003)	0.027457¥	
Iron/Anemia Profile				
Ferritin (21.81–274.66 ng/mL)		1.000 (1.000–1.000)	0.244069	
CRP, Quantitative				
CRP, Quantitative (<1.0 mg/dL)		0.992 (0.963–1.022)	0.616217	
Complete Blood Count				
WBC (4.0–10.0 K/μL)		1.032 (0.969–1.099)	0.324512	
RBC (4.5–5.9 M/μL)		0.617 (0.423–0.901)	0.012322¥	
Hemoglobin (13.5–17.5 g/dL)		0.885 (0.763–1.027)	0.107485	
Hematocrit (41–53 %)		0.954 (0.905–1.005)	0.076490¥	
MCV (77–96 fL)		1.037 (1.002–1.074)	0.038423¥	
MCH (26–34 pg)		1.077 (0.990–1.173)	0.085384¥	
MCHC (32–36 g/dL)		1.033 (0.865–1.233)	0.722060	
RDW (10.9–15.7)		1.131 (1.020–1.255)	0.019835¥	
Platelets (150–450 K/μL)		0.995 (0.992–0.999)	0.009737¥	
Mean Platelet Volume (9.0–13.0 fL)		1.134 (0.849–1.515)	0.394393	
Platelets Distribution Width (10.1–16.1 fL)		1.081 (0.971–1.204)	0.152812	
Neut. Absolute (1.8–7.0 K/μL)		1.038 (0.967–1.114)	0.300239	
Lymph. Absolute (0.9–4.9 K/μL)		0.411 (0.206–0.821)	0.011710¥	
Mono. Absolute (0.0–1.0 K/μL)		0.925 (0.411–2.084)	0.850539	
Eos. Absolute (0.0–0.4 K/μL)		0.000 (0.000–123.752)	0.144657	
Baso. Absolute (0.0–0.1 K/μL)		0.000 (0.000–827.380)	0.192178	
Band Neutrophil Absolute (0.0–0.5 K/μL)		1.138 (0.582–2.227)	0.705807	
Coagulation				
Prothrombin Time (9.8–12.7 Secs)		1.035 (0.945–1.135)	0.458576	
I.N. Ratio (1.0–1.2 Ratio)		1.342 (0.544–3.312)	0.523519	
APTT (22–33 Secs)		1.047 (0.994–1.102)	0.084205¥	
D–Dimer Quantitative (0.0–0.7 mg/L FEU)		1.069 (1.024–1.117)	0.002427¥	
Urine Analysis				
Urine Specific Gravity (1.000–1.030)		0.000 (0.000–12,872.334)	0.133710	
Urine pH (4.5–8.0)	5.5	0.740 (0.331–1.656)	0.463803	pH =5, the highest frequency pH
	6	0.335 (0.091–1.237)	0.100818	
	6.5	0.215 (0.026–1.753)	0.150945	
	7	1.395 (0.422–4.606)	0.585162	
	7.5	0.930 (0.091–9.491)	0.951051	
Urine Protein (Negative)	Available	0.767 (0.387–1.519)	0.446520	Urine Protein = Negative
Urine Glucose (Negative)	Available	1.279 (0.615–2.660)	0.509882	Urine Glucose = Negative

Urine Ketone (Negative)	Available	0.866 (0.421–1.781)	0.695876	Urine Ketone= Negative
Urine Blood (Negative)	Trace	1.500 (0.661–3.402)	0.331744	Urine Blood = Negative
	Moderate	1.026 (0.393–2.675)	0.958356	
	Numerous	2.550 (0.562–11.566)	0.224955	
Urine Bilirubin (Negative)	Available	3.707 (0.227–60.551)	0.357867	Urine Bilirubin (Negative)
Urine Urobilinogen (0.0–1.0 EU/dL)	Abnormal	0.539 (0.177–1.645)	0.277593	Urine Urobilinogen = Normal (0.0–1.0 EU/dL)
Urine Nitrites (Negative)	Available	0.000 (0.000– .)	0.999441	Urine Nitrites = Negative
Urine Leuk. Esterase (Negative)	Available	1.100 (0.289–4.192)	0.888957	Urine Leuk. Esterase = Negative

¥ $p < 0.1$.

Table S7. Correlation matrix for multicollinearity check between different mortality predictors in COVID–19 patients on tocilizumab (demographical and clinical characteristics, concurrent drugs administration, and time and tocilizumab related parameters) [£].

		Age	BMI	Comorbidities	Antiviral	Hydroxychloroquine	HLS	Being in ICU	H.A to ICU.A	ICULS	NO. OF DOSES	No. of doses four groups	Total mg	Total mg 4 groups	Total mg/kg	Total mg/kg 4 groups	HA to 1st dose	ICULS after the last dose
Age	P. Corr.	1	−0.255	0.315	0.052	−0.041	0.101	0.225	0.078	0.123	0.185	0.209	0.157	0.219	0.226	0.267	0.054	0.004
	N	300	300	300	300	300	300	300	159	300	300	300	300	300	300	300	300	154
BMI	P. Corr.	−0.255	1	−0.090	0.042	0.11	−0.071	−0.014	−0.107	−0.083	−0.019	−0.018	0.049	0.038	−0.088	−0.100	−0.144	−0.221
	N	300	300	300	300	300	300	300	159	300	300	300	300	300	300	300	300	154
Comorbidities	P. Corr.	0.315	−0.090	1	0.011	0.078	0.033	0.209	0.187	0.063	0.1	0.138	0.068	0.105	0.095	0.088	0.117	−0.098
	N	300	300	300	300	300	300	300	159	300	300	300	300	300	300	300	300	154
Antiviral	P. Corr.	0.052	0.042	0.011	1	0.391	0.311	0.435	−0.041	0.335	0.159	0.135	0.145	0.115	0.158	0.162	0.216	0.051
	N	300	300	300	300	300	300	300	159	300	300	300	300	300	300	300	300	154
Hydroxychloroquine	P. Corr.	−0.041	0.11	0.078	0.391	1	0.198	0.233	−0.023	0.145	−0.135	−0.111	−0.160	−0.146	−0.168	−0.162	0.35	−0.020
	N	300	300	300	300	300	300	300	159	300	300	300	300	300	300	300	300	154
HLS	P. Corr.	0.101	−0.071	0.033	0.311	0.198	1	0.42	0.155	0.804	0.452	0.458	0.395	0.402	0.421	0.414	0.319	0.688
	N	300	300	300	300	300	300	300	159	300	300	300	300	300	300	300	300	154
Being in ICU	P. Corr.	0.225	−0.014	0.209	0.435	0.233	0.42	1	−0.153	0.605	0.442	0.494	0.395	0.437	0.408	0.449	0.224	.[a]
	N	300	300	300	300	300	300	300	159	300	300	300	300	300	300	300	300	154
H.A to ICU.A	P. Corr.	0.078	−0.107	0.187	−0.041	−0.023	0.155	−0.153	1	0.016	0.058	0.033	0.015	−0.035	0.049	0.052	0.441	0.043
	N	159	159	159	159	159	159	159	159	159	159	159	159	159	159	159	159	154
ICULS	P. Corr.	0.123	−0.083	0.063	0.335	0.145	0.804	0.605	0.016	1	0.537	0.559	0.495	0.514	0.504	0.514	0.186	0.89
	N	300	300	300	300	300	300	300	159	300	300	300	300	300	300	300	300	154
NO. OF DOSES	P. Corr.	0.185	−0.019	0.1	0.159	−0.135	0.452	0.442	0.058	0.537	1	0.915	0.957	0.899	0.959	0.889	−0.155	0.186
	N	300	300	300	300	300	300	300	159	300	300	300	300	300	300	300	300	154
No. of doses four groups	P. Corr.	0.209	−0.018	0.138	0.135	−0.111	0.458	0.494	0.033	0.559	0.915	1	0.857	0.924	0.865	0.917	−0.131	0.263
	N	300	300	300	300	300	300	300	159	300	300	300	300	300	300	300	300	154
Total mg	P. Corr.	0.157	0.049	0.068	0.145	−0.160	0.395	0.395	0.015	0.495	0.957	0.857	1	0.912	0.96	0.873	−0.187	0.149
	N	300	300	300	300	300	300	300	159	300	300	300	300	300	300	300	300	154
Total mg 4 groups	P. Corr.	0.219	0.038	0.105	0.115	−0.146	0.402	0.437	−0.035	0.514	0.899	0.924	0.912	1	0.893	0.919	−0.170	0.197
	N	300	300	300	300	300	300	300	159	300	300	300	300	300	300	300	300	154
Total mg/kg	P. Corr.	0.226	−0.088	0.095	0.158	−0.168–	0.421	0.408	0.049	0.504	0.959	0.865	0.96	0.893	1	0.914	−0.147	0.167
	N	300	300	300	300	300	300	300	159	300	300	300	300	300	300	300	300	154
Total mg/kg 4 groups	P. Corr.	0.267	−0.100	0.088	0.162	−0.162	0.414	0.449	0.052	0.514	0.889	0.917	0.873	0.919	0.914	1	−0.123	0.212
	N	300	300	300	300	300	300	300	159	300	300	300	300	300	300	300	300	154
HA to 1st dose	P. Corr.	0.054	−0.144	0.117	0.216	0.35	0.319	0.224	0.441	0.186	−0.155	−0.131	−0.187	−0.170	−0.147	−0.123	1	0.057

	N	300	300	300	300	300	300	300	159	300	300	300	300	300	300	300	300	154
ICULS after the last dose	P. Corr.	0.004	−0.221	−0.098	0.051	−0.020	0.688	.[a]	0.043	0.89	0.186	0.263	0.149	0.197	0.167	0.212	0.057	1
	N	154	154	154	154	154	154	154	154	154	154	154	154	154	154	154	154	154

[£] Predictors with absolute correlation coefficients ≥0.7 are considered strongly correlated. [a] Correlation not Calculated

Table S8. Correlation matrix for multicollinearity check between different mortality predictors in COVID–19 patients on tocilizumab (lab results) £.

		Potassium	Phosphorus	BUN	Creatinine	Bilirubin, Total	AST	LDH	RBC	Hematocrit	MCV	MCH	RDW	Platelets	Lymph. Absolute	APTT	D–Dimer
**Potassium**	P. Corr.	1	0.335	0.235	0.16	0.058	0.097	0.106	0.023	0.076	0.086	0.017	0.025	0.109	−0.084	−0.074	0.075
	N	300	117	300	300	282	282	280	300	300	300	300	300	300	300	119	289
**Phosphorus**	P. Corr.	0.335	1	0.537	0.455	−0.098	−0.15	−0.079	−0.072	−0.149	−0.034	−0.089	0.105	0.094	−0.129	0.123	−0.109
	N	117	117	117	117	117	117	116	117	117	117	117	117	117	117	66	115
**BUN**	P. Corr.	0.235	0.537	1	0.727	0.059	0.019	0.046	−0.193	−0.219	0.056	−0.011	0.093	−0.022	−0.175	0.037	0.158
	N	300	117	300	300	282	282	280	300	300	300	300	300	300	300	119	289
**Creatinine**	P. Corr.	0.16	0.455	0.727	1	0.071	0.036	0.006	−0.216	−0.247	0.056	0.022	0.042	−0.074	−0.152	0.18	0.073
	N	300	117	300	300	282	282	280	300	300	300	300	300	300	300	119	289
**Bilirubin, Total**	P. Corr.	0.058	−0.098	0.059	0.071	1	0.226	0.246	−0.22	−0.152	0.182	0.163	0.012	−0.039	−0.134	−0.011	0.258
	N	282	117	282	282	282	282	280	282	282	282	282	282	282	282	115	273
**AST**	P. Corr.	0.097	−0.15	0.019	0.036	0.226	1	0.641	−0.054	0.045	0.144	0.174	−0.086	−0.11	−0.118	0.075	0.122
	N	282	117	282	282	282	282	280	282	282	282	282	282	282	282	115	273
**LDH**	P. Corr.	0.106	−0.079	0.046	0.006	0.246	0.641	1	−0.095	−0.01	0.152	0.164	−0.069	−0.085	−0.213	0.161	0.264
	N	280	116	280	280	280	280	280	280	280	280	280	280	280	280	114	271
**RBC**	P. Corr.	0.023	−0.072	−0.193	−0.216	−0.22	−0.054	−0.095	1	0.765	−0.561	−0.51	0.155	−0.026	0.06	−0.112	−0.145
	N	300	117	300	300	282	282	280	300	300	300	300	300	300	300	119	289
**Hematocrit**	P. Corr.	0.076	−0.149	−0.219	−0.247	−0.152	0.045	−0.01	0.765	1	0.089	0.113	−0.229	−0.089	0.096	−0.182	−0.164
	N	300	117	300	300	282	282	280	300	300	300	300	300	300	300	119	289
**MCV**	P. Corr.	0.086	−0.034	0.056	0.056	0.182	0.144	0.152	−0.561	0.089	1	0.941	−0.5	−0.067	0.008	−0.037	0.043
	N	300	117	300	300	282	282	280	300	300	300	300	300	300	300	119	289
**MCH**	P. Corr.	0.017	−0.089	−0.011	0.022	0.163	0.174	0.164	−0.51	0.113	0.941	1	−0.531	−0.086	0.013	−0.012	0.046
	N	300	117	300	300	282	282	280	300	300	300	300	300	300	300	119	289
**RDW**	P. Corr.	0.025	0.105	0.093	0.042	0.012	−0.086	−0.069	0.155	−0.229	−0.5	−0.531	1	−0.002	−0.082	0.153	0.112
	N	300	117	300	300	282	282	280	300	300	300	300	300	300	300	119	289
**Platelets**	P. Corr.	0.109	0.094	−0.022	−0.074	−0.039	−0.11	−0.085	−0.026	−0.089	−0.067	−0.086	−0.002	1	0.267	−0.009	0.025
	N	300	117	300	300	282	282	280	300	300	300	300	300	300	300	119	289
**Lymph. Absolute**	P. Corr.	−0.084	−0.129	−0.175	−0.152	−0.134	−0.118	−0.213	0.06	0.096	0.008	0.013	−0.082	0.267	1	−0.1	−0.077
	N	300	117	300	300	282	282	280	300	300	300	300	300	300	300	119	289
**APTT**	P. Corr.	−0.074	0.123	0.037	0.18	−0.011	0.075	0.161	−0.112	−0.182	−0.037	−0.012	0.153	−0.009	−0.1	1	0.002
	N	119	66	119	119	115	115	114	119	119	119	119	119	119	119	119	115
**D–Dimer**	P. Corr.	0.075	−0.109	0.158	0.073	0.258	0.122	0.264	−0.145	−0.164	0.043	0.046	0.112	0.025	−0.077	0.002	1
	N	289	115	289	289	273	273	271	289	289	289	289	289	289	289	115	289

[£] Predictors with absolute correlation coefficients ≥0.7 are considered strongly correlated.

Table S9. Multivariable binary logistic regression models for mortality in COVID–19 patients on tocilizumab (Model set A–all data).

Age, BMI–group, number of comorbidities, antiviral drugs administration, hydroxychloroquine administration, hospital length of stay, admission to ICUs, days between hospital admission and the first dose of tocilizumab, number of tocilizumab doses, number of tocilizumab doses–groups, total tocilizumab doses(mg), total tocilizumab doses (mg)–group, total tocilizumab doses (mg/kg), and total tocilizumab doses (mg/kg)–group

Model Number	Tocilizumab Presentation	MODEL		B–value	WALD		ODDs ratio (95% CI)	Constant of the Model	−2 log–likelihood	Nagelkerke R^2	Hosmer And Lemeshow p–vale	Model Accuracy
I	Number of tocilizumab doses	Age		0.078	19.775		1.081 (1.045–1.119)	−10.541	199.613	0.473	0.970	83.7
		Admitted to ICU		4.467	18.123		87.077(11.137–680.806)					
		Days between hospital admission and the first dose		0.102	4.718		1.107 (1.010–1.214)					
II [¥]	Tocilizumab doses in groups	Admitted to ICU		4.169	15.728		64.644 (8.236–507.365)	−10.401	185.330	0.524	0.844	84.7
		Age		0.070	14.223		1.073 (1.034–1.113)					
		No. of tocilizumab doses–groups	Two doses	1.387	12.813	7.363	4.005 (1.470–10.910)					
			Three doses	0.429		0.478	1.536 (.455–5.189)					
			Four doses	1.976		9.422	7.217 (2.043–25.494)					
		Days between hospital admission and the first dose		0.178	9.583		1.195 (1.068–1.338)					
		Hospital length of stay		−0.026–	3.742		.974 (.949–1.000)					
III	(Total tocilizumab in mg) [2]	Age		0.078	19.775		1.081 (1.045–1.119)	−10.541	199.613	0.473	0.970	83.7
		Admitted to ICU		4.467	18.123		87.077(11.137–680.806)					
		Days between hospital admission and the first dose		0.102	4.718		1.107 (1.010–1.214)					
IV	Total mg of tocilizumab in groups	Admitted to ICU		4.129	15.043		62.137 (7.711–500.723)	−10.316	185.711	0.523	0.548	85.7
		Age		0.069	13.766		1.071 (1.033–1.111)					
		Days between hospital admission and the first dose		0.150	8.223		1.162 (1.049–1.287)					
		Total mg of tocilizumab in groups	801–1600 mg	1.016	7.978	4.770	2.763 (1.110–6.878)					
			1601–2400 mg	0.002		0.000	1.002 (.309–3.251)					
			>2401 mg	1.236		4.983	3.443 (1.163–10.193)					
		Number of comorbidities	1	−0.230–	6.441	0.148	0.795 (0.246–2.568)					
			2	−0.882–		1.834	0.414 (0.116–1.483)					

		3 or more	0.443	0.733	1.558 (0.565–4.295)					
V	Total tocilizumab mg/kg	Age	0.078	19.775	1.081 (1.045–1.119)	−10.541	199.613	0.473	0.970	83.7
		Admitted to ICU	4.467	18.123	87.077(11.137–680.806)					
		Days between hospital admission and the first dose	0.102	4.718	1.107 (1.010–1.214)					
VI	Total tocilizumab mg/kg in groups	Age	0.078	19.775	1.081 (1.045–1.119)	−10.541	199.613	0.473	0.970	83.7
		Admitted to ICU	4.467	18.123	87.077(11.137–680.806)					
		Days between hospital admission and the first dose	0.102	4.718	1.107 (1.010–1.214)					

¥ The best model.

Table S10. Multivariable binary logistic regression model for mortality in COVID–19 patients on tocilizumab (Model set B–all data).

Lab results								
**Variables**	**B–value**	**WALD**	**ODDs ratio (95% CI)**	**Constant of the Model**	**−2 log–likeli-hood**	**Nagelkerke *R^2***	**Hosmer And Leme-show p–vale**	**Model Accuracy**
D–Dimer Quantitative	0.117	8.934	1.124 (1.041–1.214)	0.096	121.618	0.572	0.454	76.3
1/ blood phosphate concentration	−4.891–	3.374	0.008 (0.000–1.388)					

Table S11. AIC values for multivariable binary logistic regression models for mortality in COVID–19 patients on tocilizumab (Model set A–all data).

**Model number**	**Calculated AIC€**
I, III, and V–VI	207.613
II ¥	201.33 €
IV	205.711 €

¥ The best model. € Significant differences between models I, III, & V–VI vs. II, and II vs. IV using AIC statistics. No significant difference between models I, III, & V–VI vs. model IV using AIC statistics.

Table S12. Multivariable binary logistic regression model for mortality in COVID–19 patients on tocilizumab (Model set B–all data without blood phosphate concentration).

Lab results								
**MODEL**	**B–value**	**WALD**	**ODDs ratio (95% CI)**	**Constant of the Model**	**−2 log–likeli-hood**	**Nagelkerke R^2**	**Hosmer And Leme-show p–vale**	**Model Accuracy**
RDW	0.230	10.673	1.259 (1.096–1.445)	−8.730–	239.249	0.211	0.449	81.2
MCV	0.051	5.517	1.052 (1.008–1.098)					
AST	0.007	5.227	1.007 (1.001–1.014)					
D–Dimer Quantitative	0.053	5.203	1.055 (1.008–1.104)					
Platelets	−0.004–	4.408	0.996 (0.992–1.000)					
Creatinine	0.173	3.239	1.189 (0.985–1.435)					

Table S13. Multivariable binary logistic regression models for mortality in COVID–19 patients on tocilizumab (Model set A–no outliers).

Age, BMI–group, number of comorbidities, antiviral drugs administration, hydroxychloroquine administration, hospital length of stay, admission to ICUs, days between hospital admission and the first dose of tocilizumab, number of tocilizumab doses, number of tocilizumab doses–groups, total tocilizumab doses(mg), total tocilizumab doses (mg)–group, total tocilizumab doses (mg/kg), and total tocilizumab doses (mg/kg)–group

Model Number	Tocilizumab Presentation	MODEL		B–value	WALD		ODDs ratio (95% CI)	Constant of the Model	−2 log–likelihood	Nagelkerke R^2	Hosmer And Lemeshow p–vale	Model Accuracy
**1**	Number of tocilizumab doses	Age		0.081	15.281		1.085 (1.041–1.130)	−11.324	156.488	0.572	0.796	87.2
		Days between hospital admission and the first dose		0.294	13.651		1.341 (1.148–1.567)					
		Admitted to ICU		3.947	13.533		51.800 (6.323–424.335)					
		Number of comorbidities	1	−0.453–	6.597	0.456	0.636 (0.171–2.368)					
			2	−1.309–		2.888	0.270 (0.060–1.222)					
			3 or more	0.251		0.184	1.285 (.408–4.049)					
		Number of tocilizumab doses		0.435	5.910		1.545 (1.088–2.193)					
		Hospital length of stay		−0.035–	3.624		0.966 (0.932–1.001)					
		Antiviral drug		0.793	2.948		2.209 (0.894–5.461)					
2	Tocilizumab doses in groups	Admitted to ICU		4.251	15.924		70.187 (8.699–566.309)	−11.162	156.833	0.571	0.875	86.8
		Days between hospital admission and the first dose		0.310	15.142		1.363 (1.166–1.593)					
		Age		0.076	13.494		1.079 (1.036–1.123)					
		No. of tocilizumab doses–groups	Two doses	1.629	13.849	8.564	5.101 (1.713–15.190)					
			Three doses	0.653		1.001	1.921 (0.535–6.904)					
			Four doses	2.422		10.608	11.269 (2.624–48.405)					
		Hospital length of stay		−0.041–	4.865		0.960 (0.925–0.995)					
3[¥]	Total tocilizumab in mg	Age		0.082	15.457		1.085 (1.042–1.131)	−11.244	156.380	0.573	0.927	87.2
		Admitted to ICU		3.986	13.821		53.852 (6.584–440.442)					
		Days between hospital admission and the first dose		0.291	13.580		1.338 (1.146–1.562)					
		Number of comorbidities	1	−0.569–	6.455	0.716	0.566 (0.152–2.113)					
			2	−1.272–		2.809	0.280 (0.063–1.241)					
			3 or more	0.206		0.123	1.229 (.389–3.880)					
		Total tocilizumab in mg		0.001	6.102		1.001 (1.000–1.001)					

		Hospital length of stay		–0.030	2.888		0.971 (0.938–1.005)					
		Antiviral drug		0.781	2.885		2.183 (0.887–5.374)					
4	Total mg of tocilizumab in groups	Admitted to ICU		4.172	14.894		64.875 (7.795–39.973)	–10.729	158.273	0.566	0.797	87.2
		Age		0.071	12.665		1.074 (1.033–1.117)					
		Days between hospital admission and the first dose		0.266	12.662		1.305 (1.127–1.511)					
		Number of comorbidities	1	–0.374	7.364	0.330	0.688 (0.192–2.462)					
			2	–1.454		3.633	0.234 (0.052–1.042)					
			3 or more	0.228		0.158	1.256 (0.408–3.862)					
		Total mg of tocilizumab in groups	801–1600 mg	1.028	6.984	4.438	2.796 (1.074–7.275)					
			1601–2400 mg	0.132		0.043	1.141 (0.330–3.941)					
			>2401 mg	1.380		4.212	3.974 (1.064–14.844)					
5	Total tocilizumab mg/kg	Admitted to ICU		4.335	17.020		76.341 (9.734–598.725)	–10.690	165.513	0.539	0.736	85.4
		Age		0.079	15.797		1.082 (1.041–1.125)					
		Days between hospital admission and the first dose		0.213	9.845		1.237 (1.083–1.414)					
		Number of comorbidities	1	–0.354–	6.685	0.308	0.702 (0.201–2.452)					
			2	–1.302–		3.048	0.272 (0.063–1.173)					
			3 or more	0.233		0.172	1.262 (0.420–3.794)					
6	Total tocilizumab mg/kg in groups	Admitted to ICU		4.335	17.020		76.341 (9.734–598.725)	–10.690	165.513	0.539	0.736	85.4
		Age		0.079	15.797		1.082 (1.041–1.125)					
		Days between hospital admission and the first dose		0.213	9.845		1.237 (1.083–1.414)					
		Number of comorbidities	1	–0.354–	6.685	0.308	0.702 (0.201–2.452)					
			2	–1.302–		3.048	0.272 (0.063–1.173)					
			3 or more	0.233		0.172	1.262 (0.420–3.794)					

¥ The best model.

Table S14. AIC values for multivariable binary logistic regression models for mortality in COVID–19 patients on tocilizumab (Model set A–no outliers).

Model Number	Calculated AIC €
I	176.488
II ¥	172.833
III	176.380
IV	178.273
V–VI	179.513

¥ The best model. € Significant differences between models I vs. II, I vs. V–VI, II vs. III, II vs. IV, II vs. V–VI, and III vs. V–VI using AIC statistics. There is no significant difference between models I vs. III, I vs. IV, III vs. IV, and IV vs. V–VI using AIC statistics.

Table S15. Multivariable binary logistic regression model for mortality in COVID–19 patients on tocilizumab (Model set B–all data– no outliers).

Lab Results								
Variables	B–value	WALD	ODDs ratio (95% CI)	Constant of the Model	–2 log–likelihood	Nagelkerke R^2	Hosmer And Leme-show p–vale	Model Accuracy
Blood phosphate concentration	0.801	6.937	2.227 (1.1.227–4.042)	–4.059	90.631	0.134	0.073	78.4

Table S16. Multivariate logistic regression models for mortality in COVID–19 patients on tocilizumab (Model set B–no outliers without blood phosphate concentration).

Lab Results								
MODEL	B–value	WALD	ODDs ratio (95% CI)	Constant of the Model	–2 log–likelihood	Nagelkerke R2	Hosmer And Leme-show p–vale	Model Accuracy
RDW	0.213	5.836	1.237 (1.041–1.470)	–8.745	197.682	0.126	0.350	81.4
Creatinine	0.579	5.747	1.785 (1.111–2.866)					
MCV	0.057	5.422	1.059 (1.009–1.111)					
Platelets	–0.005–	4.140	0.995 (.990–1.000)					

Table S17. Clinical and laboratory characteristics of COVID–19 patients who received single dose of tocilizumab and were discharged alive – the most recent lab results before tocilizumab administration vs. the most recent lab results before discharge (patients discharged alive *n* = 133).

Parameters	Value
Number of patients (*n*, %)	133 (44.3%)
Tocilizumab Related Parameters	
Total Tocilizumab (mg) median (IQR)	400.00 (400.00–600.00)
Total Tocilizumab (mg/kg) (mean ± SD)	5.88 (5.00–7.45)
Demographical Characteristics	
Age median (IQR)	58.00 (53.00–67.00)
Gender	
Male (*n*, %)	84 (63.2%)
Female (*n*, %)	49 (36.8%)
Nationality	
Saudi (*n*, %)	121 (91.0%)
Non–Saudi (*n*, %)	11 (8.3%)
Weight Related Parameters	
Weight median (kg) (IQR)	81.50 (74.25–96.00
Body mass index median (kg/m2) (IQR)	31.24 (27.26–35.16)
Body Mass Index category	
Underweight (<18.5) (*n*, %)	1 (0.8%)
Normal weight (18.5–24.9) (*n*, %)	9 (6.8%)
Overweight (25.0–29.9) (*n*, %)	45 (33.8%)
Obese (>35.0) (*n*, %)	48 (58.6%)
Smoking status	
Non–smoker (*n*, %)	101 (75.9%)
Former–smoker (*n*, %)	21 (15.8%)
Active–smoker (*n*, %)	11 (8.3%)
Clinical Characteristics	
Number of Comorbidities	
0 (*n*, %)	37 (27.8%)
1 (*n*, %)	32 (24.1%)
2 (*n*, %)	32 (24.1%)
3 or more (*n*, %)	32 (24.1%)
Concurrent Drug Administration	
Patients received at least one Antiviral drug (*n*, %)	15 (11.3%)
Patient received at least one type of Corticosteroids (*n*, %)	131 (98.5)
Patient received Hydroxychloroquine (*n*, %)	27 (20.3%)
Time Related Parameters	
Hospital Related Parameters	
Hospital Length of stay median (IQR)	10.00 (6.00–15.00)
ICU Related Parameters	
Not admitted to ICU (*n*, %)	99 (74.4%)
Admitted to ICU (*n*, %)	34 (25.6)
For patients admitted to ICU: Days between Hospital admission and ICU admission median (IQR)	3.00 (.75–4.25)
ICU Length of stay median (IQR)	0.00 (0.00–1.50)
Time Related Parameters–Tocilizumab (All Patients)	
The time between admission and the first dose of Tocilizumab median (IQR)	3.00 (1.00–4.50)
For patients who received the first dose of Tocilizumab in ICU: Time between ICU admission and the first dose median (IQR)	1.00 (0.00–3.00)
ICU length of stay after the last dose of Tocilizumab median (IQR)	5.00 (3.00–9.50)
Hospital Length of stay after the last dose of Tocilizumab median (IQR)	7.00 (5.00–11.00)

Laboratory Characteristics				
Parameter	Most recent lab result before TCZ administration	Most recent Lab results before discharge	*n*	*p*–value
Electrolytes				
Sodium (135–145 mEq/L) median (IQR)	136.00 (134.00–138.00)	135.00 (133.00–137.00)	128	0.116919
Potassium (3.5–5.1 mEq/L (mean ± SD)	4.33±0.46	4.34±0.45	128	0.779753
Calcium (8.6–10.0 mg/dL) (mean ± SD)	8.26±0.68	8.68±0.58↑	31	0.000193 ***
Magnesium (1.7–2.4 mg/dL) median (IQR)	2.10 (1.90–2.20)	2.10 (2.00–2.20)	40	0.144292
Phosphorus (2.5–4.5 mg/dL) median (IQR)	3.40 (3.00–3.80)	3.85 (3.40–4.50)↑	30	0.009330 **
Chloride (98–107 mEq/L) median (IQR)	101.00 (98.00–104.00)	101.00 (98.00–103.00)	128	0.839649
Carbon Dioxide (22–28 mEq/L) median (IQR)	25.00 (23.00–27.00)	26.00 (24.00–29.00)↑	128	0.000019 ***
Chemical Profile				
Blood Urea Nitrogen (6–20 mg/dL) median (IQR)	17.00 (12.50–23.50)	20.00 (16.00–25.00)↑	128	4.4332×10^{-7} ***
Creatinine (0.66–1.25 mg/dL) median (IQR)	0.8000 (0.6000–0.9000)	0.7000 (0.6000–0.9000)	128	0.840693
Blood Glucose Level (73–178 mg/dL) (mean ± SD)	139.00 (107.00–211.00)	171.00 (105.75–262.75)	7	0.463071
Lactic Acid (0.7–2.1 mmol/L) median (IQR)	1.50 (1.20–2.00)	1.40 (1.10–1.90)	33	0.051830
Cardiac Profile				
Troponin High Sensitive (0.000–0.026 ng/mL) median (IQR)	0.0060 (0.0030–0.0125)	0.0040 (0.0030–0.0140)	11	0.179688
B–type Natriuretic Peptide (<100 pg/mL) median (IQR)	40.70 (17.98–107.15)	48.50 (11.35–133.93)	10	0.343750
Hepatic Profile				
Bilirubin, Total (0.2–1.3 mg/dL) median (IQR)	0.5000 (.4000–0.7000)	0.5000 (0.3000–0.7000)	90	0.105192
Alk. Phosphatase (53–128 IU/L) median (IQR)	67.50 (56.00–87.75)	67.00 (55.50–82.00)	90	0.073140
ALT (SGPT) (<50 IU/L) median (IQR)	32.00 (22.00–49.75)	46.00 (28.75–69.25)↑	90	0.038166 *
AST (SGOT) (17–59 IU/L) median (IQR)	42.50 (33.25–59.00)	40.50 (27.00–53.00)↓	90	0.003996 **
LDH (120–246 IU/L) median (IQR)	316.00 (261.00–407.00)	294.00 (226.75–373.75)↓	89	0.010959 *
Iron/Anemia Profile				
Ferritin (21.81–274.66 ng/mL) median (IQR)	636.00 (349.50–1212.25)	678.50 (358.00–1084.25)	32	0.719438
CRP, Quantitative				
CRP, Quantitative (<1.0 mg/dL) median (IQR)	13.90 (5.73–19.23)	1.40 (.60–2.50)↓	124	2.299×10^{-28} ***
Complete Blood Count				
WBC (4.0–10.0 K/μL) median (IQR)	7.30 (5.35–10.10)	7.40 (5.20–9.70)	131	0.575072
RBC (4.5–5.9 M/μL) median (IQR)	4.79 (4.29–5.21)	4.77 (4.41–5.23)	131	0.250536
Hemoglobin (13.5–17.5 g/dL) median (IQR)	12.30 (11.25–13.70)	12.60 (11.10–13.60)↑	131	0.048373 *
Hematocrit (41–53 %) median (IQR)	38.30 (35.05–42.10)	39.30 (35.80–42.10)↑	131	0.048495 *
MCV (77–96 fL) median (IQR)	82.50 (74.00–87.70)	82.70 (75.50–88.00)	131	0.254213
MCH (26–34 pg) median (IQR)	26.80 (22.95–29.10)	26.90 (23.10–29.20)	131	0.926025
MCHC (32–36 g/dL) median (IQR)	32.40 (31.20–33.35)	32.20 (31.10–33.60)	131	0.407383
RDW (10.9–15.7) median (IQR)	13.80 (12.70–14.80)	13.70 (12.80–15.70)	131	0.174452
Platelets (150–450 K/μL) median (IQR)	242.00 (197.50–321.00)	317.00 (245.00–407.00)↑	131	4.883×10^{-9} ***
Mean Platelet Volume (9.0–13.0 fL) median (IQR)	10.60 (9.70–11.35)	10.40 (9.70–11.10)↓	116	0.005262 **

Platelets Distribution Width (10.1–16.1 fL) median (IQR)		12.30 (10.85–14.50)	12.05 (10.60–13.90)	116	0.227170
Neut. Absolute (1.8–7.0 K/μL) median (IQR)		5.80 (3.85–7.95)	5.00 (3.10–7.10)↓	127	0.031823*
Lymph. Absolute (0.9–4.9 K/μL) median (IQR)		0.9000 (0.6000–1.2000)	1.3000 (.9000–2.0000)↑	127	1.0634×10^{-11} ***
Mono. Absolute (0.0–1.0 K/μL) median (IQR)		0.3000 (0.2000–0.5000)	0.5000 (0.4000–0.8000)↑	127	3.6741×10^{-12} ***
Eos. Absolute (0.0–0.4 K/μL) median (IQR)		0.0000 (0.0000–0.0000)	0.0000 (0.0000–0.1000)↑	126	1.2978×10^{-9} ***
Baso. Absolute (0.0–0.1 K/μL) median (IQR)		0.0000 (0.0000–0.0000)	0.0000 (0.0000–0.0000)	126	0.404873
Band Neutrophil Absolute (0.0–0.5 K/μL) median (IQR)		0.2550 (0.1000–0.5775)	0.2300 (0.1300–0.4625)	21	1.000000
Coagulation					
Prothrombin Time (9.8–12.7 Secs) median (IQR)		11.30 (10.70–11.90)	11.80 (11.17–12.62)	17	0.301758
I.N. Ratio (1.0–1.2 Ratio) median (IQR)		1.00 (1.00–1.10)	1.10 (1.00–1.10)	17	0.218750
APTT (22–33 Secs) median (IQR)		29.00 (27.00–33.00)	27.00 (25.00–28.75)	13	0.092285
D–Dimer Quantitative (0.0–0.7 mg/L FEU) median (IQR)		0.65 (0.36–1.15)	0.79 (0.40–1.40)	104	0.768625
Urine Analysis					
Urine Specific Gravity (1.000–1.030) (mean ± SD)		1.0233± .0121	1.0213± .00300	9	0.688328
Urine pH (4.5–8.0)	5 (*n*, %)			9	0.432768
	5.5 (*n*, %)				
	6 (*n*, %)				
	6.5 (*n*, %)				
	7 (*n*, %)				
	7.5 (*n*, %)				
Urine Protein (Negative) (*n*)	Negative	1	3	9	0.500000
	Available	8	6		
Urine Glucose (Negative) (*n*)	Negative	8	8	9	1.000000
	Available	1	1		
Urine Ketone (Negative) (*n*)	Negative	5	7	9	0.500000
	Available	4	2		
Urine Blood (Negative) (*n*)	Negative			9	0.763025
	Trace				
	Moderate				
	Numerous				
Urine Bilirubin (Negative) (*n*)	Negative	9	9	9	1.000000
	Available	0	0		
Urine Urobilinogen (0.0–1.0 EU/dL) (*n*)	Normal	7	9	9	0.500000
	Abnormal	2	0		
Urine Nitrites (Negative) (*n*)	Negative	9	9	9	1.000000
	Available	0	0		
Urine Leuk. Esterase (Negative) (*n*)	Negative	8	7	9	1.000000
	Available	1	2		

* $p < 0.05$ is statistically significant. ** $p < 0.01$ is statistically very significant. *** $p < 0.001$ is statistically extremely signify

Table S18. Comparison of the short–term effect of a single dose of tocilizumab on different laboratory parameters (most recent lab results before discharge) of COVID–19 patients who received a single dose of tocilizumab (all patients vs. patients who discharged a life).

**Laboratory Characteristics**		
**Parameter**	**Short–term Effect of Single Dose of Tocilizumab on All Patients (*n* = 146)**	**Short–term Effect of Single Dose of Tocilizumab on Patients Discharged Alive (n = 133)**
Electrolytes		
Calcium (8.6–10.0 mg/dL) median (IQR)	↑**N**	↑**N**
Phosphorus (2.5–4.5 mg/dL) median (IQR)	↑**N**	↑**N**
Carbon Dioxide (22–28 mEq/L) median (IQR)	↑**N**	↑**N**
Chemical Profile		
Blood Urea Nitrogen (6–20 mg/dL) median (IQR)	↑**H**	↑**N**
ALT (SGPT) (<50 IU/L) median (IQR)	↑**N**	↑**N**
AST (SGOT) (17–59 IU/L) median (IQR)	↓**N**	↓**N**
LDH (120–246 IU/L) median (IQR)	↓**H**	↓**H**
CRP, Quantitative		
CRP, Quantitative (<1.0 mg/dL) median (IQR)	↓**H**	↓**H**
Complete Blood Count		
Hemoglobin (13.5–17.5 g/dL) median (IQR)	–	↑**N**
Hematocrit (41–53 %) median (IQR)	–	↑**N**
RDW (10.9–15.7) median (IQR)	↑**N**	–
Platelets (150–450 K/µL) median (IQR)	↑**N**	↑**N**
Mean Platelet Volume (9.0–13.0 fL) median (IQR)	–	↓**N**
Neut. Absolute (1.8–7.0 K/µL) median (IQR)	–	↓**N**
Lymph. Absolute (0.9–4.9 K/µL) median (IQR)	↑**N**	↑**N**
Mono. Absolute (0.0–1.0 K/µL) median (IQR)	↑**N**	↑**N**
Eos. Absolute (0.0–0.4 K/µL) median (IQR)	↑**N**	↑**N**

Table S19. Baseline characteristics of COVID–19 patients who received different doses of tocilizumab categorized based on the number of doses administered to the patient.

Parameters		Total Patients	One Dose	Two Doses	Three Doses	Four Or More	*p*–value
Number of patients (*n*, %)		300 (100%)	146 (48.7%)	82 (27.3%)	36 (12.0%)	36 (12.0%)	
Demographical Characteristics							
Age (mean ± SD)		62.00 (55.00–71.00)	58.50 (53.00–69.00)	65.00 (56.00–75.25)	65.00 (59.00–72.75)	66.50 (60.25–73.75)	0.000485 ***
Gender	Male (*n*, %)	196 (65.3%)	93 (63.7%)	55 (67.1%)	24 (66.7%)	24 (66.7%)	0.952658
	Female (*n*, %)	104 (34.7%)	53 (36.3%)	27 (32.9%)	12 (33.3%)	12 (33.3%)	
Nationality	Saudi (*n*, %)	281 (93.7%)	133 (91.7%)	77 (93.9%)	35 (97.2%)	36 (100.0%)	0.290378
	Non–Saudi (*n*, %)	18 (6.0%)	12 (8.3%)	5 (6.1%)	1 (2.8%)	0 (0.0%)	
Weight (kg) median (IQR)		83.00 (72.23–95.75)	81.25 (73.75–96.00)	84.65 (73.13–97.00)	84.10 (73.50–92.38)	81.35 (66.63–93.85)	0.617307
Body mass index (Kg/m^2) median (IQR)		31.36 (27.43–35.76)	31.23 (27.13–35.15)	31.68 (27.42–36.47)	31.55 (28.80–36.95)	31.62 (27.73–36.54)	0.659972
Body Mass Index category	Underweight (<18.5) (*n*, %)	3 (1.0%)	1 (0.7%)	1 (1.2%)	1 (2.8%)	0 (0.0%)	0.889576
	Normal weight (18.5–24.9) (*n*, %)	27 (9.0%)	12 (8.2%)	8 (9.8%)	2 (5.6%)	5 (13.9%)	
	Overweight (25.0–29.9) (*n*, %)	94 (31.3%)	48 (32.9%)	25 (30.5%)	12 (33.3%)	9 (25.0%)	
	Obese (>35.0) (*n*, %)	176 (58.7%)	85 (58.2%)	48 (58.5%)	21 (58.3%)	22 (61.1%)	
Smoking status	Non–smoker (*n*, %)	231 (78.3%)	111 (76.0%)	66 (80.5%)	30 (83.3%)	28 (77.8%)	0.851965
	Former–smoker (*n*, %)	38 (12.9%)	22 (15.1%)	8 (9.8%)	3 (8.3%)	6 (16.7%)	
	Active–smoker (*n*, %)	26 (8.8%)	13 (8.9%)	8 (9.8%)	3 (8.3%)	2 (5.6%)	
Clinical Characteristics							
Number of Comorbidities	0 (*n*, %)	73 (24.3%)	39 (26.7%)	20 (24.4%)	8 (22.2%)	6 (16.7%)	0.254330
	1 (*n*, %)	68 (22.7%)	35 (24.0%)	18 (22.0%)	10 (27.8%)	5 (13.9%)	
	2 (*n*, %)	60 (20.0%)	34 (23.3%)	14 (17.1%)	6 (16.7%)	6 (16.7%)	
	3 or more (*n*, %)	99 (33.0 %)	38 (26.0%)	30 (36.6%)	12 (33.3%)	19 (52.8%)	
Concurrent Drug Administration							
Patients received at least one Antiviral drug (*n*, %)		64 (21.3%)	22 (15.1%)	22 (26.8%)	9 (25.0%)	11 (30.6%)	0.071797
Patient received at least one type of Corticosteroids (*n*, %)		298 (99.3)	144 (98.6%)	82 (100.0%)	36 (100.0%)	36 (100.0%)	0.733066
Patient received Hydroxychloroquine (*n*, %)		63 (21%)	34 (23.3%)	20 (24.4%)	6 (16.7%)	3 (8.3%)	0.177919
Laboratory Characteristics							
Electrolytes							
Sodium (135–145 mEq/L) (mean ± SD)		135.78 ± 3.94	135.99±3.94	135.78±3.91	135.64±4.32	135.08±3.73	0.668427

Potassium (3.5–5.1 mEq/L) (mean ± SD)	4.36 ± 0.51	4.35±0.49	4.39±0.50	4.30±0.64	4.40±0.47	0.771133
Calcium (8.6–10.0 mg/dL) (mean ± SD)	8.40 (8.10–8.80)	8.50 (8.10–8.85)	8.40 (8.20–8.70)	8.30 (7.90–8.50)	8.55 (7.95–8.93)	0.237853
Magnesium (1.7–2.4 mg/dL) (mean ± SD)	2.08 ±0.30	2.08±0.30	2.15±0.31	1.99±0.31	1.97±0.26	0.064780
Phosphorus (2.5–4.5 mg/dL) median (IQR)	3.50 (3.00–4.10)	3.40 (3.00–3.90)	3.50 (3.15–4.45)	2.90 (2.35–4.10)	3.80 (3.50–4.35)	0.161459
Chloride (98–107 mEq/L) median (IQR)	101.00 (98.00–103.00)	101.00 (98.00–104.00)	101.00 (97.00–103.25)	101.50 (99.00–103.75)	100.00 (97.25–102.00)	0.522922
Carbon Dioxide (22–28 mEq/L) median (IQR)	25.00 (22.00–27.00)	25.00 (22.00–27.00)	24.00 (22.00–28.00)	25.00 (23.00–27.75)	25.00 (22.00–27.00)	0.884520
Chemical Profile						
Blood Urea Nitrogen (6–20 mg/dL) median (IQR)	19.00 (14.00–27.00)	17.50 (13.00–25.25)	19.00 (15.00–32.00)	19.00 (14.00–25.75)	23.50 (18.00–35.25)	0.008393 **
Creatinine (0.66–1.25 mg/dL) median (IQR)	0.8000 (0.6000–1.1000)	0.8000 (0.6000–0.9250)	0.9000 (0.7000–1.2000)	0.8000 (0.7250–1.1000)	0.9000 (0.6250–1.3000)	0.015513 *
Blood Glucose Level (73–178 mg/dL) median (IQR)	141.50 (111.75–210.50)	139.50 (108.50–209.75)	143.50 (111.25–194.00)	136.00 (116.50–200.50)	142.50 (120.50–240.50)	0.804366
Lactic Acid (0.7–2.1 mmol/L) median (IQR)	1.50 (1.10–2.00)	1.50 (1.20–2.00)	1.35 (1.00–1.70)	1.50 (1.10–2.13)	1.50 (1.10–2.30)	0.395015
Cardiac Profile						
Troponin High Sensitive (0.000– 0.026 ng/mL) median (IQR)	0.0100 (0.0045–0.0285)	0.0070 (0.0040–0.0180)	0.0130 (0.0050–0.0520)	0.0145 (0.0040–0.0460)	0.0130 (.0093–0.0243)	0.130941
B–type Natriuretic Peptide (<100 pg/mL) median (IQR)	59.00 (20.20–127.20)	49.25 (19.08–118.40)	69.45 (18.33–118.05)	26.25 (15.67–197.58)	103.20 (47.50–198.90)	0.250478
Hepatic Profile						
Bilirubin, Total (0.2–1.3 mg/dL) median (IQR)	0.5000 (0.4000–0.7000)	0.5000 (0.4000–0.7000)	0.5000 (0.4000–0.9000)	0.5000 (0.4000–0.8000)	0.5000 (0.4000–0.7000)	0.817465
Alk. Phosphatase (53–128 IU/L) median (IQR)	68.00 (56.00–87.00)	69.00 (56.00–89.00)	67.00 (54.50–81.50)	67.50 (55.50–82.50)	68.00 (54.50–101.25)	0.455784
ALT (SGPT) (<50 IU/L) median (IQR)	31.00 (21.00–48.00)	32.00 (22.00–49.50)	31.00 (19.00–49.00)	32.00 (22.75–48.25)	29.00 (22.00–40.25)	0.777216
AST (SGOT) (17–59 IU/L) median (IQR)	44.50 (35.00–62.50)	43.00 (34.50–59.00)	46.00 (36.00–71.50)	45.00 (39.75–80.75)	43.00 (33.50–69.75)	0.561416
LDH (120–246 IU/L) median (IQR)	356.50 (280.00–462.00)	325.00 (262.00–415.25)	387.50 (311.25–493.50)	428.50 (326.75–648.75)	379.50 (299.75–612.50)	0.000539 ***
Iron/Anemia Profile						
Ferritin (21.81–274.66 ng/mL) median (IQR)	786.00 (421.00–1538.00)	735.00 (406.75–1415.78)	844.00 (504.00–1634.50)	516.50 (211.75–1158.50)	973.50 (420.25–1610.50)	0.446257
CRP, Quantitative						
CRP, Quantitative (<1.0 mg/dL) median (IQR)	15.60 (6.30–20.40)	13.90 (5.75–20.00)	17.10 (6.60–21.20)	17.90 (7.00–22.40)	16.50 (6.30–20.40)	0.140567
Complete Blood Count						
WBC (4.0–10.0 K/μL) median (IQR)	7.45 (5.30–10.10)	7.40 (5.40–10.13)	7.70 (4.78–10.18)	9.10 (5.80–12.15)	6.05 (4.50–8.95)	0.073328
RBC (4.5–5.9 M/μL) median (IQR)	4.61 (4.15–5.11)	4.74 (4.25–5.23)	4.50 (4.09–5.00)	4.59 (4.19–4.92)	4.47 (4.11–5.11)	0.102481
Hemoglobin (13.5–17.5 g/dL) (mean ± SD)	12.31 ±1.89	12.29±1.80	12.26±2.00	12.32±1.92	12.50±2.01	0.932202
Hematocrit (41–53 %) (mean ± SD)	38.05 ±5.34	38.12±4.94	37.85±5.97	37.84±5.52	38.47±5.42	0.935646

MCV (77–96 fL) median (IQR)		84.00 (77.30–88.38)	82.60 (73.88–87.70)	85.10 (80.48–89.18)	82.45 (78.83–87.20)	86.25 (82.10–88.55)	0.008551 **
MCH (26–34 pg) median (IQR)		27.30 (24.80–29.18)	26.90 (22.98–29.10)	27.60 (26.05–29.40)	27.40 (25.20–28.35)	28.40 (25.33–29.75)	0.041851 *
MCHC (32–36 g/dL) (mean ± SD)		32.32 ±1.58	32.21±1.67	32.40±1.55	32.48±1.22	32.41±1.56	0.705016
RDW (10.9–15.7) median (IQR)		13.75 (2.90–14.80)	13.80 (12.78–15.33)	13.85 (13.00–14.80)	13.60 (12.90–14.63)	13.45 (12.83–14.88)	0.714308
Platelets (150–450 K/μL) median (IQR)		233.00 (183.00–297.75)	245.00 (196.75–321.00)	225.50 ((178.50–290.25)	240.00 (186.75–265.00)	177.00 (144.50–249.50)	0.000858 ***
Mean Platelet Volume (9.0–13.0 fL) median (IQR)		10.60 (10.00–11.30)	10.60 (9.73–11.38)	10.50 (10.10–11.08)	10.80 (10.35–11.30)	11.00 (10.23–11.58)	0.206676
Platelets Distribution Width (10.1–16.1 fL) median (IQR)		12.30 (11.20–14.10)	12.30 (11.10–14.43)	12.10 (11.20–13.48)	12.60 (11.60–13.73)	12.80 (11.45–15.38)	0.309775
Neut. Absolute (1.8–7.0 K/μL) median (IQR)		6.05 (3.80–8.50)	5.95 (3.90–8.27)	6.10 (3.63–8.55)	7.35 (4.30–9.60)	4.65 (2.85–7.33)	0.095240
Lymph. Absolute (0.9–4.9 K/μL) median (IQR)		0.8000 (0.6000–1.1000)	0.8000 (0.6000–1.2000)	0.7000 (0.5000–1.0000)	0.8000 (0.6000–1.0000)	0.8000 (0.5250–0.9750)	0.042841 *
Mono. Absolute (0.0–1.0 K/μL) median (IQR)		0.3000 (0.2000–0.5000)	0.3000 (0.2000–0.5000)	0.4000 (0.2000–0.6000)	0.3000 (0.2000–0.6750)	0.3000 (0.2000–0.5000)	0.409104
Eos. Absolute (0.0–0.4 K/μL) median (IQR)		0.0000 (.0000–0.0000)	0.0000 (0.0000–0.0000)	0.0000 (0.0000–0.0000)	0.0000 (0.0000–0.0000)	0.0000 (0.0000–0.0000)	0.241188
Baso. Absolute (0.0–0.1 K/μL) median (IQR)		0.0000 (.0000–0.0000)	0.0000 (0.0000–0.0000)	0.0000 (0.0000–0.0000)	0.0000 (0.0000–0.0000)	0.0000 (0.0000–0.0000)	0.287612
Band Neutrophil Absolute (0.0–0.5 K/μL) median (IQR)		0.2700 (0.1300–0.5900)	0.3050 (0.1225–0.6050)	0.2500 (0.1300–0.5650)	0.7500 (0.2300–1.4150)	0.1800 (0.1250–0.3000)	0.111886
Coagulation							
Prothrombin Time (9.8–12.7 Secs) median (IQR)		11.30 (10.80–11.90)	11.40 (10.73–11.90)	11.25 (10.90–11.98)	10.90 (10.30–11.80)	11.30 (10.65–11.70)	0.747834
I.N. Ratio (1.0–1.2 Ratio) median (IQR)		1.00 (1.00–1.10)	1.00 (1.00–1.10)	1.00 (1.00–1.10)	1.00 (1.00–1.10)	1.00 (1.00–1.10)	0.868973
APTT (22–33 Secs) median (IQR)		30.00 (28.00–33.00)	29.50 (27.25–34.50)	31.00 (28.00–33.00)	28.00 (28.00–32.50)	29.50 (28.00–32.25)	0.833412
D–Dimer Quantitative (0.0–0.7 mg/L FEU) median (IQR)		0.73 (0.43–1.41)	0.66 (0.37–1.33)	0.83 (0.44–1.72)	0.76 (0.46–1.36)	0.90 (0.51–1.21)	0.291413
Urine Analysis							
Urine Specific Gravity (1.000–1.030) (mean ± SD)		1.0197±.00901	1.0200±.00867	1.0197±.00989	1.0174±.00806	1.0206±.00951	0.585585
Urine pH (4.5–8.0)	5 (*n*, %)	72 (24.0%)	38 (39.2%)	18 (34.6%)	10 (41.7%)	6 (27.3%)	0.925127
	5.5 (*n*, %)	62 (20.7%)	29 (29.9%)	14 (26.9%)	9 (37.5%)	10 (45.5%)	
	6 (*n*, %)	28 (9.3%)	12 (12.4%)	11 (21.2%)	2 (8.3%)	3 (13.6%)	
	6.5 (*n*, %)	14 (4.7%)	9 (9.3%)	3 (5.8%)	1 (4.2%)	1 (4.5%)	
	7 (*n*, %)	15 (5.0%)	7 (7.2%)	5 (9.6%)	2 (8.3%)	1 (4.5%)	
	7.5 (*n*, %)	4 (1.3%)	2 (2.1%)	1 (1.9%)	0 (0.0%)	1 (4.5%)	
Urine Protein (Negative) (*n*, %)	Negative	92 (30.7%)	46 (47.4%)	25 (48.1%)	12 (50.0%)	9 (40.9%)	0.931261
	Available	103 (34.3%)	51 (52.6%)	27 (51.9%)	12 (50.0%)	13 (59.1%)	
	Negative	138 (46.0%)	74 (76.3%)	29 (55.8%)	20 (83.3%)	15 (68.2%)	0.029464 *

Urine Glucose (Negative) (*n*, %)	Available	57 (19.0%)	23 (23.7%)	23 (44.2%)	4 (16.7%)	7 (31.8%)	
Urine Ketone (Negative) (*n*, %)	Negative	125 (41.7%)	59 (60.8%)	32 (61.5%)	18 (75.0%)	16 (72.7%)	0.466107
	Available	70 (23.3%)	38 (39.2%)	20 (38.5%)	6 (25.0%)	6 (27.3%)	
Urine Blood (Negative) (*n*, %)	Negative	105 (35.0%)	49 (50.5%)	32 (61.5%)	13 (54.2%)	11 (50.0%)	0.683917
	Trace	46 (15.3%)	25 (25.8%)	11 (21.2%)	4 (16.7%)	6 (27.3%)	
	Moderate	36 (12.0%)	18 (18.6%)	6 (11.5%)	7 (29.2%)	5 (22.7%)	
	Numerous	8 (2.7%)	5 (5.2%)	3 (5.8%)	0 (0.0%)	0 (0.0%)	
Urine Bilirubin (Negative) (*n*, %)	Negative	193 (64.3%)	96 (99.0%)	52 (100.0%)	23 (95.8%)	22 (100.0%)	0.487179
	Available	2 (.7%)	1 (1.0%)	0 (0.0%)	1 (4.2%)	0 (0.0%)	
Urine Urobilinogen (0.0–1.0 EU/dL) (*n*, %)	Normal	166 (55.3%)	79 (81.4%)	48 (92.3%)	17 (70.8%)	22 (100.0%)	0.008260 **
	Abnormal	29 (9.7%)	18 (18.6%)	4 (7.7%)	7 (29.2%)	0 (0.0%)	
Urine Nitrites (Negative) (*n*, %)	Negative	193 (64.3%)	96 (99.0%)	52 (100.0%)	24 (100.0%)	21 (95.5%)	0.364103
	Available	2 (.7%)	1 (1.0%)	0 (0.0%)	0 (0.0%)	1 (4.5%)	
Urine Leuk. Esterase (Negative) (*n*, %)	Negative	182 (60.7%)	89 (91.8%)	50 (96.2%)	24 (100.0%)	19 (86.4%)	0.205746
	Available	13 (4.3%)	8 (8.2%)	2 (3.8%)	0 (0.0%)	3 (13.6%)	

* $p < 0.05$ is statistically significant; ** $p < 0.01$ is statistically very significant; *** $p < 0.001$ is statistically extremely significant.

Table S20. Kaplan–Meir survival analysis (days) post last dose of tocilizumab among different dose groups of COVID–19 patients.

Number of Tocilizumab Doses	28 Days Mean Survival Time (days) After the Last Dose of Tocilizumab (mean ± Std. Error)	95% Confidence Interval	*p*–value	90 Days Mean Survival Time (days) After the Last Dose of Tocilizumab (mean ± Std. Error)	95% Confidence Interval	*p*–value
One dose	8.77±1.35	(6.11–11.42)	0.750664	12.87±3.09	(6.81–18.93)	0.318854
Two doses	9.75±1.31	(7.18–12.32)		10.60±1.52	(7.62–13.58)	
Three doses	7.17±2.88	(1.52–12.81)		11.71±5.16	(1.60–21.82)	
Four or more doses	7.17±2.88	(5.11–10.73)		18.47±4.52	(9.62–27.32)	
Total patients	8.82±0.78	(7.30–10.34)		13.34±1.63	(10.15–16.54)	

Table S21. Short–term effect of different doses of tocilizumab on the laboratory results of COVID–19 (patients discharged a live, *n* = 237).

Parameters	*n*	Total Patients	One Dose	Two Doses	Three Doses	Four Or More	*p*–value
Number of patients (*n*, %)	237	237 (79%)	133 (91.1%)	57 (69.5%)	29 (80.6%)	18 (50.0%)	
Laboratory Characteristics							
Electrolytes							
Sodium (135–145 mEq/L) (mean ± SD)	230	134.95±2.66	135.41±2.85	134.53±2.10	133.93±2.74	134.61±2.12	0.029862 *
Potassium (3.5–5.1 mEq/L) (mean ± SD)	230	4.32±.46	4.34±0.45	4.35±0.46	4.24±0.49	4.17±0.49	0.371573
Calcium (8.6–10.0 mg/dL) (mean ± SD)	116	8.79±.56	8.69±0.57	8.85±0.47	8.80±0.64	8.98±0.56	0.293032
Magnesium (1.7–2.4 mg/dL) (mean ± SD)	149	2.10 (1.90–2.20)	2.10 (2.00–2.20)	2.10 (1.90–2.20)	2.20 (1.85–2.50)	1.85 (1.70–2.03)	0.005985 **
Phosphorus (2.5–4.5 mg/dL) (mean ± SD)	121	3.97±.80	3.97±0.88	3.81±0.74	4.32±0.62	3.86±0.76	0.151380
Chloride (98–107 mEq/L) (mean ± SD)	230	100.52±4.21	100.63±4.30	100.78±4.14	99.34±4.39	100.78±3.49	0.455094
Carbon Dioxide (22–28 mEq/L) (mean ± SD)	230	27.00 (24.00–29.00)	26.00 (24.00–29.00)	27.00 (24.00–29.00)	28.00 (27.00–32.00)	29.50 (27.50–31.25)	0.000157 ***
Chemical Profile							
Blood Urea Nitrogen (6–20 mg/dL) (mean ± SD)	230	21.00 (17.00–27.00)	20.00 (16.00–25.00)	22.00 (18.00–27.00)	22.00 (19.00–29.00)	19.50 (13.50–36.25)	0.147281
Creatinine (0.66–1.25 mg/dL) (mean ± SD)	230	0.7000 (0.6000–0.9000)	0.70 (0.60–0.90)	0.80 (0.50–1.00)	0.70 (0.70–0.80)	0.60 (0.50–0.83)	0.213064
Blood Glucose Level (73–178 mg/dL) (mean ± SD)	12	171.00 (99.25–252.75)	*n* = 10<br>171.00 (105.75–262.75)	*n* = 1	*n* = 1		Not enough sample size to conduct the test
Lactic Acid (0.7–2.1 mmol/L) (mean ± SD)	93	1.50 (1.15–2.00)	1.40 (1.10–1.90)	1.55 (1.10–2.18)	1.80 (1.30–1.92)	1.50 (1.30–2.03)	0.434713
Cardiac Profile							
Troponin High Sensitive (0.000– 0.026 ng/mL)(mean ±SD)	41	0.0050 (0.0030–0.0185)	0.0040 (0.0030–0.0140)	0.0035 (0.0010–0.0233)	0.0225 (0.0033–0.3823)	0.0110 (0.0058–0.0287)	0.147656
B–type Natriuretic Peptide (<100 pg/mL) (mean ±SD)	62	40.00 (11.10–122.23)	48.50 (11.35–133.93)	14.90 (9.99–81.50)	38.40 (12.00–171.90)	54.95 (16.65–116.98)	0.359876
Hepatic Profile							
Bilirubin, Total (0.2–1.3 mg/dL) (mean ± SD)	182	0.5000 (0.4000–0.7000)	0.5000 (0.3000–0.7000)	0.5000 (0.4000–0.8000)	0.6000 (0.4000–0.8000)	0.5000 (0.3250–0.6000)	0.225059
Alk. Phosphatase (53–128 IU/L) (mean ± SD)	181	65.00 (54.00–80.00)	67.00 (55.50–82.00)	63.00 (54.00–71.75)	61.00 (48.00–79.00)	63.00 (54.00–80.75)	0.299463
ALT (SGPT) (<50 IU/L) (mean ± SD)	181	47.00 (28.50–83.00)	46.00 (28.75–69.25)	56.00 (29.00–103.75)	47.00 (35.00–90.00)	27.50 (20.25–64.00)	0.059131
AST (SGOT) (17–59 IU/L) (mean ± SD)	181	36.00 (26.50–47.00)	40.50 (27.00–53.00)	36.50 (26.00–45.00)	32.00 (27.00–39.00)	27.50 (21.25–35.00)	0.004630 **
LDH (120–246 IU/L) (mean ± SD)	181	293.00 (235.00–365.00)	294.00 (226.75–373.75)	300.00 (255.25–364.00)	270.00 (228.00–298.00)	308.00 (246.25–348.00)	0.479027
Iron/Anemia Profile							
Ferritin (21.81–274.66 ng/mL) (mean ± SD)	105	575.00 (317.50–1030.00)	678.50 (358.00–1084.25)	505.50 (411.50–1131.00)	574.00 (219.00–1274.00)	376.50 (298.50–801.00)	0.396176
CRP, Quantitative							

CRP, Quantitative (<1.0 mg/dL) (mean ± SD)	227	0.80 (0.49–1.90)	1.40 (0.60–2.50)	0.60 (0.49–1.05)	0.49 (0.49–1.40)	0.49 (0.49–0.70)	0.000001 ***
Complete Blood Count							
WBC (4.0–10.0 K/μL) (mean ± SD)	234	7.98±3.43	7.85±3.52	8.26±3.12	8.65±3.35	6.95±3.79	0.354469
RBC (4.5–5.9 M/μL) (mean ± SD)	234	4.73±.77	4.82±0.73	4.68±0.88	4.72±0.63	4.28±0.80	0.039838 *
Hemoglobin (13.5–17.5 g/dL) (mean ± SD)	234	12.55±1.84	12.52±1.87	12.68±2.03	12.80±1.47	11.97±1.54	0.457129
Hematocrit (41–53 %) (mean ± SD)	234	38.93±5.00	38.87±4.93	39.28±5.70	39.83±3.89	36.86±4.58	0.230721
MCV (77–96 fL) (mean ± SD)	234	83.14±8.35	81.43±8.65	84.86±7.68	85.01±6.27	87.31±8.41	0.002726 **
MCH (26–34 pg) (mean ± SD)	234	26.83±3.46	26.27±3.76	27.38±3.03	27.32±2.54	28.36±3.02	0.028848*
MCHC (32–36 g/dL) (mean ± SD)	234	32.18±1.61	32.13±1.78	32.24±1.41	32.10±1.26	32.47±1.37	0.775783
RDW (10.9–15.7) (mean ± SD)	234	13.80 (13.00–15.83)	13.70 (12.80–15.70)	13.75 (13.10–15.67)	13.80 (13.00–14.95)	15.40 (14.60–16.45)	0.043671 *
Platelets (150–450 K/μL) (mean ± SD)	234	305.88±116.92	323.81±114.88	286.84±112.34	317.69±124.60	215.67±86.64	0.001161 **
Mean Platelet Volume (9.0–13.0 fL) (mean ± SD)	220	10.40 (9.83–11.10)	10.40 (9.70–11.10)	10.45 (9.98–11.00)	10.50 (9.80–11.00)	11.40 (10.10–11.95)	0.208165
Platelets Distribution Width (10.1–16.1 fL) (mean ± SD)	220	12.10 (10.80–13.88)	12.05 (10.60–13.90)	12.10 (11.25–13.08)	12.10 (10.70–13.60)	13.90 (10.85–15.45)	0.494551
Neut. Absolute (1.8–7.0 K/μL) (mean ± SD)	230	5.05 (3.10–7.50)	5.00 (3.10–7.10)	5.35 (3.10–8.28)	5.20 (3.45–8.30)	4.25 (2.03–6.00)	0.365829
Lymph. Absolute (0.9–4.9 K/μL) (mean ± SD)	230	1.63±.86	1.54±0.87	1.65±0.81	1.91±0.97	1.73±0.75	0.192007
Mono. Absolute (0.0–1.0 K/μL) (mean ± SD)	230	0.6000 (0.4000–0.8000)	0.5000 (0.4000–0.8000)	0.7000 (0.4000–0.8000)	0.6000 (0.4500–0.8000)	0.6000 (0.4000–0.8250)	0.677961
Eos. Absolute (0.0–0.4 K/μL) (mean ± SD)	230	0.1000 (0.0000–0.2000)	0.0000 (0.0000–0.1000)	0.1000 (0.0000–0.2000)	0.0000 (0.0000–0.2000)	0.1000 (0.0000–0.2000)	0.008098 **
Baso. Absolute (0.0–0.1 K/μL) (mean ± SD)	230	0.0000 (0.0000–0.0000)	0.0000 (0.0000–0.0000)	0.0000 (0.0000–0.1000)	0.0000 (0.0000–0.0500)	0.0000 (0.0000–0.0250)	0.061805
Band Neutrophil Absolute (0.0–0.5 K/μL) (mean ± SD)	77	0.2600 (0.1400–0.5450)	0.2300 (0.1300–0.4625)	0.3400 (0.1275–0.5575)	0.6950 (0.5175–1.0325)	0.2200 (0.1200–0.5850)	0.041337 *
Coagulation							
Prothrombin Time (9.8–12.7 Secs) (mean ± SD)	81	11.70 (11.00–12.35)	11.80 (11.18–12.63)	11.65 (11.00–12.53)	11.55 (10.90–12.20)	11.80 (11.40–12.40)	0.797284
I.N. Ratio (1.0–1.2 Ratio) (mean ± SD)	81	1.10 (1.00–1.10)	1.10 (1.00–1.10)	1.10 (1.00–1.13)	1.00 (1.00–1.10)	1.10 (1.00–1.10)	0.700831
APTT (22–33 Secs) (mean ± SD)	77	26.88±8.06	27.04±2.94	29.35±14.10	25.07±1.33	24.67±2.29	0.257836
D–Dimer Quantitative (0.0–0.7 mg/L FEU) (mean ± SD)	188	0.81 (0.42–1.49)	0.79 (0.40–1.40)	0.63 0(.39–1.22)	1.13 (.046–4.03)	1.32 (0.46–2.36)	0.118139
Urine Analysis							
Urine Specific Gravity (1.000–1.030) (mean ± SD)	37	1.01959±.00971	1.02319±0.00974	1.01763±0.00850	1.01175±0.00544	1.01250±0.00714	0.039138 *
Urine pH (4.5–8.0) 5 (*n*, %)	37	7 (3.0%)	5 (23.8%)	1 (12.5%)	1 (25.0%)	0 (0.0%)	0.646419

	5.5 (*n*, %)		7 (3.0%)	5 (23.8%)	1 (12.5%)	0 (0.0%)	1 (25.0%)	
	6 (*n*, %)		7 (3.0%)	4 (19.0%)	2 (25.0%)	0 (0.0%)	1 (25.0%)	
	6.5 (*n*, %)		11 (4.6%)	4 (19.0%)	4 (50.0%)	1 (25.0%)	2 (50.0%)	
	7 (*n*, %)		5 (2.1%)	3 (14.3%)	0 (0.0%)	2 (50.0%)	0 (0.0%)	
	7.5 (*n*, %)		0 (0.0%)	0 (0.0%)	0 (0.0%)	0 (0.0%)	0 (0.0%)	
Urine Protein (Negative) (*n*, %)	Negative	37	21 (8.9%)	13 (61.9%)	4 (50.0%)	2 (50.0%)	2 (80.0%)	0.953978
	Available		16 (6.8%)	8 (38.1%)	4 (50.0%)	2 (50.0%)	2 (50.0%)	
Urine Glucose (Negative) (*n*, %)	Negative	37	29 (12.2%)	16 (76.2%)	7 (87.5%)	3 (75.0%)	3 (75.0%)	0.930551
	Available		8 (3.4%)	5 (23.8%)	1 (12.5%)	1 (25.0%)	1 (25.0%)	
Urine Ketone (Negative) (*n*, %)	Negative	37	29 (12.2%)	16 (76.2%)	7 (87.5%)	4 (100.0%)	2 (50.0%)	0.374272
	Available		8 (3.4%)	5 (23.8%)	1 (12.5%)	0 (0.0%)	2 (50.0%)	
Urine Blood (Negative) (*n*, %)	Negative	37	15 (6.3%)	8 (38.1%)	4 (50.0%)	1 (25.0%)	2 (50.0%)	0.981251
	Trace		7 (3.0%)	5 (23.8%)	1 (12.5%)	1 (25.0%)	0 (0.0%)	
	Moderate		8 (3.4%)	5 (23.8%)	1 (12.5%)	1 (25.0%)	1 (25.0%)	
	Numerous		7 (3.0%)	3 (14.3%)	2 (25.0%)	1 (25.0%)	1 (25.0%)	
Urine Bilirubin (Negative) (*n*, %)	Negative	37	36 (15.2%)	21 (100%)	7 (87.5%)	4 (100.0%)	4 (100.0%)	0.432432
	Available		1 (.4%)	0 (0.0%)	1 (12.5%)	0 (0.0%)	0 (0.0%)	
Urine Urobilinogen (0.0–1.0 EU/dL) (*n*, %)	Normal	37	32 (13.5%)	17 (81.0%)	7 (87.5%)	4 (100.0%)	4 (100.0%)	1.000000
	Abnormal		5 (2.1%)	4 (19.0%)	1 (12.5%)	0 (0.0%)	0 (0.0%)	
Urine Nitrites (Negative) (*n*, %)	Negative	37	34 (14.3%)	21 (100.0%)	6 (75.0%)	4 (100.0%)	3 (75.0%)	0.055598
	Available		3 (1.3%)	0 (0.0%)	2 (25.0%)	0 (0.0%)	1 (25.0%)	
Urine Leuk. Esterase (Negative) (*n*, %)	Negative	37	29 (12.2%)	18 (85.7%)	5 (62.5%)	4 (100.0%)	2 (50.0%)	0.181398
	Available		8 (3.4%)	3 (14.3%)	3 (37.5%)	0 (0.0%)	2 (50.0%)	

* $p < 0.05$ is statistically significant; ** $p < 0.01$ is statistically very significant; *** $p < 0.001$ is statistically extremely significant; [i] The most recent lab results before discharge/decease and after the first dose of Tocilizumab for patients received one dose of tocilizumab ; [ii] The most recent lab results before discharge/decease and after the second dose of Tocilizumab for patients received two doses of tocilizumab; [iii] The most recent lab results before discharge/decease and after the third dose of Tocilizumab for patients received three doses of tocilizumab; [iv] The most recent lab results before discharge/decease and after the fourth dose of Tocilizumab for patients received four doses and more of tocilizumab.

Table S22. Number of patients who had an infection before and after tocilizumab administrations and the number of incidences.

	**Before Tocilizumab**		**After Tocilizumab**	
	**Number of Patients**	**Number of Incidences**	**Number of Patients**	**Number of Incidences**
0	278	0	237	0
1	21	21	34	34
2	1	2	16	32
3	0	0	6	18
4	0	0	4	16
5	0	0	2	10
6	0	0	1	6
Total	22/300	23	63/300	116

Table S23. Number of incidences of infection before and after tocilizumab administrations based on their anatomical locations.

**Anatomical Location of the Infection**	**Number of Incidences Before Tocilizumab**	**Number of Incidences After Starting Tocilizumab**	**Test**	***p*–value**
Blood	3	22	Sign test	0.007538 **
Respiratory	14	57		0.000004 ***
Urine	6	37		0.000048 ***
Total	23	116		5.7734×10^{-7} ***

* $p < 0.05$ is statistically significant; ** $p < 0.01$ is statistically very significant; *** $p < 0.001$ is statistically extremely significant.

Table S24. Types of microorganisms before and after tocilizumab based on their types.

**Microorganism Type**	**Number of Incidences Before Tocilizumab**	**Number of Incidences After Tocilizumab**
Bacterial infection	14	55
Fungal infection	9	61
Total	23	116

Table S25. Detailed microorganisms before and after tocilizumab.

Microorganism Name	Number of Incidences Before Tocilizumab	Number Of Incidences After Tocilizumab
Acinetobacter baumannii complex		2
Aspergillus niger		1
Aspergillus species		4
Burkholderia cepacia group		2
Candida albicans	4	33
Candida dubliniensis	2	5
Candida glabrata	1	
Candida kefyr		1
Candida krusei		2
Candida lusitaniae		2
Candida parapsilosis		3
Candida tropicalis	2	8
Cryptococcus laurentii		1
Enterobacter cloacae		1
Enterococcus faecalis	1	3
Escherichia coli	3	6
Escherichia coli ESBL producing strain		4
Haemophilus parainfluenzae		1
Klebsiella pneumoniae	2	5
MRSA	4	7
Pseudomonas aeruginosa	2	5
Serratia marcescens		1
Staphylococcus aureus	1	3
Staphylococcus epidermidis	1	9
Staphylococcus haemolyticus		1
Stenotrophomonas maltophilia		5
Yeast		1
Total	23	116

Table S26. Detailed microorganisms before and after tocilizumab administration based on their anatomical location.

Anatomical Location	Microorganism Name	Number of Incidences Before Tocilizumab	Number of Incidences After Tocilizumab
Blood	*Acinetobacter baumannii* complex	–	1
	Burkholderia cepacia group	–	1
	Candida albicans	–	3
	Candida tropicalis	–	1
	Enterococcus faecalis	–	2
	Klebsiella pneumoniae	–	1
	MRSA	–	2
	Serratia marcescens	–	1
	Staphylococcus aureus	1	1
	Staphylococcus haemolyticus	–	1
	Staphylococcus epidermidis	1	6
	Stenotrophomonas maltophilia	–	1
	Pseudomonas aeruginosa	1	1
Respiratory	*Acinetobacter baumannii* complex	–	1
	Aspergillus niger	–	1
	Aspergillus species	–	4
	Burkholderia cepacia group	–	1
	Candida albicans	4	16
	Candida dubliniensis	2	3
	Candida glabrata	1	
	Candida krusei	–	1
	Candida lusitaniae	–	1
	Candida parapsilosis	–	2
	Candida tropicalis	1	3
	Cryptococcus laurentii	–	1
	Enterobacter cloacae	–	1
	Escherichia coli	–	2
	Haemophilus parainfluenzae	–	1
	Klebsiella pneumoniae	1	2
	MRSA	4	5
	Pseudomonas aeruginosa	1	2
	Staphylococcus Aureus	–	2
	Staphylococcus epidermidis	–	3
	Stenotrophomonas maltophilia	–	4
	Yeast	–	1
Urine	*Candida albicans*	–	14
	Candida dubliniensis	–	2
	Candida kefyr	–	1
	Candida krusei	–	1
	Candida lusitaniae	–	1
	Candida parapsilosis	–	1
	Candida tropicalis	1	4
	Enterococcus faecalis	1	1
	Escherichia coli	3	4
	Escherichia coli ESBL producing strain	–	4
	Klebsiella pneumoniae	1	2
	Pseudomonas aeruginosa	–	2

References

1. Al BahraniS.Y.; Al-TawfiqJ.A.; AlshaerA.R.; ShilashA.; AlswefyK.; Al-ZayerR.S.; AbouelelaA.M. A Case Series of Severe Hospitalized COVID-19 Patients Treated with Tocilizumab and Glucocorticoids: A Report from Saudi Arabian Hospital. *J. Epidemiology Glob. Heal.* **2021**, *11*, 233–237, doi:10.2991/jegh.k.210112.001.
2. Assiri, A., et al., COVID–19 related treatment and outcomes among COVID–19 ICU patients: A retrospective cohort study. J Infect Public Health, 2021. 14(9): p. 1274–1278.
3. StoneJ.H.; FrigaultM.J.; Serling-BoydN.J.; FernandesA.D.; HarveyL.; FoulkesA.S.; HorickN.K.; HealyB.C.; ShahR.; BensaciA.M.; et al. Efficacy of Tocilizumab in Patients Hospitalized with Covid-19. *New Engl. J. Med.* **2020**, *383*, 2333–2344, doi:10.1056/nejmoa2028836.
4. Al-BaadaniA.; EltayebN.; AlsufyaniE.; AlbahraniS.; BasheriS.; AlbayatH.; BatubaraE.; BalloolS.; Al AssiriA.; FaqihiF.; et al. Efficacy of tocilizumab in patients with severe COVID-19: Survival and clinical outcomes. *J. Infect. Public Heal.* **2021**, *14*, 1021–1027, doi:10.1016/j.jiph.2021.05.015.
5. VeigaV.C.; PratsJ.A.G.G.; FariasD.L.C.; RosaR.G.; DouradoL.K.; ZampieriF.G.; MachadoF.R.; LopesR.D.; BerwangerO.; AzevedoL.C.P.; et al. Effect of tocilizumab on clinical outcomes at 15 days in patients with severe or critical coronavirus disease 2019: randomised controlled trial. *BMJ* **2021**, *372*, n84, doi:10.1136/bmj.n84.
6. Salvarani, C., et al., Effect of Tocilizumab vs Standard Care on Clinical Worsening in Patients Hospitalized With COVID–19 Pneu-monia: A Randomized Clinical Trial. JAMA Intern Med, 2021. 181(1): p. 24–31.
7. CapraR.; De RossiN.; MattioliF.; RomanelliG.; ScarpazzaC.; SormaniM.P.; CossiS. Impact of low dose tocilizumab on mortality rate in patients with COVID-19 related pneumonia. *Eur. J. Intern. Med.* **2020**, *76*, 31–35, doi:10.1016/j.ejim.2020.05.009.
8. Wadud, N., et al., IMPROVED SURVIVAL OUTCOME IN PATIENTS WITH SARS–COV–2 (COVID–19) ARDS WITH TOCILIZUMAB ADMINISTRATION. Chest, 2020. 158(4): p. A696–A697.
9. Roumier, M., et al., Interleukin–6 blockade for severe COVID–19. medRxiv, 2020: p. 2020.04.20.20061861.
10. Ramaswamy, M., et al., Off–Label Real World Experience Using Tocilizumab for Patients Hospitalized with COVID–19 Disease in a Regional Community Health System: A Case–Control Study. medRxiv, 2020: p. 2020.05.14.20099234.
11. Morena, V.; Milazzo, L.; Oreni, L.; Bestetti, G.; Fossali, T.; Bassoli, C.; Torre, A.; Cossu, M.V.; Minari, C.; Ballone, E.; et al. Off-label use of tocilizumab for the treatment of SARS-CoV-2 pneumonia in Milan, Italy. *Eur. J. Intern. Med.* **2020**, *76*, 36–42, doi:10.1016/j.ejim.2020.05.011.
12. Quartuccio, L.; Sonaglia, A.; McGonagle, D.; Fabris, M.; Peghin, M.; Pecori, D.; De Monte, A.; Bove, T.; Curcio, F.; Bassi, F.; et al. Profiling COVID-19 pneumonia progressing into the cytokine storm syndrome: Results from a single Italian Centre study on tocilizumab versus standard of care. *J. Clin. Virol.* **2020**, *129*, 104444, doi:10.1016/j.jcv.2020.104444.
13. Biran, N.; Ip, A.; Ahn, J.; Go, R.C.; Wang, S.; Mathura, S.; A Sinclaire, B.; Bednarz, U.; Marafelias, M.; Hansen, E.; et al. Tocilizumab among patients with COVID-19 in the intensive care unit: a multicentre observational study. *Lancet Rheumatol.* **2020**, *2*, e603–e612, doi:10.1016/s2665-9913(20)30277-0.
14. Gokhale, Y.; Mehta, R.; Karnik, N.; Kulkarni, U.; Gokhale, S. Tocilizumab improves survival in patients with persistent hypoxia in severe COVID-19 pneumonia. *EClinicalMedicine* **2020**, *24*, 100467, doi:10.1016/j.eclinm.2020.100467.
15. Guaraldi, G.; Meschiari, M.; Cozzi-Lepri, A.; Milic, J.; Tonelli, R.; Menozzi, M.; Franceschini, E.; Cuomo, G.; Orlando, G.; Borghi, V.; et al. Tocilizumab in patients with severe COVID-19: a retrospective cohort study. *Lancet Rheumatol.* **2020**, *2*, e474–e484, doi:10.1016/s2665-9913(20)30173-9.
16. Mady, A., et al., Tocilizumab in the treatment of rapidly evolving COVID–19 pneumonia and multifaceted critical illness: A retro-spective case series. Ann Med Surg (Lond), 2020. 60: p. 417–424.
17. Moreno–García, E., et al., Tocilizumab is associated with reduced risk of ICU admission and mortality in patients with SARS–CoV–2 infection. medRxiv, 2020: p. 2020.06.05.20113738.
18. Colaneri, M.; Bogliolo, L.; Valsecchi, P.; Sacchi, P.; Zuccaro, V.; Brandolino, F.; Montecucco, C.; Mojoli, F.; Giusti, E.M.; Bruno, R.; et al. Tocilizumab for Treatment of Severe COVID-19 Patients: Preliminary Results from SMAtteo COvid19 REgistry (SMACORE). *Microorg.* **2020**, *8*, 695, doi:10.3390/microorganisms8050695.
19. Klopfenstein, T.; Zayet, S.; Lohse, A.; Balblanc, J.-C.; Badie, J.; Royer, P.-Y.; Toko, L.; Mezher, C.; Kadiane-Oussou, N.; Bossert, M.; et al. Tocilizumab therapy reduced intensive care unit admissions and/or mortality in COVID-19 patients. *Médecine et Maladies Infectieuses* **2020**, *50*, 397–400, doi:10.1016/j.medmal.2020.05.001.
20. Gordon, A.C., et al., Interleukin–6 Receptor Antagonists in Critically Ill Patients with Covid–19. N Engl J Med, 2021. 384(16): p. 1491–1502.
21. RosasI.O.; BräuN.; WatersM.; GoR.C.; HunterB.D.; BhaganiS.; SkiestD.; AzizM.S.; CooperN.; DouglasI.S.; et al. Tocilizumab in Hospitalized Patients with Severe Covid-19 Pneumonia. *New Engl. J. Med.* **2021**, *384*, 1503–1516, doi:10.1056/nejmoa2028700.
22. SalamaC.; HanJ.; YauL.; ReissW.G.; KramerB.; NeidhartJ.D.; CrinerG.J.; Kaplan-LewisE.; BadenR.; PanditL.; et al. Tocilizumab in Patients Hospitalized with Covid-19 Pneumonia. *New Engl. J. Med.* **2021**, *384*, 20–30, doi:10.1056/nejmoa2030340.
23. EimerJ.; VesterbackaJ.; SvenssonA.; StojanovicB.; WagrellC.; SönnerborgA.; NowakP. Tocilizumab shortens time on mechanical ventilation and length of hospital stay in patients with severe COVID-19: a retrospective cohort study. *J. Intern. Med.* **2021**, *289*, 434–436, doi:10.1111/joim.13162.
24. KhamisF.; MemishZ.; Al BahraniM.; Al NummaniH.; Al RaisiD.; Al DowaikiS.; ChhetriS.; Al FahdiF.; Al YahyaiM.; PandakN.; et al. The Role of Convalescent Plasma and Tocilizumab in the Management of COVID-19 Infection: A Cohort of 110 Patients from a Tertiary Care Hospital in Oman. *J. Epidemiology Glob. Heal.* **2021**, *11*, 216–223, doi:10.2991/jegh.k.201222.001.

25. Hermine, O., et al., Effect of Tocilizumab vs Usual Care in Adults Hospitalized With COVID–19 and Moderate or Severe Pneumonia: A Randomized Clinical Trial. JAMA Intern Med, 2021. 181(1): p. 32–40.
26. (CDC), C.f.D.C.a.p. Defining Adult Overweight & Obesity. 2021 June 7, 2021 [cited 2021 October 11]; Available from: https://www.cdc.gov/obesity/adult/defining.html.
